# Cyclic Hydroxylamines for Native Residue-Forming Peptide Ligations: Synthesis of Ubiquitin and Tirzepatide

**DOI:** 10.1021/jacs.5c11881

**Published:** 2025-09-11

**Authors:** Jiling Han, Kohtaro Hirao, Toshiki Mikami, Nicolas Y. Nötel, Leonardo L. Seidl, Jeffrey W. Bode

**Affiliations:** Laboratory for Organic Chemistry, Department of Chemistry and Applied Biosciences, ETH Zürich, Zürich 8093, Switzerland

## Abstract

The α-ketoacid-hydroxylamine (KAHA) ligation enables the chemoselective coupling of unprotected peptide segments. The most commonly used hydroxylamine building block, (*S*)-5-oxaproline, yields homoserine residues at ligation sites, limiting applications where the native sequence is essential. To overcome this limitation, we developed cyclic dipeptide-derived hydroxylamine building blocks that enable the formation of canonical amino acids directly under modified KAHA ligation conditions. These building blocks are prepared from dipeptides and are applicable at nonobvious peptide ligation junctions, including Leu–Ile and Lys–Ile. We applied this approach to the synthesis of K48/K63 selectively protected ubiquitin monomers for chemoenzymatic ubiquitin chain formation and the total synthesis of tirzepatide, a GLP-1 receptor agonist peptide therapeutic containing amino-isobutyric acid (Aib) residues and a fatty acid side chain modification. This work establishes a practical approach for KAHA ligation at fully native sites and expands its applicability to the practical synthesis of challenging peptide targets.

## Graphical abstract

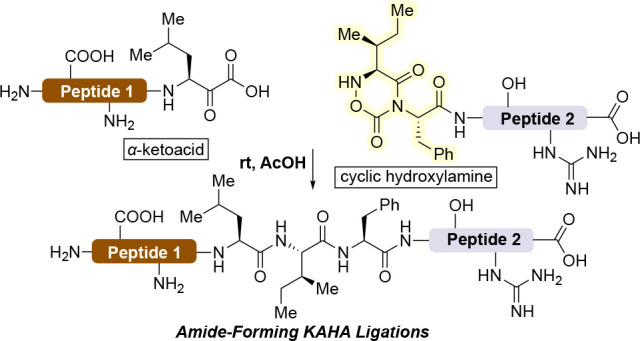

Chemical protein synthesis by chemoselective ligation of peptide segments is a powerful approach to the assembly of atomically tailored protein variants and emerging peptide therapeutics.^1–4^ The transformative native chemical ligation of peptide thioesters and *N*-terminal cysteine residues has been leveraged for the assembly of hundreds of proteins (Figure 1a).^1,5^ Another notable approach is serine/threonine ligation, which enables peptide coupling at Ser/Thr residues using a salicylaldehyde ester linker (Figure 1b).^6,7^ In our own work, we have reported the α-ketoacid–hydroxylamine (KAHA) ligation, which has proved particularly useful for sequences that lack cysteine residues or that are prone to aggregation.^8^ Typical KAHA ligation conditions—DMSO/acidic water or HFIP/AcOH—are well suited for solubilizing even difficult segments,^9^ and the absence of additives or buffers simplifies scale up, as exemplified by its large-scale use for the manufacture of preclinical candidates.^10^

**Figure 1:**
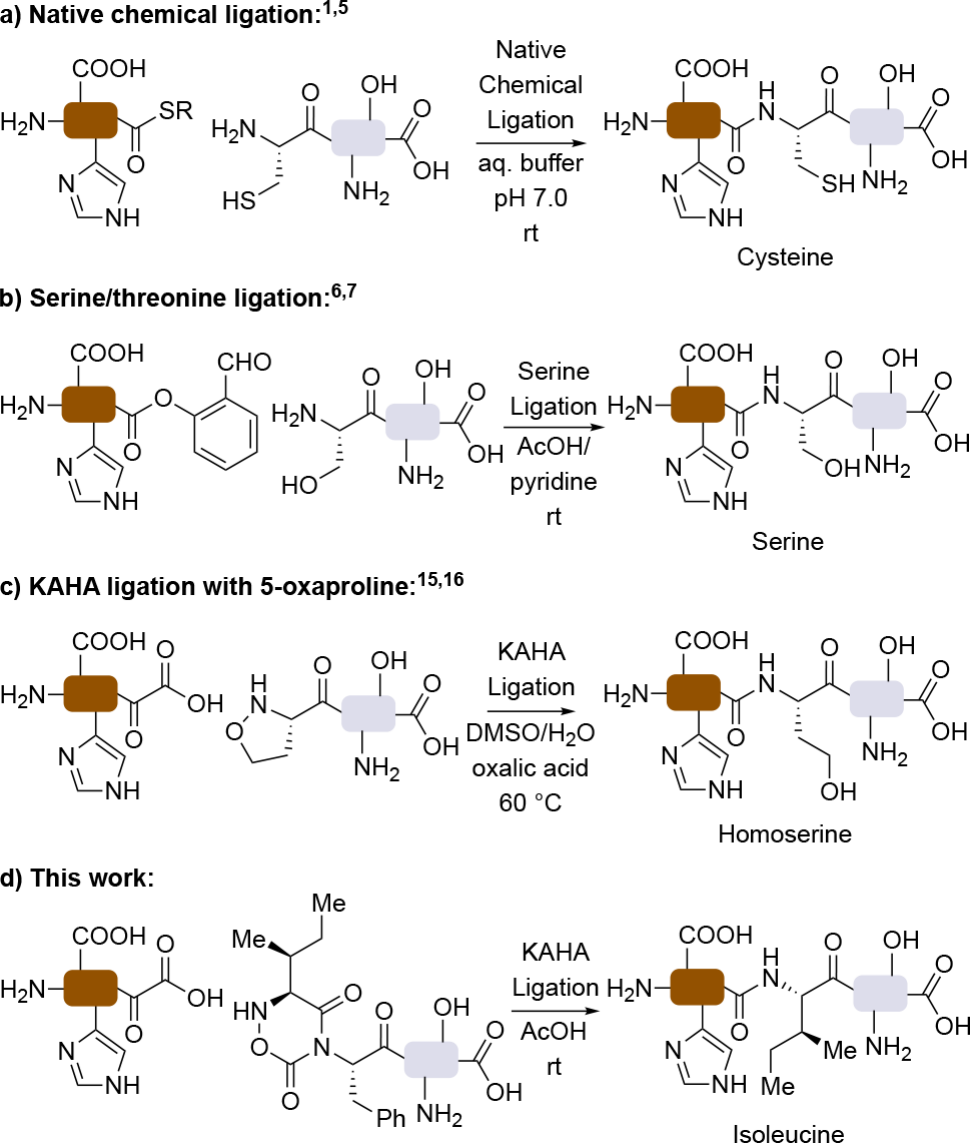
Reported chemical ligation methods and the developed cyclic dipeptide building blocks. (a) Native chemical ligation. (b) Serine/threonine ligation. (c) KAHA ligation using (*S*)-5-oxaproline. (d) KAHA ligation using the developed cyclic dipeptide building block.

Our group has reported robust technologies for installing α-ketoacids at the *C*-terminus of peptide segments on a solid support^11–13^ and from recombinant peptides.^10^ Balancing reactivity and stability of the amino acid derived *N*-terminal hydroxylamines, which are prone to elimination to form imines, has proven more challenging.^14^ The most common implementation of the KAHA ligation employs (*S*)-5-oxaproline as a robust, chemically stable ligation partner,^15^ resulting initially in the formation of an ester before rearrangement to a homoserine residue at the ligation site (Figure 1c).^16^ While this minor deviation from proteogenic amino acids is well tolerated for applications in chemical biology and therapeutic proteins,^17^ we have identified two important cases where preparation of the natural sequence is an absolute requirement. First, ubiquitin (Ub) is a highly conserved, evolutionarily optimized protein whose broader function can be perturbed by even conservative mutations,^18–20^ mandating that synthetic preparation preserve the natural sequence. Second, any peptide API with a predetermined structure obviously does not tolerate deviations from its primary sequence. The emergence and widespread medicinal use of long peptide therapeutics^21^—and associated manufacturing challenges as exemplified by tirzepatide (TZP)^22^—provide a new forum where direct, native amide forming ligations will be of considerable value.

In this report, we disclose the synthesis and utility of cyclic hydroxylamine building blocks suitable for KAHA ligations at unconventional disconnection sites, including Leu–Ile and Lys–Ile, under mild conditions (AcOH, rt) ideal for sustainable peptide production (Figure 1d). We establish the application of these building blocks for the construction of Ub monomers with Aboc-protecting groups selectively installed on K48/K63, useful for our chemoenzymatic platform for ubiquitin chain synthesis.^23^ We also apply these building blocks to a proof-of-principle synthesis of tirzepatide by KAHA ligation between an *N-*terminal segment containing the two amino-isobutyric acid (Aib) residues and a *C-*terminal segment derivatized with both the fatty acid side chain and the cyclic hydroxylamine building block.^24^

Despite numerous advantages of KAHA ligation using (*S*)-5-oxaproline, our group has long sought a general alternative suitable for completely natural ligation sites. Prior work has identified a few hydroxylamines providing the appropriate balance of stability and reactivity, including KAHA ligations that form natural residues from serine-,^25^ threonine-,^26^ and aspartic acid^27^-derived hydroxylamines. However, these monomers cannot easily be produced on a scale. Inspired by work of Gouverneur and Ghosez on the preparation of an unusual class of cyclic hydroxylamines by cycloaddition of azadiene and nitroso compounds,^28,29^ we postulated that cyclization via the backbone amide bond could offer a general approach to KAHA ligations at arbitrary ligation sites (Scheme 1a). Using the Gouverneur–Ghosez route as a roadmap, we successfully prepared small amounts of cyclic hydroxylamines and established that these building blocks could undergo KAHA ligation with simple α-ketoacids. While encouraged by these observations, this cycloaddition route did not provide access to enantiomerically pure material and did not enable attachment to a peptide chain. We therefore turned to a longer—but low-cost and scalable—synthesis by elaborating dipeptide **1** to hydroxylamine **2** using Fukuyama’s method (Scheme 1b).^30^ Conversion of readily produced hydroxylamine **2** to a cyclic building block suitable for incorporation into a solid-supported peptide segment proved to be initially challenging. There are few precedents for cyclization via the backbone amide nitrogen, and the desired six-membered ring was prone to irreversible rearrangement to the more stable five-membered hydroxamic hydantoin,^31^ which could be overcome by appropriate handling of the synthetic intermediates. Selective *N*-protection of peptide-derived hydroxylamines is known to be difficult, the primary reason we had abandoned these precursors more than a decade ago.^14^

**Scheme 1:**
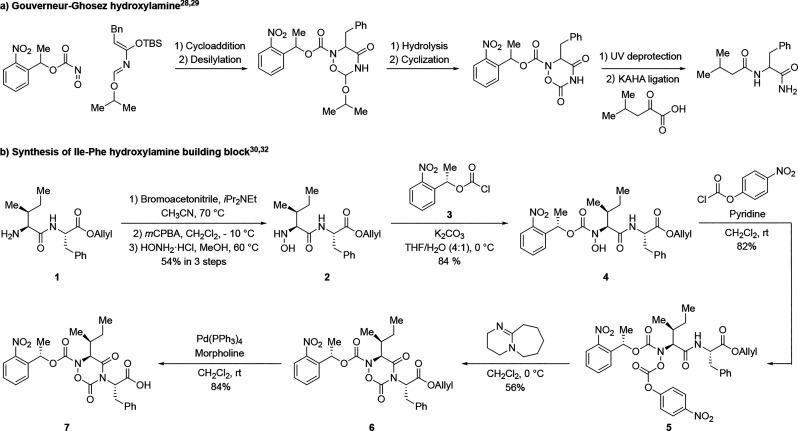
(a) Preliminary Studies on the Preparation of Gouverneur–Ghosez Hydroxylamine Variants and Small-Molecule KAHA Ligation Test; (b) Synthesis of Ile-Phe Cyclic Hydroxylamine Building Block with a Photolabile Protecting Group

We eventually identified a workable and scalable solution to both the production of the cyclic hydroxylamine and its incorporation into synthetic peptide segments following standard solid phase peptide synthesis. Selective *N*-protection of the hydroxylamine nitrogen was accomplished with a photolabile carbamate;^32^ other choices including Boc, Alloc, TMS,^33^ and PMB gave mixtures of *N*-, *O*-, and bis-protection.^34,35^ Photocaged intermediate **4** was cyclized by carbonate formation with *para*-nitrophenyl chloroformate followed by ring closure with DBU in CH_2_Cl_2_ at 0 °C. Clean *O*-allyl deprotection of dipeptide **6** afforded the desired dipeptide **7**.

For the synthesis of K48/K63-Aboc-protected ubiquitin monomers, dipeptide **7** was smoothly coupled to the solid-supported peptide under base-free amide-formation conditions using DIC/Oxyma as coupling reagents in DMF (Scheme 2b).^36^ In its protected form, this cyclic hydroxylamine is stable to all standard peptide manipulations, including resin cleavage (TFA/TIPS/H_2_O, 95:2.5:2.5), reverse phase HPLC, and lyophilization, allowing facile isolation of the otherwise unprotected peptide segment **9a/9b**. Upon photodeprotection—accomplished with a hand-held UV lamp in CH_3_CN/H_2_O—the hydroxylamine can undergo structural isomerization to a five-membered hydroxamic hydantoin as a minor peak adjacent to **9a** (Scheme 2c), a side reaction minimized by dilution to 1 mM and cooling on ice during the deprotection. Working initially on the synthesis of ubiquitin monomers, we were pleased to find that incubation of the hydroxylamine segment **9** **a** **/9b** with *C*-terminal Leu-α-ketoacid segment **8a/8b** bearing a Met1Nle substitution—to avoid oxidation^37^—in AcOH/HFIP at room temperature afforded the desired amide-ligation product (Scheme 2b). Despite coupling two hindered amino acids, leucine at the α-ketoacid and isoleucine at the hydroxylamine, these ligations proceed efficiently in good yield with the unprotected peptide segments.

**Scheme 2:**
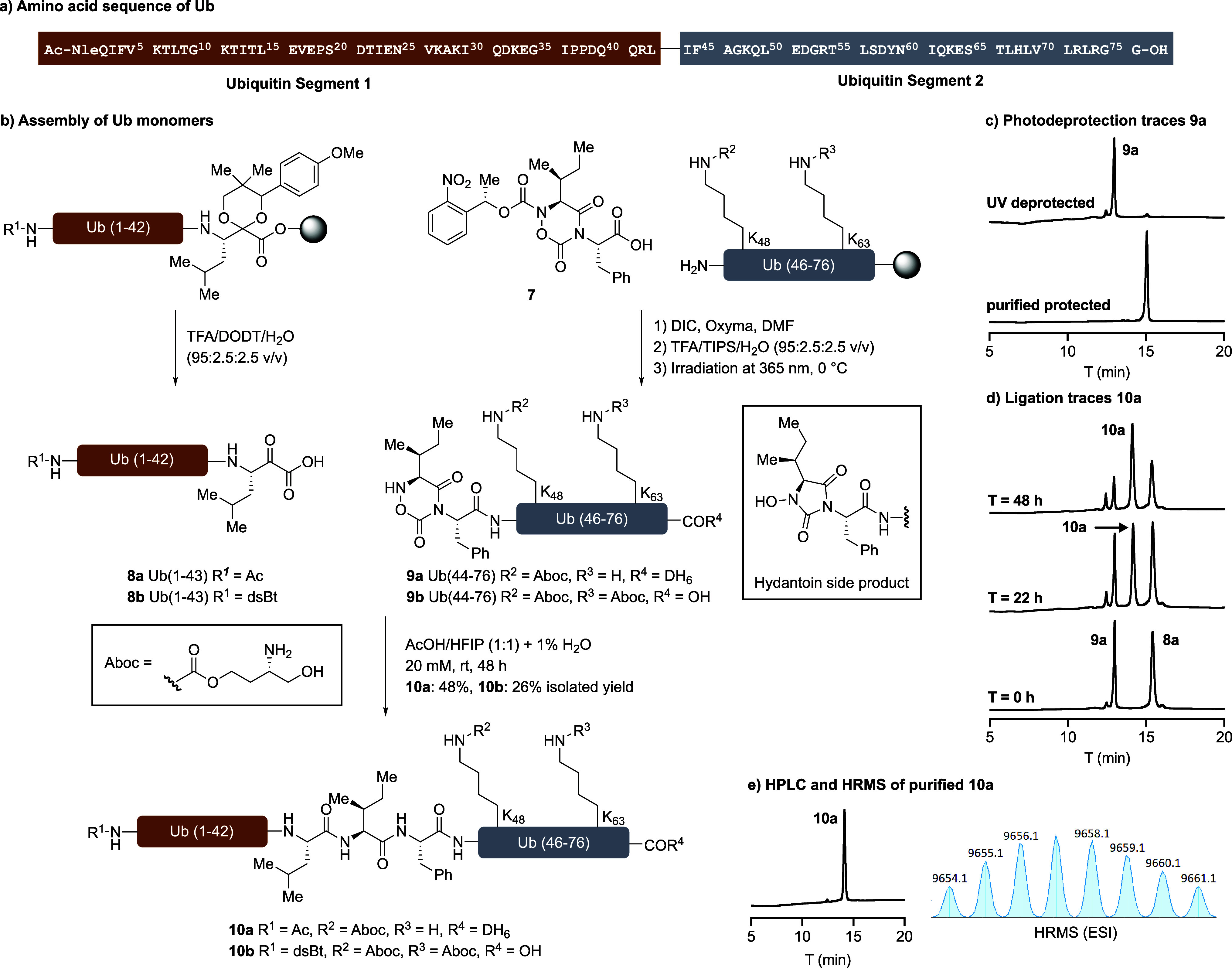
(a) Amino Acid Sequence of Ub; (b) Assembly of Ub Monomers; (c) HPLC Traces of Photodeprotection of Hydroxylamine Segment **9a**; (d) HPLC Traces of KAHA Ligation between **8a** and **9a**; (e) HPLC Trace and Mass Spectrum of Purified Ubiquitin **10a** ^a^

a HRMS (ESI): calculated for C_426_H_690_N_125_O_131_, 9658.9; measured 9657.1.

The successful synthesis of Ub using this approach addresses a considerable bottleneck in the scalable synthesis of Ub monomers, which we presently require for other ongoing work. To date, we have produced these Ub variants by linear solid phase peptide synthesis, an effective but material-limited approach that is prone to challenges in purification and reproducibility. By using this two-segment ligation approach, we have prepared numerous Ub monomers on a 100 mg (20 μmol) scale via a single KAHA ligation.

Encouraged by these results, we turned our attention to the contemporary challenge of therapeutic peptide synthesis using KAHA ligation. The demand for such targets and the inherent synthetic challenges are exemplified by tirzepatide (TZP), a 39-residue GLP-1 (glucagon-like peptide-1) receptor agonist incorporating four non-natural residues: a *C*-terminal serine amide, two Aib residues at positions 2 and 13, and a fatty acid side chain installed on Lys20.^38^ Of considerable interest for the sustainable production of TZP and other GLP-1 therapeutics, strategies combining SPPS and chemical ligation have gained significant attention.^39,40^ This consideration suggested TZP assembly from an *N*-terminal segment bearing the two Aib residues and a lysine α-ketoacid at its *C*-terminus, and a 23-residue peptide modified with an *N*-terminal Ile-Ala cyclic hydroxylamine dipeptide, a *C*-terminal serine amide, and the commercially available fatty acid side chain on Lys20.

We prepared the *C*-terminal segment by automated SPPS until Lys20. The orthogonal protecting groups of Alloc-Lys­(Fmoc)-OH incorporated at this position allow conjugation to the fatty acid containing moiety. After subsequent Alloc deprotection and coupling of a protected glutamine, the cyclic Ile-Ala hydroxylamine **11** was attached to the *N*-terminus using DIC/Oxyma (Scheme 3a). We observed that coupling efficiency was influenced in the presence of the fatty acid side chain, presumably due to increased steric hindrance. This impediment was circumvented by employing iterative coupling in conjunction with an elongated reaction time. The dipeptide hydroxylamine **11** was synthesized from Boc-Ile-Ala-OAllyl by the analogous route shown in Scheme 1b, suggesting that this approach will be suitable for the preparation of other dipeptide hydroxylamines. The photodeprotection proceeded efficiently to afford unmasked peptide **13** in a clean manner (Scheme 3b).

**Scheme 3:**
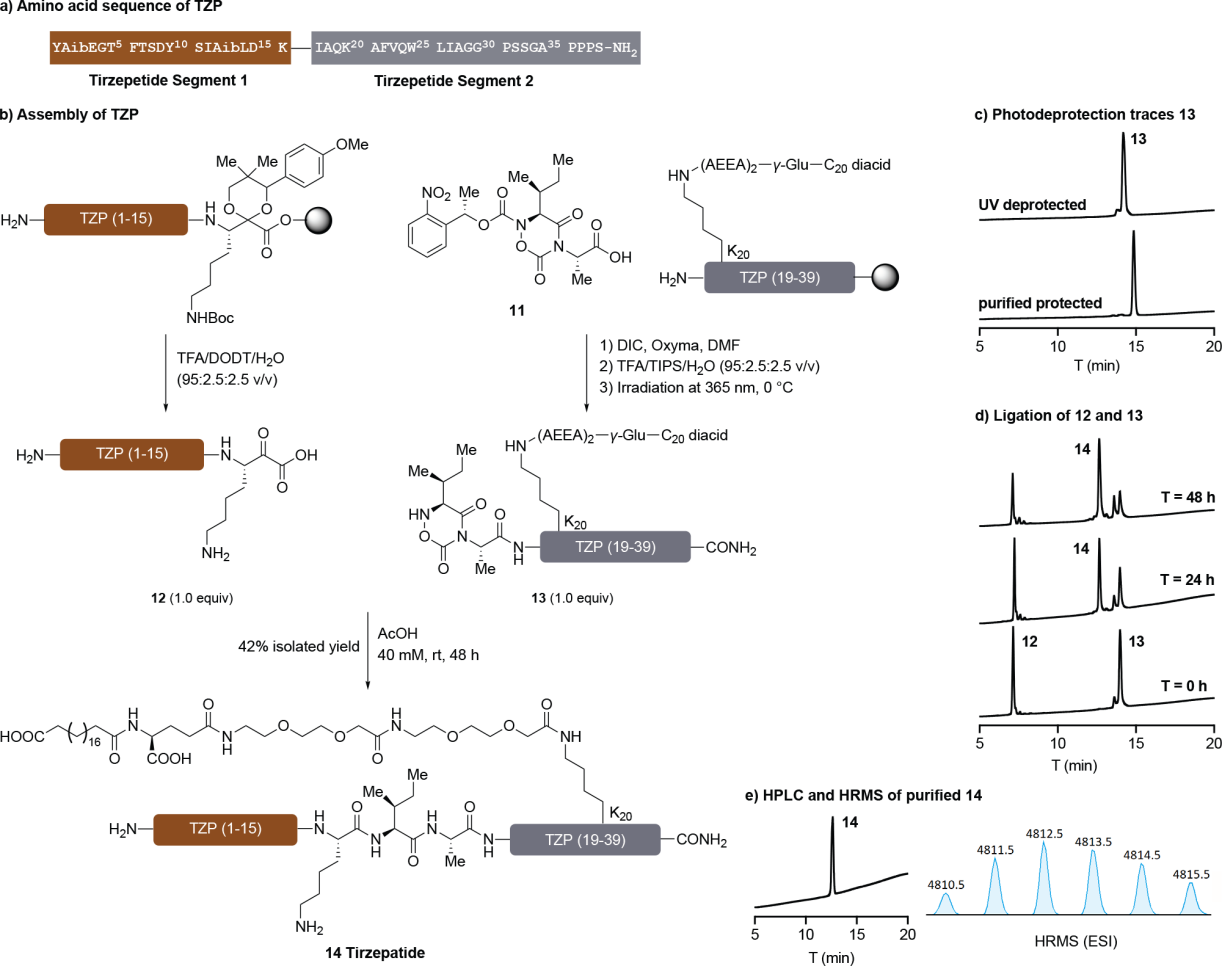
(a) Amino Acid Sequence of TZP; (b) Assembly of TZP; (c) HPLC Traces of Photodeprotection of Hydroxylamine Segment **13**; (d) HPLC Traces of KAHA Ligation between **12** and **13**; (e) HPLC Trace and Mass Spectrum of Purified TZP **14** ^a^

a HRMS (ESI): calculated for C_225_H_348_N_48_O_68_, 4813.5; measured 4812.5.

The Aib-containing *N*-terminal peptide was prepared on a polystyrene linker loaded with a protected lysine-derived α-ketoacid.^41^ Although we have previously synthesized and reported this α-ketoacid, it had never been tested on a peptide for KAHA ligation. Given that the unprotected amine could cyclize onto the ketone, as sometimes occurs with lysine-derived peptide thioesters,^42^ we were initially skeptical that the unprotected lysine α-ketoacid could serve as a ligation partner. Fortunately, the preparation of the 15-residue α-ketoacid peptide **12** proceeded smoothly and could be purified by preparative HPLC without any trace of cyclization or oligomerization.

With both reaction partners in hand, we examined the KAHA ligation between **12** and **13** to form TZP **14**. The ligation was conducted in pure AcOH, selected for the reasonable solubility of both segments and sustainability considerations. The formation of the hydantoin side product was observed again between peaks **13** and **14** on HPLC traces (Scheme 3d). Following incubation at ambient temperature for 48 h followed by HPLC purification afforded tirzepatide **14** in 42% isolated yield.

In conclusion, we have developed cyclic hydroxylamine building blocks suitable for KAHA ligations, resulting in canonical amino acids and amide bonds directly at the ligation sites, thereby overcoming limitations associated with (*S*)-5-oxaproline-based ligation. The established synthetic route, initiated from readily accessible dipeptides, potentially tolerates a wide range of amino acid combinations. The ability to conduct chemoselective peptide ligations at arbitrary and unexpected sites, in this case Leu–Ile and Lys–Ile, without further downstream processing has particular value in contexts such as peptide API production, where the increased synthetic overhead of accessing the building blocks may be justified. The peptide assembly—including hydroxylamine coupling, resin cleavage, and photodeprotection—proceeded smoothly, demonstrating the potential of this methodology for scale-up and broader applications. These hydroxylamines also enable KAHA ligation under sustainable conditions (AcOH, rt) that are also well-suited for hydrophobic or heat-sensitive peptides. These results highlight the versatility of the cyclic hydroxylamine-based KAHA ligation in chemical biology and peptide therapeutics, providing a robust and efficient strategy for the convergent assembly of fully native peptides and proteins.^43^

## Supplementary Material



## Notes

Experimental procedures; characterization data by NMR, IR, HPLC, and mass spectrometry (PDF)

## †

Department of Chemistry, Graduate School of Science, University of Osaka, Toyonaka, Osaka 560-0043, Japan
