# Supplementary material for: Cyclic Hydroxylamines for Native Residue-Forming Peptide Ligations: Synthesis of Ubiquitin and Tirzepatide
Source: J Am Chem Soc. 2025 Sep 11;147(38):34238–43. doi: 10.1021/jacs.5c11881 (PMC12464992; doi:10.1021/jacs.5c11881)
Supplement: Supplementary file 1 [file ja5c11881_si_001.pdf]

Supporting Information

Cyclic Hydroxylamines for Native Residue–Forming Peptide Ligations: Synthesis of Ubiquitin and Tirzepatide

Jiling Han, Kohtaro Hirao†, Toshiki Mikami, Nicolas Y. Nötel,
Leonardo L. Seidl and Jeffrey W. Bode*

Department of Chemistry and Applied Biosciences, ETH Zürich
Vladimir Prelog Weg 3, 8093 Zürich, Switzerland

E-mail: bode@org.chem.ethz.ch

Table of Contents

1. General Information & Methods...3

1.1. Reagents and solvents 3

1.2. Thin layer chromatography and flash chromatography 3

1.3. High-performance liquid chromatography...3

1.4. IR and NMR 3

1.5. LC-MS and HRMS 4

1.6. Solid phase peptide synthesis 4

1.7. Resin cleavage 4

2. Synthesis of Isoleucine-Phenylalanine Hydroxylamine Monomer 5

3. Synthesis of Isoleucine-Alanine Hydroxylamine Monomer..11

4. Mechanistic Studies 17

5. Synthesis of Ubiquitin 19

5.1. Ac-Ub(K48-Aboc)-DH_6 (10a)...19

5.1.1. Ac-Ub-(1-43)-Leu-α-ketoacid (8a) 19

5.1.2. Photoprotected-Ub(44-76)-(K48-Aboc)-DH_6 (S14) 21

5.1.3. Photodeprotection - Ub(44-76)-(K48-Aboc)-DH_6 (9a)..23

5.1.4. KAHA ligation - Ac-Ub(K48-Aboc)-DH_6 (10a) 24

5.2. Desthiobiotin-Ub(K48-Aboc, K63-Aboc)-OH (10b) 26

5.2.1. dsBt-Ub-(1-43)-Leu-α-ketoacid (8b) 26

5.2.2. Photoprotected-Ub(44-76)-(K48-Aboc, K63-Aboc)-OH (S15) 28

5.2.3. Photodeprotection - Ub(44-76)-(K48-Aboc, K63-Aboc)-OH (9b) 30

5.2.4. KAHA ligation - dsBt-Ub(K48-Aboc, K63-Aboc)-OH (10b) 31

6. Synthesis of Tirzepatide (14)..33

6.1. Tirzepatide-(1-16)-Lys-α-ketoacid (12) 33

6.2. Photoprotected-Tirzepatide-(17-39) (S16)..35

6.3. Photodeprotection - Tirzepatide-(17-39) (13) 37

6.4. KAHA ligation - Tirzepatide (14) 38

7. NMR Spectra 40

8. References 54

1. General Information & Methods

1.1. Reagents and solvents

Fmoc-amino acids with corresponding side-chain protecting groups, HCTU and HATU were purchased from Peptides International (Louisville, KY, USA) and ChemImpex (Wood Dale, IL, USA). Solvents for flash chromatography and SPPS were of technical grade. HPLC grade CH_3CN from Sigma-Aldrich was used for analytical and preparative HPLC purification. Other commercially available reagents and solvents were purchased from Sigma-Alrich (Buchs, Switzerland), Acros Organics (Geel, Belgium) and TCI Europe (Zwijndrecht, Belgium). All other commercially available reagents and solvents were used without further purification. H_2O used for reactions was purified using a Millipore system (MQ-H_2O).

1.2. Thin layer chromatography and flash chromatography

Reactions and fractions from flash chromatography were monitored by thin layer chromatography using precoated silica gel plates (Merck, silica 60 F254) and visualized by UV at 254 nm or staining with basic $KMnO_4$ aqueous solution. Flash chromatography was performed on Sigma Aldrich SiO_2 Type F60 (high-purity grade, 60 Å pore size, 230-400 mesh particle size) using a N_2 flow.

1.3. High-performance liquid chromatography

Analysis and purification by reversed phase high performance liquid chromatography (RP-HPLC) were conducted on Jasco analytical and preparative instruments equipped with dual pumps, mixer and in-line degasser, a variable wavelength UV detector (simultaneous monitoring of the eluent at 220 nm, 254 nm and 301 nm) and a Rheodyne injector fitted with a 1000 μl or 20 mL injection loop. If required, the columns were heated using a water bath. Gram-scale reversed phase purification was conducted on the Biotage Isolera™ System using a dry load vessel. The mobile phase for RP-HPLC were Milipore-H_2O containing 0.1% (v/v) TFA and HPLC grade CH_3CN containing 0.1% (v/v) TFA. The column, gradient and temperature will be stated at the individual experiments.

1.4. IR and NMR

IR spectra were recorded on a JASCO FT-IR-4100 spectrometer and reported as wavenumbers in cm-1. ^1H and ^{13}C NMR spectra were recorded on Bruker DRX400, Bruker AVIII400 and Bruker AVIII500 spectrometers. The chemical shifts of ^1H and ^{13}C NMR were referenced to the solvent peaks: δ=7.26 and δ=77.0 for $CDCl_3$ are given from tetramethylsilane (TMS) as an internal standard. Multiplicities abbreviations: s = singlet, d = doublet, t = triplet, q = quartet, m = multiplet, br = broad.

1.5. LC-MS and HRMS

LC-MS measurement of peptides was performed on an ultra-high performance liquid chromatography (Acquity) coupled to a QTOF-MS instrument (Bruker, Compact Q-TOF MS, or Waters, G2 XS Q-TOF) using InfinityLab Poroshell C18 column (120 å, 2.1 x 50 mm, 1.9 μm). High-resolution mass spectra were recorded by the Mass Service of the Laboratory of Organic Chemistry at ETH Zurich on a Bruker solariX - ESI-FTICR-MS, a Bruker maXis - ESI-Qq-TOF-MS, a Bruker solariX – MALDI-FTICR-MS, Bruker- Compact-20260, or a Bruker UltraFlex II – MALDI-TOF-MS.

1.6. Solid phase peptide synthesis

Peptides were synthesized on Symphony®X (GYROS PROTEIN Technology) parallel synthesizer using Fmoc SPPS chemistry. The following Fmoc amino acids with side-chain protection groups were used: Fmoc-Ala-OH, Fmoc-Arg(Pbf)-OH, Fmoc-Asn(Trt)-OH, Fmoc-Asp(OtBu)-OH, Fmoc-Gln(Trt)-OH, Fmoc-Glu(OtBu)-OH, FmocGly-OH, Fmoc-His(1-Trt)-OH, Fmoc-Ile-OH, Fmoc-Leu-OH, Fmoc-Lys(Boc)-OH, Fmoc-Phe-OH, Fmoc-Pro-OH, Fmoc-Ser(OtBu)-OH, Fmoc-Thr(OtBu)-OH, Fmoc-Trp(Boc)-OH, Fmoc-Tyr(OtBu)-OH, Fmoc-Val-OH, Fmoc-Nle-OH. SPPS was performed on 2-chlorotrityl chloride polystyrene resin or Rink-amide polystyrene resin. Manual loading of the first amino acid residue on the resin and subsequent Fmoc-SPPS, followed established standard protocols. The substitution capacity was determined by UV (λ = 304 nm) quantification of dibenzofulvene group released after treating with 2% DBU in DMF. Fmoc-deprotections were performed with 20 % piperidine in DMF (2 x 10 min). Standard couplings were performed with Fmoc amino acid (4.0 equiv to resin substitution), HCTU (3.9 equiv) and NMM (8.0 equiv) in DMF for 30 min and repeated once. After coupling, unreacted free amine was capped by treatment with 20 % acetic anhydride and NMM (1.5 equiv to acetic anhydride). Special building blocks and non-standard amino acids were coupled manually.

1.7. Resin cleavage

Peptide cleavage procedure from resin: To peptide segments synthesized on resin in the glass vial was added a solution of TFA/TIPS/H_2O (95:2.5:2.5 v/v/v, 15 mL/g resin) or TFA/DODT/H_2O (for α-ketoacid resin, 95:2.5:2.5 v/v/v, 15 mL/g resin). After being shaken for 2 h, the resin was filtered off. The filtrate was volatiled by N_2 gas. The residue was triturated with cold Et_2O/*n*-pentane (3:1), vortexed and centrifuged (4000 × rpm, 3 min). The supernatant was removed by decantation. This trituration/washing step was repeated twice. The residue was dissolved in a solution of CH_3CN/H_2O (1:1 v/v, + 0.1% TFA) for RP-HPLC purification.

2. Synthesis of Isoleucine-Phenylalanine Hydroxylamine Monomer

Allyl (*tert*-butoxycarbonyl)-*L*-phenylalaninate S2

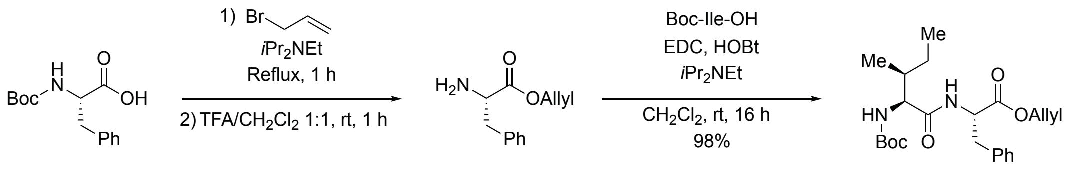

S1 **S2**

In a round bottom flask, Boc-Phe-OH (55.7 g, 210.0 mmol, 1.0 equiv) was dissolved in allyl bromide (350 mL). After addition of *N,N*-diisopropylethylamine (73.2 mL, 420.0 mmol, 2.0 equiv), the solution was heated to reflux (around 100 °C) and stirred for 1 h. The clean yellow solution became dark red and precipitate formed. The reaction mixture was cooled to rt and diluted with EtOAc (500 mL). After filtration the solution was washed with a 1 M HCl aqueous solution (500 mL x 2). The phases were separated, and the organic phase was washed with a saturated $NaHCO_3$ aqueous solution (500 mL), brine (500 mL), dried over Na_2SO_4, and concentrated under reduced pressure to afford an orange oil. The product was used for the next step without further purification.

In a round bottom flask, the obtained material was dissolved in a mixture of CH_2Cl_2 and TFA (1:1 v/v, 200 mL). The reaction mixture was stirred at rt for 1 h and concentrated under reduced pressure (co-evaporation with MeOH) to afford **S1** as a dark red oil. The material was used for the next reaction without further purification.

In a round bottom flask, the obtained material, Boc-Ile-OH (45.1 g, 195.2 mmol, 1.0 equiv) and 6-Chlor-1-hydroxy-benzotriazol (39.3 g, 204.9 mmol, 1.05 equiv) were dissolved in CH_2Cl_2 (650 mL, 0.3 M). To the solution, 1-ethyl-3-(3-dimethylaminopropyl)carbodiimide (33.1 g, 195.2 mmol, 1.0 equiv) and *N,N*-diisopropylethylamine (135.1 mL, 780.7 mmol, 4.0 equiv) were added. After being allowed to stir at rt for 16 h, the reaction mixture was quenched with a 1 M aqueous HCl aqueous solution (500 mL x 2) and the generated precipitate was filtered off. The phases were separated, and the aqueous phase was extracted with CH_2Cl_2 (300 mL x 2). The combined organic layers were washed with a saturated $NaHCO_3$ aqueous solution (500 mL), and brine (500 mL), dried over Na_2SO_4, and concentrated under reduced pressure. The residue was purified by flash column chromatography (hexane/EtOAc = 3:1 to 1:1, Rf = 0.4 in hexane/EtOAc = 3:2) to afford **S2** (80.2 g, 191.6 mmol, 98%) as a white solid.

^{1}H NMR (500 MHz, $CDCl_3$) δ [ppm] 7.31 – 7.11 (m, 5H), 6.42 (d, *J* = 7.8 Hz, 1H), 5.84 (ddt, *J* = 17.2, 10.4, 6.0 Hz, 1H), 5.29 (dq, *J* = 17.2, 1.5 Hz, 1H), 5.24 (dq, *J* = 10.4, 1.2 Hz, 1H), 5.14 – 5.01 (m, 1H), 4.89 (dt, *J* = 7.8, 6.0 Hz, 1H), 4.59 (dt, *J* = 6.0, 1.3 Hz, 2H), 4.02 – 3.89 (m, 1H), 3.25 – 3.04 (m, 2H), 1.82 (m, 1H), 1.44 (s, 9H), 1.43 (m, 1H), 1.12 – 1.02 (m, 1H), 0.91 – 0.84 (m, 6H).

^{13}C NMR (125 MHz, $CDCl_3$) δ [ppm] 171.19, 170.91, 155.64, 135.63, 131.35, 129.35, 128.62, 127.19, 119.17, 77.23, 66.11, 59.25, 53.11, 38.03, 37.24, 28.32, 24.66, 15.43, 11.43.

HRMS (ESI-Qq-TOF): m/z, $[M+Na]^+$ calculated for $C_{23}H_{34}N_2NaO_5$: 441.2360, found 441.2358.

IR (cm^{-1}, ATR): 3312, 2976, 1724, 1677, 1648, 1559, 1522, 1365, 1252, 1147.

Allyl (cyanomethyl)-*L*-alloisoleucyl-*L*-phenylalaninate S3[1]

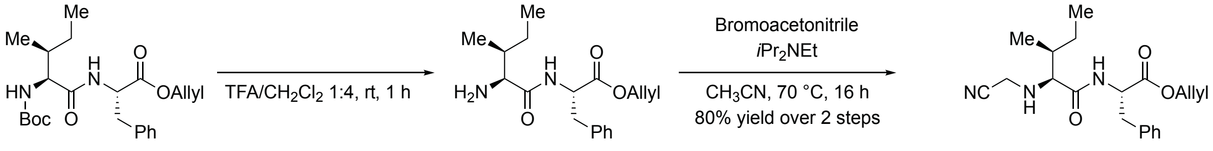

S2 **1** **S3**

In a round bottom flask, **S2** (5.5 g, 16.1 mmol, 1.0 equiv) was dissolved in a mixture of CH_2Cl_2 and TFA (4:1 v/v, 29 mL). The reaction mixture was stirred at rt for 1 h and concentrated under reduced pressure (co-evaporation with MeOH) to afford **1** as a yellow solid. The material was used for the next reaction without further purification.

In a round bottom flask, the obtained material was dissolved in CH_3CN (80 mL, 0.2 M). To this solution was added *N,N*-diisopropylethylamine (8.4 mL, 48.2 mmol, 3.0 equiv). After being allowed to stir for 5 min, the mixture was added bromoacetonitrile (2.24 mL, 32.1 mmol, 2.0 equiv) and stirred at 70 °C for 16 h. The reaction mixture was cooled to rt and concentrated under reduced pressure. The residue was redissolved in CH_2Cl_2 (100 mL) and washed with a saturated $NaHCO_3$ aqueous solution (100 mL). The phases were separated, and the aqueous phase was extracted with CH_2Cl_2 (50 mL x 2). The combined organic phases were washed with brine (100 mL), dried over Na_2SO_4, and concentrated under reduced pressure. The residue was purified by flash column chromatography (hexane/EtOAc = 7:3, Rf = 0.6 in hexane/EtOAc = 1:1) to afford **S3** (3.6 g, 16.1 mmol, 80%) as a yellow solid.

^{1}H NMR (500 MHz, $CDCl_3$) δ [ppm] 7.35-7.24 (m, 3H), 7.20-7.16 (m, 2H), 7.03 (d, *J* = 8.9 Hz, 1H), 5.91 (ddt, *J* = 17.1, 10.4, 5.9 Hz, 1H), 5.39-5.28 (m, 2H), 5.00 (ddd, *J* = 8.9, 8.1, 5.3 Hz, 1H), 4.65 (dq, *J* = 5.9, 1.5 Hz, 2H), 3.62 (d, *J* = 17.6 Hz, 1H), 3.34 (d, *J* = 17.6 Hz, 1H), 3.24 (dd, *J* = 14.1, 5.3 Hz, 1H), 3.06 (dd, *J* = 14.1, 8.1 Hz, 1H), 1.74-1.67 (m, 1H), 1.33 (dqd, *J* = 13.5, 7.5, 4.1 Hz, 1H), 1.11-1.02 (m, 1H), 0.88-0.84 (m, 6H).

^{13}C NMR (125 MHz, $CDCl_3$) δ [ppm] 171.46, 171.42, 135.86, 131.36, 129.13, 128.69, 127.21, 119.34, 117.37, 66.47, 66.23, 52.40, 37.98, 37.90, 36.15, 24.93, 15.51, 11.48.

HRMS (ESI-Qq-TOF): m/z, $[M+H]^+$ calculated for $C_{20}H_{28}N_3O_3$: 358.2125, found 358.2118.

IR (cm^{-1}, ATR): 3356, 2965, 2875, 2238, 1742, 1662, 1559, 1453, 1272, 958.

Allyl hydroxy-*L*-alloisoleucyl-*L*-phenylalaninate 2[1]

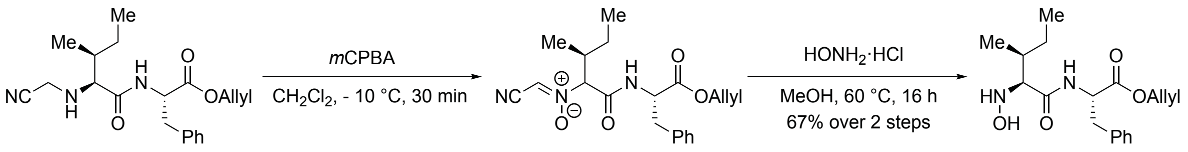

S3 **S4** **2**

In a round bottom flask, **S3** (8.00 g, 22.38 mmol, 1.0 equiv) was dissolved in CH_2Cl_2 (110 mL, 0.2 M). To this solution was added *m*-CPBA (11.03 g, 49.24 mmol, 2.2 equiv) over 30 min at -10 °C (Cooling bath: NaCl/ice = 1:1). The reaction mixture was quenched with a $Na_2S_2O_3$ (12.22 g, 49.24 mmol, 2.2 equiv) in H_2O (80 mL) and a saturated $NaHCO_3$ aqueous solution (280 mL). After dilution with CH_2Cl_2, the solution was stirred for 20 min. The phases were separated, and the aqueous phase was extracted with CH_2Cl_2 (200 mL x 2). The combined organic phases were washed with brine (300 mL), dried over Na_2SO_4, and concentrated under reduced pressure to afford **S4** as a white solid. The material was used for the next reaction without further purification.

In a round bottom flask, the obtained material was dissolved in MeOH (21 mL, 0.25 M). To the solution was added NH_2OH•HCl (1.95 g, 28.00 mmol, 5.0 equiv). After being allowed to stir at 60 °C for 16 h, the reaction mixture was cooled to rt and concentrated under reduced pressure (water bath temperature lower than 30 °C). The residue was suspended in CH_2Cl_2 (40 mL), filtered through Celite and washed with CH_2Cl_2 (30 mL). The solution was washed with a saturated $NaHCO_3$ aqueous solution (70 mL). The phases were separated, and the aqueous phase was extracted with CH_2Cl_2 (40 mL x 2). The combined organic phases were washed with brine (300 mL), dried over Na_2SO_4 and concentrated under reduced pressure (water bath temperature lower than 30 °C). The residue was purified by flash column chromatography (hexane/EtOAc = 1:1, Rf = 0.35 in hexane/EtOAc = 1:1) to afford **2** (1.25 g, 3.73 mmol, 67% in two steps) as a white solid.

^{1}H NMR (400 MHz, $CDCl_3$) δ [ppm] 7.34 – 7.15 (m, 5H), 6.91 (d, *J* = 8.9 Hz, 1H), 5.90 (ddt, *J* = 17.2, 10.4, 6.0 Hz, 1H), 5.39 – 5.27 (m, 2H), 5.05 (ddd, *J* = 8.9, 7.1, 5.5 Hz, 1H), 4.64 (ddt, *J* = 6.0, 2.5, 1.3 Hz, 2H), 3.40 (d, *J* = 6.1 Hz, 1H), 3.32 – 3.06 (m, 2H), 1.62 (m, 1H), 1.51 – 1.37 (m, 1H), 1.11 (m, 1H), 0.87 (t, *J* = 7.4 Hz, 3H), 0.83 (d, *J* = 6.9 Hz, 3H).

^{13}C NMR (100 MHz, $CDCl_3$) δ [ppm] 172.18, 172.11, 135.87, 131.32, 129.29, 128.58, 127.14, 119.32, 71.41, 66.32, 52.50, 37.99, 35.36, 25.82, 15.62, 11.35.

HRMS (ESI-Qq-TOF): m/z, $[M+H]^+$ calculated for for $C_{18}H_{27}N_2O_4$: 335.1965, found 335.1964.

IR (cm^{-1}, ATR): 3349, 2950, 2873, 1727, 1654, 1533, 1442, 1254, 1151, 936.

Allyl *N*-hydroxy-*N*-(((*S*)-1-(2-nitrophenyl)ethoxy)carbonyl)-*L*-alloisoleucyl-*L*-phenylalaninate (4)

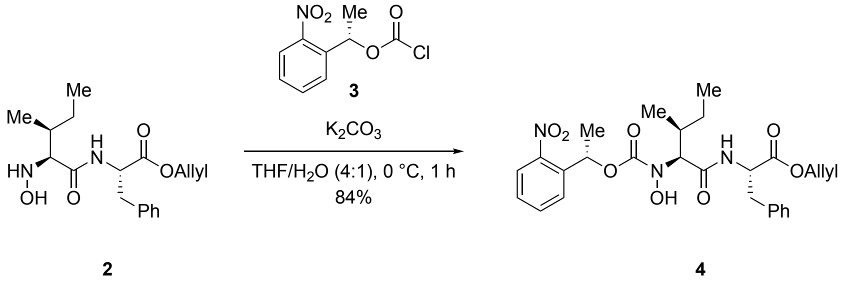

In a round bottom flask, **2** (4.0 g, 12.0 mmol, 1.0 equiv) was dissolved in a mixture of THF and H_2O (4:1 v/v, 10 mL). To the solution, (*S*)-1-(2-nitrophenyl)ethyl chloroformate **3**[2] (3.31 g, 14.5 mmol, 1.2 equiv) and K_2CO_3 (100 mg, 7.2 mmol, 0.60 equiv) were added at 0 °C. After being allowed to stir for 1 h, the reaction mixture was washed with H_2O. The aqueous phase was extracted with CH_2Cl_2. The organic layer was dried over Na_2SO_4. After concentration under reduced pressure, the residue was purified by flash column chromatography (hexane/EtOAc = 9:1 to 6:1) to afford **4** (5.26 g, 10 mmol, 84%) as a light yellow solid.

^{1}H NMR (400 MHz, $CDCl_3$) δ [ppm] 7.96 (dd, *J* = 8.2, 1.2 Hz, 1H), 7.64 (m, 2H), 7.46 – 7.37 (m, 1H), 7.31 – 7.21 (m, 3H), 7.11 (dd, *J* = 7.7, 1.7 Hz, 2H), 6.75 (s, 1H), 6.31 (q, *J* = 6.5 Hz, 1H), 5.79 (ddt, *J* = 16.5, 11.0, 5.8 Hz, 1H), 5.31 – 5.17 (m, 2H), 4.86 (td, *J* = 7.6, 5.5 Hz, 1H), 4.53 (d, *J* = 5.8 Hz, 2H), 4.28 (d, *J* = 10.0 Hz, 1H), 3.21 – 2.95 (m, 2H), 2.08 (m, 1H), 1.68 (d, *J* = 6.5 Hz, 3H), 1.50 (m, 1H), 1.13 (m, 1H), 0.84 (m, 6H).

^{13}C NMR (100 MHz, $CDCl_3$) δ [ppm] 171.12, 170.54, 156.43, 147.35, 138.09, 135.57, 133.95, 131.30, 129.24, 128.61, 128.32, 127.31, 127.21, 124.38, 119.02, 70.76, 66.40, 66.09, 53.14, 37.95, 33.55, 25.41, 22.25, 15.06, 10.44.

HRMS (ESI-Qq-TOF): m/z, $[M+Na]^+$ calculated for $C_{27}H_{33}N_3NaO_8$: 550.2160, found 550.2151.

IR (cm^{-1}, ATR): 3316, 2955, 2873, 1735, 1641, 1432, 1190, 1095, 961.

Allyl N-(((4-nitrophenoxy)carbonyl)oxy)-N-(((S)-1-(2-nitrophenyl)ethoxy)carbonyl)-L-alloisoleucyl-L-phenylalaninate (5)

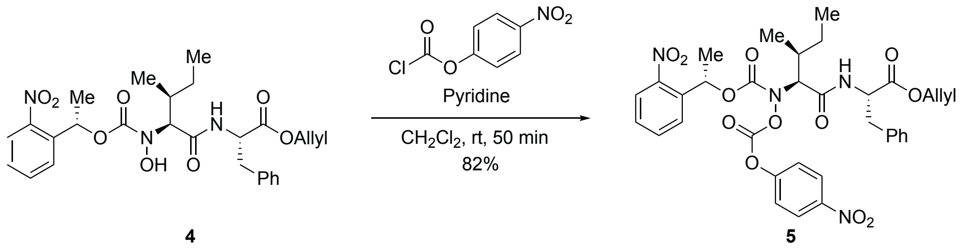

In a round bottom flask, **4** (2.5 g, 4.74 mmol) was dissolved in CH_2Cl_2 (7.5 mL). To the solution were added 4-nitrophenyl chloroformate (980 mg, 4.86 mmol, 1.03 equiv) and pyridine (420 μL, 5.21 mmol, 1.1 equiv). After being allowed to stir for 50 min at rt, the reaction mixture was quenched with a 1 M HCl aqueous solution. The aqueous phase was extracted with CH_2Cl_2. The organic layer was dried over Na_2SO_4. After concentration under reduced pressure, the residue was purified by reverse phase HPLC using the Biotage Sfär C18 column (30 *μ*m, 100 Å pore size, 60 g) with a gradient of 5–100% CH_3CN in H_2O + 0.1% TFA over 10 column volumes at rt, with a flow rate of 50 mL/min. The pure product fractions were combined and lyophilized to obtain **5** (2.68 g, 3.87 mmol, 82%) as a yellow oil.

^{1}H NMR (500 MHz, $CDCl_3$) δ [ppm] 8.32 (d, *J* = 8.8 Hz, 2H), 7.99 (d, *J* = 8.2 Hz, 1H), 7.66 (m, 2H), 7.53 – 7.41 (m, 3H), 7.24 (s, 3H), 7.17 – 7.11 (m, 2H), 6.63 (s, 1H), 6.38 (s, 1H), 5.86 (ddt, *J* = 16.6, 10.4, 5.9 Hz, 1H), 5.36 – 5.22 (m, 2H), 4.91 (s, 1H), 4.60 (d, *J* = 5.9, 2H), 4.39 (s, 1H), 3.12 (s, 2H), 2.34 (s, 1H), 1.71 (s, 3H), 1f.44 (s, 1H), 1.09 (s, 1H), 1.00 – 0.78 (m, 6H).

^{13}C NMR (125 MHz, $CDCl_3$) δ [ppm] 170.68, 167.57, 155.14, 153.75, 151.80, 147.68, 145.87, 136.86, 135.51, 133.72, 131.34, 129.28, 128.82, 128.58, 127.22, 127.03, 125.52, 124.67, 121.59, 119.14, 115.62, 72.17, 66.16, 53.21, 37.61, 33.41, 24.90, 22.05, 15.17, 10.89.

HRMS (ESI-Qq-TOF): m/z, $[M+Na]^+$ calculated for $C_{34}H_{36}N_4NaO_{12}$: 715.2222, found 715.2206.

IR (cm^{-1}, ATR): 3369, 3084, 2964, 2855, 1811, 1739, 1678, 1524, 1346, 1288, 1190, 992.

(S)-1-(2-nitrophenyl)ethyl (S)-5-((S)-1-(allyloxy)-1-oxo-3-phenylpropan-2-yl)-3-((R)-sec-butyl)-4,6-dioxo-1,2,5-oxadiazinane-2-carboxylate (6)

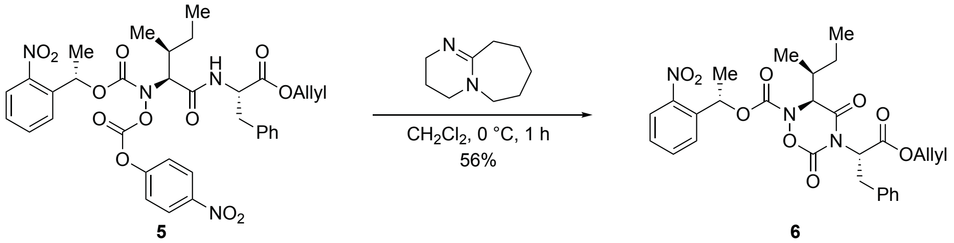

In a round bottom flask, **5** (128 mg, 185 μmol, 1.0 equiv) was dissolved in CH_2Cl_2 (3.7 mL). To the solution was added DBU (13.8 μL, 92.54 μmol, 0.5 equiv) at 0 °C. After being allowed to stir for 1 h, the reaction mixture was quenched with a 1 M HCl aqueous solution. The aqueous phase was extracted with CH_2Cl_2. The organic layer was dried over Na_2SO_4. After concentration under reduced pressure, the residue was purified by flash column chromatography (hexane/EtOAc = 95:5 to 90:10) to afford **6** (57 mg, 103 μmol, 56%) as a yellow oil.

^{1}H NMR (500 MHz, $CDCl_3$) δ [ppm] 7.99 (dd, *J* = 8.2, 1.3 Hz, 1H), 7.66 (td, *J* = 7.6, 1.3 Hz, 1H), 7.61 (dd, *J* = 8.2, 1.5 Hz, 1H), 7.49-7.44 (m, 1H), 7.30 (m, 3H), 7.17-7.12 (m, 2H), 6.14 (q, *J* = 6.4 Hz,

1H), 5.92 (ddt, J = 17.3, 10.4, 6.0 Hz, 1H), 5.47 (dd, J = 11.6, 5.5 Hz, 1H), 5.36 (dq, J = 17.3, 1.5 Hz, 1H), 5.30 (dq, J = 10.4, 1.2 Hz, 1H), 4.69 (dt, J = 6.0, 1.4 Hz, 2H), 4.48 (d, J = 9.0 Hz, 1H), 3.63-3.44 (m, 2H), 1.75-1.69 (m, 1H), 1.58 (d, J = 6.4 Hz, 3H), 1.47 (m, 1H), 1.17 (m, 1H), 0.82 (t, J = 7.4 Hz, 3H), 0.74 (d, J = 6.8 Hz, 3H).

^{13}C NMR (125 MHz, $CDCl_3$) δ [ppm] 167.62, 166.56, 154.04, 151.03, 147.49, 137.13, 135.90, 133.96, 131.24, 129.16, 128.81, 128.76, 127.39, 126.86, 124.58, 119.22, 72.14, 66.75, 63.57, 56.18, 34.50, 34.32, 25.28, 21.93, 14.81, 10.46.

HRMS (ESI-Qq-TOF): m/z, $[M+Na]^+$ calculated for $C_{28}H_{31}N_3NaO_9$: 576.1953, found 576.1944.

IR (cm^{-1}, ATR): 3085, 2967, 2878, 1784, 1723, 1612, 1526, 1456, 1339, 1249, 935.

(*S*)-2-((*S*)-3-((*R*)-*sec*-butyl)-2-(((*S*)-1-(2-nitrophenyl)ethoxy)carbonyl)-4,6-dioxo-1,2,5-oxadiazinan-5-yl)-3-phenylpropanoic acid (7)

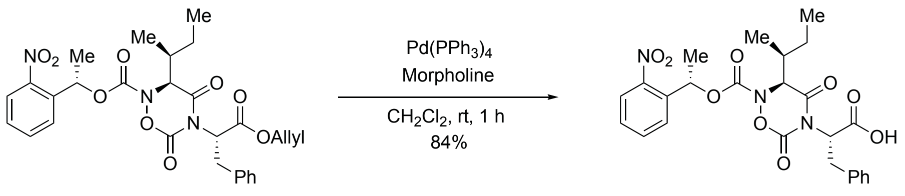

6 **7**

In a round bottom flask, **6** (210 mg, 0.379 mmol) was dissolved in CH_2Cl_2 (3.8 mL). To the solution were added tetrakis(triphenylphosphine)palladium (11.5 mg, 9.95 μmol, 0.026 equiv) and morpholine (34.0 μL, 0.394 mmol, 1.04 equiv). After being allowed to stir for 60 min at rt, the reaction mixture was quenched with a 10% citric acid aqueous solution. The aqueous phase was extracted with CH_2Cl_2. The organic layer was dried over Na_2SO_4. After concentration under reduced pressure, the residue was purified by reverse phase HPLC using the Shiseido Capcell Pak MGII C18 column (5 µm, 100 Å pore size, 250 × 20 mm) with a gradient of 60–85–95% CH_3CN in H_2O + 0.1% TFA over 30 min at rt, with a flow rate of 40 mL/min. The pure product fractions were combined and lyophilized to obtain **7** (164 mg, 0.319 mmol, 84 %) as a light yellow solid.

^{1}H NMR (500 MHz, $CDCl_3$) δ 9.72 (br, 1H), 8.00 (dd, J = 8.3, 1.3 Hz, 1H), 7.68 (td, J = 7.6, 1.3 Hz, 1H), 7.63 (dd, J = 7.9, 1.6 Hz, 1H), 7.47 (ddd, J = 8.3, 7.2, 1.6 Hz, 1H), 7.34-7.29 (m, 3H), 7.16 (m, 2H), 6.17 (q, J = 6.4 Hz, 1H), 5.56 (dd, J = 11.2, 5.7 Hz, 1H), 4.48 (d, J = 9.2 Hz, 1H), 3.64-3.44 (m, 2H), 1.68 (m, 1H), 1.58 (d, J = 6.4 Hz, 3H), 1.47 (m, 1H), 1.16 (m, 1H), 0.82 (t, J = 7.4 Hz, 3H), 0.72 (d, J = 6.8 Hz, 3H).

^{13}C NMR (125 MHz, $CDCl_3$) δ 173.93, 166.43, 154.16, 150.93, 147.46, 136.95, 135.49, 134.02, 129.21, 128.85, 128.82, 127.51, 126.95, 124.58, 72.24, 63.52, 55.53, 34.38, 34.12, 25.34, 21.93, 14.76, 10.35.

HRMS (ESI-Qq-TOF): m/z, $[M+Na]^+$ calculated for $C_{25}H_{27}N_3NaO_9$: 536.1640, found 536.1635.

IR (cm^{-1}, ATR): 3255, 2965, 2877, 1781, 1730, 1698, 1524, 1455, 1224, 930.

3. Synthesis of Isoleucine-Alanine Hydroxylamine Monomer

Allyl (*tert*-butoxycarbonyl)-*L*-alloisoleucyl-*L*-alaninate (S6)

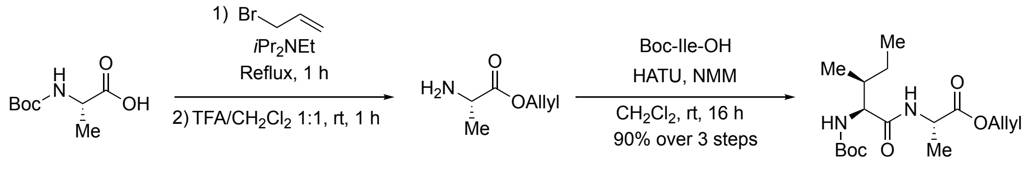

S5 **S6**

In a round bottom flask, Boc-Ala-OH (30.0 g, 158.6 mmol, 1.0 equiv) was dissolved in allyl bromide (300 mL). After addition of *N,N*-diisopropylethylamine (55.2 mL, 317.1 mmol, 2.0 equiv), the solution was heated to reflux (around 100 °C) and stirred for 1 h. The clean yellow solution became dark red and precipitate formed. The reaction mixture was cooled to rt and diluted with EtOAc (500 mL). After filtration the solution was washed with a 1 M HCl aqueous solution (500 mL x 2). The phases were separated, and the organic phase was washed with a saturated $NaHCO_3$ aqueous solution (500 mL), brine (500 mL), dried over Na_2SO_4, and concentrated under reduced pressure to afford an orange oil. The product was used for the next step without further purification.

In a round bottom flask, the obtained material was dissolved in a mixture of CH_2Cl_2 and TFA (1:1 v/v, 160 mL). The reaction mixture was stirred at rt for 1 h and concentrated under reduced pressure (co-evaporation with MeOH) to afford **S5** as a brown oil. The material was used for the next reaction without further purification.

In a round bottom flask, the obtained material, Boc-Ile-OH (40.3 g, 174.4 mmol, 1.1 equiv) and HATU (60.3 g, 158.6 mmol, 1.0 equiv) were dissolved in CH_2Cl_2 (700 mL, 0.2 M). To the solution, *N*-Methylmorpholine (69.8 mL, 634.2 mmol, 4.0 equiv) were added. After being allowed to stir at rt for 16 h, the reaction mixture was quenched with a 1 M aqueous HCl aqueous solution (500 mL x 2) and the generated precipitate was filtered off. The phases were separated, and the aqueous phase was extracted with CH_2Cl_2 (300 mL x 2). The combined organic layers were washed with a saturated $NaHCO_3$ aqueous solution (500 mL), and brine (500 mL), dried over Na_2SO_4, and concentrated under reduced pressure. The residue was purified by flash column chromatography (hexane/EtOAc = 90:10 to 75:25, Rf = 0.55 in hexane/EtOAc = 1:1) to afford **S6** (49.1 g, 143.4 mmol, 90%) as a white solid.

^{1}H NMR (400 MHz, $CDCl_3$) δ [ppm] 6.55 (s, 1H), 5.91 (ddtd, *J* = 17.2, 10.4, 5.8, 0.9 Hz, 1H), 5.34 (dp, *J* = 17.2, 1.4 Hz, 1H), 5.27 (dp, *J* = 10.4, 1.4 Hz, 1H), 5.12 (s, 1H), 4.71 – 4.57 (m, 3H), 3.98 (t,

J = 7.8 Hz, 1H), 1.88 (s, 1H), 1.58 – 1.48 (m, 1H), 1.49 – 1.40 (m, 12H), 1.16 (ddq, J = 14.0, 9.3, 8.1 Hz, 1H), 0.95 (d, J = 6.9 Hz, 3H), 0.91 (t, J = 8.1 Hz, 3H).

^{13}C NMR (101 MHz, $CDCl_3$) δ [ppm] 172.34, 171.17, 155.76, 131.52, 118.80, 79.87, 65.97, 59.12, 48.06, 37.39, 28.31, 24.73, 18.32, 15.47, 11.42.

HRMS (ESI-Qq-TOF): m/z, $[M+Na]^+$ calculated for $C_{17}H_{30}N_2NaO_5$: 365.2047, found 365.2042.

IR (cm^{-1}, ATR): 3346, 3273, 2963, 2879, 1742, 1678, 1649, 1522, 1390, 1153.

Allyl (cyanomethyl)-*L*-alloisoleucyl-*L*-alaninate (S8)[1]

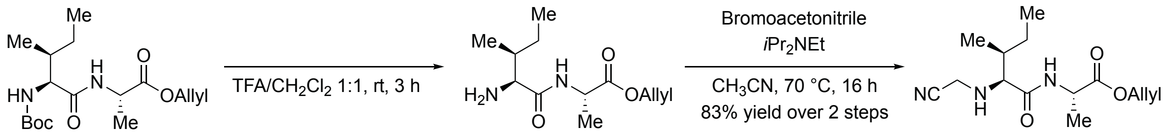

S6 **S7** **S8**

In a round bottom flask, **S6** (44.1 g, 128.8 mmol, 1.0 equiv) was dissolved in a mixture of CH_2Cl_2 and TFA (1:1 v/v, 128 mL). The reaction mixture was stirred at rt for 3 h and concentrated under reduced pressure (co-evaporation with MeOH) to afford **S7** as a brown oil. The material was used for the next reaction without further purification.

In a round bottom flask, the obtained material was dissolved in CH_3CN (640 mL, 0.2 M). To this solution was added *N,N*-diisopropylethylamine (67.3 mL, 386.3 mmol, 3.0 equiv). After being allowed to stir for 5 min, the mixture was added bromoacetonitrile (18.0 mL, 257.6 mmol, 2.0 equiv) and stirred at 70 °C for 16 h. The reaction mixture was cooled to rt and concentrated under reduced pressure. The residue was redissolved in CH_2Cl_2 (500 mL) and washed with a saturated $NaHCO_3$ aqueous solution (500 mL). The phases were separated, and the aqueous phase was extracted with CH_2Cl_2 (300 mL x 2). The combined organic phases were washed with brine (500 mL), dried over Na_2SO_4, and concentrated under reduced pressure. The residue was purified by flash column chromatography (hexane/EtOAc = 90:10 to 60:40, Rf = 0.25 in hexane/EtOAc = 2:1) to afford **S8** (30.0 g, 106.6 mmol, 83%) as a white solid.

^{1}H NMR (500 MHz, $CDCl_3$) δ 7.22 (d, J = 8.3 Hz, 1H), 5.90 (ddtd, J = 17.2, 10.4, 5.8, 1.2 Hz, 1H), 5.41 – 5.21 (m, 2H), 4.71 – 4.57 (m, 3H), 3.74 (dd, J = 17.5, 1.1 Hz, 1H), 3.52 (dd, J = 17.5, 1.1 Hz, 1H), 3.21 (dt, J = 5.0, 1.1 Hz, 1H), 1.79 (m, 1H), 1.61 – 1.47 (m, 1H), 1.43 (dd, J = 8.1, 1.0 Hz, 3H), 1.29 – 1.11 (m, 1H), 0.98 (dd, J = 7.0, 1.1 Hz, 3H), 0.91 (td, J = 7.3, 1.1 Hz, 3H).

^{13}C NMR (125 MHz, $CDCl_3$) δ 172.65, 171.33, 131.47, 118.91, 117.47, 66.38, 66.02, 47.55, 38.09, 36.34, 25.12, 18.31, 15.64, 11.55.

HRMS (ESI-Qq-TOF): m/z, $[M+Na]^+$ calculated for $C_{14}H_{23}N_3NaO_3$: 304.1632, found 304.1624.

IR (cm^{-1}, ATR): 3346, 3272, 2962, 2879, 2238, 1742, 1652, 1527, 1324, 1211,1151, 1105.

Allyl hydroxy-*L*-alloisoleucyl-*L*-alaninate (S10)[1]

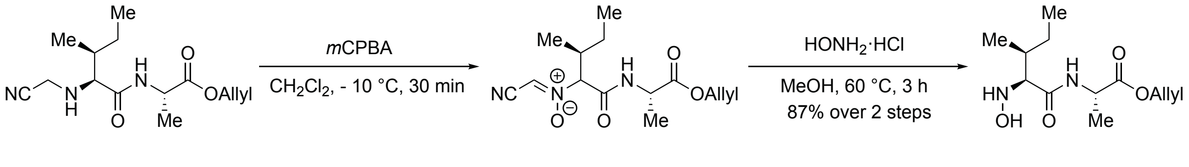

S8 **S9** **S10**

In a round bottom flask, **S8** (42.0 g, 149.3 mmol, 1.0 equiv) was dissolved in CH_2Cl_2 (750 mL, 0.2 M). To this solution was added *m*-CPBA (77 %, 66.9 g, 298.6 mmol, 2.0 equiv) over 30 min at -10 °C (Cooling bath: NaCl/Ice = 1:1). The reaction mixture was quenched with a saturated $Na_2S_2O_3$ aqueous solution (300 mL) and a saturated $NaHCO_3$ aqueous solution (500 mL). After dilution with CH_2Cl_2, the solution was stirred for 20 min. The phases were separated, and the aqueous phase was extracted with CH_2Cl_2 (400 mL x 2). The combined organic phases were washed with brine (500 mL), dried over Na_2SO_4, and concentrated under reduced pressure to afford **S9** as a white solid. The material was used for the next reaction without further purification.

In a round bottom flask, the obtained material was dissolved in MeOH (370 mL, 0.4 M). To the solution was added NH_2OH•HCl (41.5 g, 597.1 mmol, 4.0 equiv). After being allowed to stir at 60 °C for 3 h, the reaction mixture was cooled to rt and concentrated under reduced pressure. The residue was suspended in CH_2Cl_2 (400 mL), filtered through Celite and washed with CH_2Cl_2 (150 mL). The solution was washed with a saturated $NaHCO_3$ aqueous solution (500 mL). The phases were separated, and the aqueous phase was extracted with CH_2Cl_2 (300 mL x 2). The combined organic phases were washed with brine (500 mL), dried over Na_2SO_4 and concentrated under reduced pressure. The residue was purified by flash column chromatography (hexane/EtOAc = 90:10 to 50:50, Rf = 0.3 in hexane/EtOAc = 1:1) to afford **S10** (33.7 g, 149.3 mmol, 87% in two steps) as a brown oil.

^{1}H NMR (500 MHz, $CDCl_3$) δ 7.10 (d, *J* = 8.0 Hz, 1H), 5.92 – 5.83 (m, 1H), 5.30 (m, 1H), 5.25 – 5.17 (m, 1H), 4.73 – 4.65 (m, 1H), 4.65 – 4.55 (m, 2H), 3.38 (dt, *J* = 6.3, 1.4 Hz, 1H), 1.68 (m, 1H), 1.60 – 1.48 (m, 1H), 1.42 (d, *J* = 7.2, 3H), 1.17 (m, 1H), 0.91 (dd, *J* = 6.9, 1.4 Hz, 3H), 0.87 (t, *J* = 7.5 Hz, 3H).

^{13}C NMR (125 MHz, $CDCl_3$) δ 73.19, 172.32, 131.48, 118.76, 71.07, 66.02, 47.65, 35.46, 25.83, 18.28, 15.66, 11.38.

HRMS (ESI-Qq-TOF): m/z, $[M+H]^+$ calculated for $C_{12}H_{23}N_2O_4$: 259.1652, found 259.1647.

IR (cm^{-1}, ATR): 3203, 3080, 2965, 2877, 2361, 1739, 1456, 1321, 1201, 1156, 917.

Allyl *N*-hydroxy-*N*-(((*S*)-1-(2-nitrophenyl)ethoxy)carbonyl)-*L*-alloisoleucyl-*L*-alaninate (S11)[2]

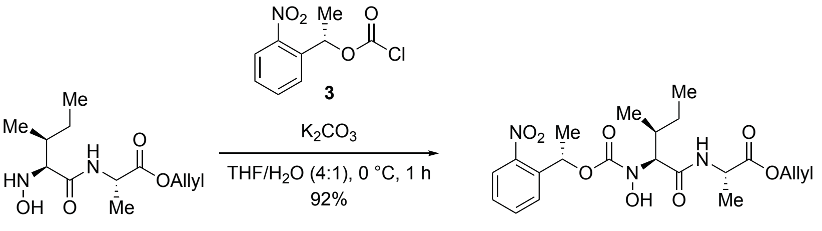

S10 **S11**

In a round bottom flask, **S10** (4.0 g, 15.5 mmol, 1.0 equiv) was dissolved in a mixture of THF and H_2O (4:1 v/v, 50 mL). To the solution, (*S*)-1-(2-nitrophenyl)ethyl chloroformate **3**[2] (4.3 g, 18.6 mmol, 1.2 equiv) and K_2CO_3 (1.3 g, 9.3 mmol, 0.60 equiv) were added at 0 °C. After being allowed to stir for 1 h, the reaction mixture was washed with H_2O. The aqueous phase was extracted with CH_2Cl_2. The organic layer was dried over Na_2SO_4. After concentration under reduced pressure, the residue was purified by flash column chromatography (CH_2Cl_2/MeOH = 100:0 to 98:2, Rf = 0.39 in hexane/EtOAc = 1:1) to afford **S11** (6.4 g, 14.3 mmol, 92%) as a transparent oil.

^{1}H NMR (400 MHz, $CDCl_3$) δ 7.96 (dd, *J* = 8.2, 1.3 Hz, 1H), 7.73 – 7.67 (m, 1H), 7.64 (td, *J* = 7.6, 1.3 Hz, 1H), 7.43 (ddd, *J* = 8.5, 7.2, 1.6 Hz, 1H), 6.76 (s, 1H), 6.32 (q, *J* = 6.4 Hz, 1H), 5.89 (ddt, *J* = 16.5, 11.0, 5.7 Hz, 1H), 5.39 – 5.22 (m, 2H), 4.67 – 4.51 (m, 3H), 4.30 (d, *J* = 9.9 Hz, 1H), 2.18 (m, 1H), 1.70 (d, *J* = 6.4 Hz, 3H), 1.55 (m, 1H), 1.42 (d, *J* = 7.2 Hz, 3H), 1.23 – 1.09 (m, 1H), 0.97 (d, *J* = 7.3 Hz, 3H), 0.85 (t, *J* = 7.4 Hz, 3H).

^{13}C NMR (100 MHz, $CDCl_3$) δ 171.92, 156.17, 147.46, 138.03, 133.82, 131.41, 128.35, 127.33, 124.43, 118.93, 73.19, 70.71, 66.10, 48.14, 35.95, 33.66, 25.50, 22.25, 18.01, 15.17, 10.48.

HRMS (ESI-Qq-TOF): m/z, $[M+Na]^+$ calculated for $C_{21}H_{29}N_3NaO_8$: 474.1847, found 474.1839.

IR (cm^{-1}, ATR): 3299, 3085, 2966, 2878, 2360, 1739, 1652, 1524, 1340, 1198, 1148, 856.

Allyl *N*-(((4-nitrophenoxy)carbonyl)oxy)-*N*-(((*S*)-1-(2-nitrophenyl)ethoxy)carbonyl)-*L*-alloisoleucyl-*L*-alaninate (S12)

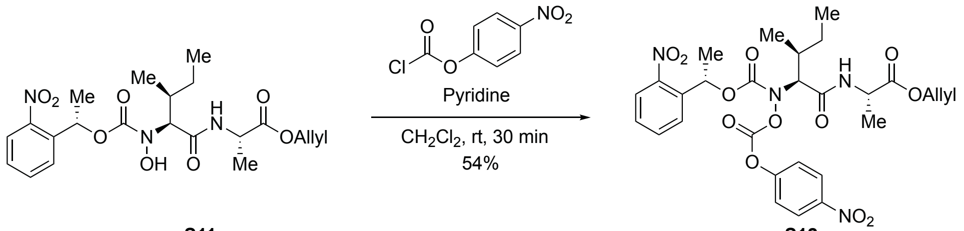

S11 **S12**

In a round bottom flask, **S11** (2.1 g, 4.7 mmol, 1.0 equiv) was dissolved in CH_2Cl_2 (40 mL). To the solution were added 4-nitrophenyl chloroformate (1.0 g, 5.1 mmol, 1.1 equiv) and pyridine (450 μL, 5.6 mmol, 1.2 equiv). After being allowed to stir for 30 min at rt, the reaction mixture was quenched with a 1 M HCl aqueous solution. The aqueous phase was extracted with CH_2Cl_2. The organic layer

was dried over Na_2SO_4. After concentration under reduced pressure, the residue was purified by reverse phase HPLC using the Biotage Sfär C18 column (30 μm, 100 Å pore size, 60 g) with a gradient of 50–95% CH_3CN in H_2O + 0.1% TFA over 8 column volumes at rt, with a flow rate of 50 mL/min. The pure product fractions were combined and lyophilized to obtain **S12** (1.5 g, 4.7 mmol, 54%) as a white solid.

^{1}H NMR (400 MHz, $CDCl_3$) δ 8.33 (d, J = 9.2 Hz, 2H), 7.99 (d, J = 8.1 Hz, 1H), 7.73 – 7.61 (m, 2H), 7.54 – 7.42 (m, 3H), 6.69 (s, 1H), 6.39 (q, J = 6.5 Hz, 1H), 5.91 (ddt, J = 17.2, 10.4, 5.7 Hz, 1H), 5.40 – 5.23 (m, 2H), 4.65 (ddt, J = 5.7, 2.8, 1.4 Hz, 2H), 4.58 (p, J = 7.1 Hz, 1H), 4.40 (d, J = 9.7 Hz, 1H), 2.34 (s, 1H), 1.74 (d, J = 6.5 Hz, 3H), 1.42 (d, J = 7.1 Hz, 3H), 0.98 (s, 3H), 0.89 (s, 3H).

^{13}C NMR (100 MHz, $CDCl_3$) δ 172.08, 167.41, 155.13, 153.84, 151.80, 147.73, 145.89, 136.76, 133.62, 131.47, 128.84, 127.10, 125.53, 124.66, 121.62, 118.86, 77.24, 72.19, 66.06, 48.28, 33.38, 24.90, 22.02, 17.99, 15.28, 10.87.

HRMS (ESI-Qq-TOF): m/z, $[M+H]^+$ calculated for $C_{28}H_{33}N_4O_{12}$: 617.2089, found 617.2079.

IR (cm^{-1}, ATR): 3369, 3080, 2967, 2878, 2362, 1812, 1734, 1675, 1524, 1346, 1191, 1156, 856.

(*S*)-1-(2-nitrophenyl)ethyl (*S*)-5-((*S*)-1-(allyloxy)-1-oxopropan-2-yl)-3-((*R*)-*sec*-butyl)-4,6-dioxo-1,2,5-oxadiazinane-2-carboxylate (S13)

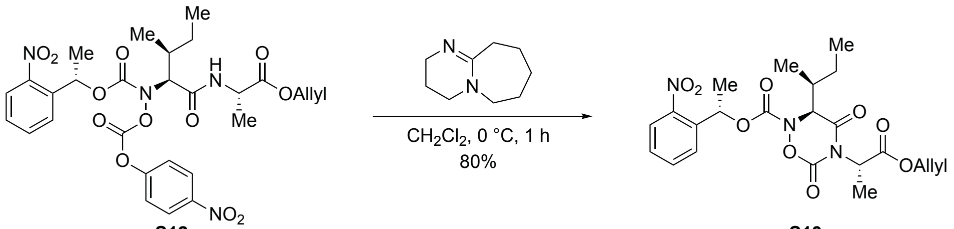

In a round bottom flask, **S12** (960.0 mg, 1.6 mmol, 1.0 equiv) was dissolved in CH_2Cl_2 (60 mL, 25 mM). To the solution was added DBU (232.4 µL, 1.6 mmol, 1.0 equiv) diluted in CH_2Cl_2 (5 mL) at 0 °C over a time period of 1 h. The reaction mixture was then quenched with a 1 M HCl aqueous solution. The aqueous phase was extracted with CH_2Cl_2. The organic layer was dried over Na_2SO_4. After concentration under reduced pressure, the residue was purified by flash column chromatography (pure CH_2Cl_2, Rf = 0.67 in hexane/EtOAc = 1:1) to afford **S13** (595 mg, 1.3 mmol, 80%) as an orange oil.

^{1}H NMR (400 MHz, $CDCl_3$) δ 8.01 (d, J = 8.2, 1H), 7.77 – 7.66 (m, 2H), 7.50 (ddd, J = 8.2, 5.0, 3.8 Hz, 1H), 6.43 (q, J = 6.5 Hz, 1H), 5.88 (ddt, J = 17.3, 10.4, 5.8 Hz, 1H), 5.42 – 5.24 (m, 2H), 5.16 (q, J = 7.1 Hz, 1H), 4.69 – 4.58 (m, 3H), 2.14 – 1.99 (m, 1H), 1.76 (d, J = 6.5 Hz, 3H), 1.64 (m, 1H), 1.57 (d, J = 7.1 Hz, 3H), 1.39 – 1.26 (m, 1H), 1.06 (d, J = 6.9 Hz, 3H), 0.92 (t, J = 7.4 Hz, 3H).

^{13}C NMR (100 MHz, $CDCl_3$) δ 168.32, 166.37, 154.43, 150.77, 147.48, 136.71, 134.00, 131.33, 128.92, 127.10, 124.67, 119.00, 72.43, 66.57, 64.34, 51.36, 34.57, 25.57, 22.09, 15.02, 14.87, 10.50.

HRMS (ESI-Qq-TOF): m/z, $[M+Na]^+$ calculated for $C_{22}H_{27}N_3NaO_9$: 500.1640, found 500.1633.

IR (cm^{-1}, ATR): 2969, 2880, 1720, 1526, 1339, 1250, 1201, 1057, 855.

(*S*)-2-((*S*)-3-((*R*)-*sec*-butyl)-2-(((*S*)-1-(2-nitrophenyl)ethoxy)carbonyl)-4,6-dioxo-1,2,5-oxadiazinan-5-yl)propanoic acid (11)

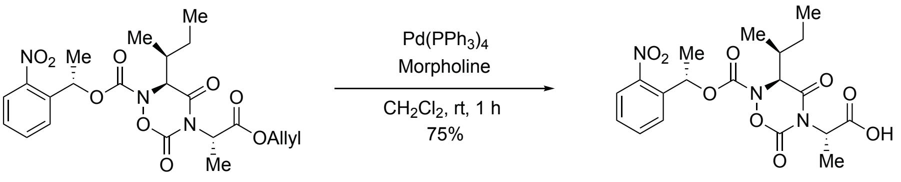

S13 **11**

In a round bottom flask, **S13** (521 mg, 1.1 mmol, 1.0 equiv) was dissolved in CH_2Cl_2 (10.9 mL, 0.1 M). To the solution were added tetrakis(triphenylphosphine)palladium (37.8 mg, 32.7 μmol, 0.03 equiv) and morpholine (94.1 μL, 1.09 mmol, 1.0 equiv). After being allowed to stir for 1 h at rt, the reaction mixture was quenched with a 10% citric acid aqueous solution. The aqueous phase was extracted with CH_2Cl_2. The organic layer was dried over Na_2SO_4. After concentration under reduced pressure, the residue was purified by reverse phase HPLC using the Shiseido Capcell Pak MGII C18 column (5 µm, 100 Å pore size, 250 × 20 mm) with a gradient of 50–95% CH_3CN in H_2O + 0.1% TFA over 30 min at rt, with a flow rate of 40 mL/min. The pure product fractions were combined and lyophilized to obtain **11** (355.4 mg, 0.813 mmol, 75 %) as a white solid.

^{1}H NMR (500 MHz, $CDCl_3$) δ 10.38 (s, 1H), 8.00 (d, *J* = 8.3, 1H), 7.74 – 7.67 (m, 2H), 7.50 (ddd, *J* = 8.3, 4.9, 3.7 Hz, 1H), 6.42 (q, *J* = 6.4 Hz, 1H), 5.27 – 5.15 (m, 1H), 4.64 (d, *J* = 8.9 Hz, 1H), 2.10 – 2.00 (m, 1H), 1.72 (d, *J* = 6.4 Hz, 3H), 1.62 (m, 1H), 1.57 (d, *J* = 7.1 Hz, 3H), 1.32 (m, 1H), 1.06 (d, *J* = 6.8 Hz, 3H), 0.91 (t, *J* = 7.4 Hz, 3H).

^{13}C NMR (125 MHz, $CDCl_3$) δ 174.85, 166.31, 154.56, 150.65, 147.44, 136.56, 134.09, 128.97, 127.20, 124.65, 72.50, 64.25, 50.94, 34.54, 25.63, 22.04, 14.98, 14.57, 10.46.

HRMS (ESI-Qq-TOF): m/z, $[M+NH_4]^+$ calculated for $C_{19}H_{27}N_4O_9$: 455.1773, found 455.1770.

IR (cm^{-1}, ATR): 2970, 2881, 1718, 1526, 1339, 1258, 1208, 1056, 856.

4. Mechanistic Studies

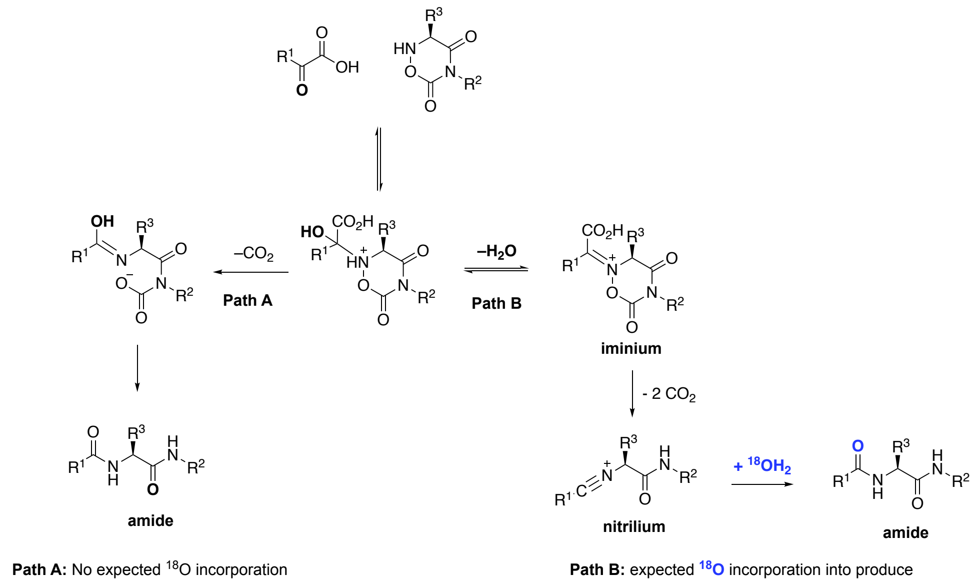

Based on the established mechanism of KAHA ligation, we proposed two possible mechanistic pathways for the cyclic hydroxylamine. The ligation begins with the nucleophilic attack of the hydroxylamine nitrogen to the carbonyl carbon of *α*-ketoacid, forming the common tetrahedral intermediate. In Path A, decarboxylation results in the imine intermediate, which releases another equivalent of CO_2, forming the amide product. In Path B, subsequent elimination of water of leads to formation of an iminium. Due to the presence of a carbamate in the cyclic hydroxylamine structure, the rearrangement proceeds with the loss of two equivalents of CO_2. Simultaneous ring opening offers a nitrilium intermediate, which undergoes hydrolysis to yield the desired amide.

To test this hypothesis, we performed a O^{18} labelling experiment – the small molecule KAHA ligation in presence of $^{18}OH_2$. In principle, the path B should provide the amide product incorporating the O^{18} from $^{18}OH_2$ in the solvent, which should not be observed for path A.

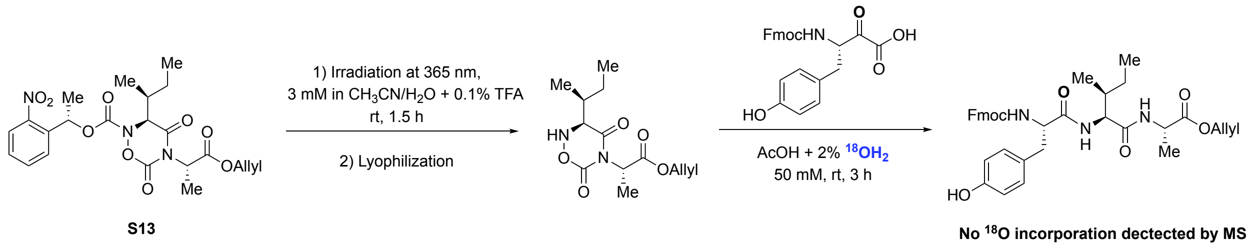

The protected cyclic hydroxylamine **S13** (2.9 mg, 6.07 *μ*mol, 3.0 equiv) was dissolved in CH_3CN/H_2O + 0.1% TFA (2 mL, 3 mM) and irradiated at 365 nm at rt for 1.5 h. The lyophilized deprotected hydroxylamine crude was dissolved in AcOH (20 *μ*L, 300 mM). The Fmoc-Tyr-COOH ketoacid (0.9 mg, 2.02 *μ*mol, 1.0 equiv) was added. The total volume was diluted with AcOH to achieve a final concentration of 50 mM for ketoacid, with 2% of $^{18}OH_2$. After incubation at rt for 3 h, the formation of unlabeled ligation product was observed in LCMS. This result suggested that Path A is the more plausible mechanism.

MS (ESI): m/z, $[M+H]^+$ calculated for $C_{36}H_{42}N_3O_7$: 628.75, found 628.36.

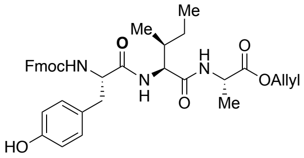

Predicted mass for $C_{36}H_{42}N_3O_7^+$ $[M+H]^+$: 628.7455

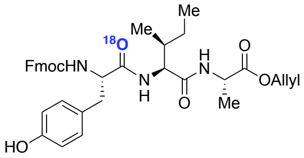

Predicted mass for $C_{36}H_{42}N_3O_6{}^{18}O^+$ $[M+H]^+$: 630.7456

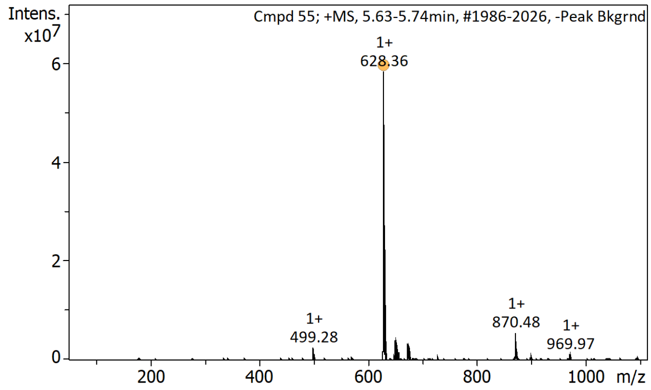

5. Synthesis of Ubiquitin

5.1. Ac-Ub(K48-Aboc)-DH$_6$ (10a)

5.1.1. Ac-Ub-(1-43)-Leu-*α*-ketoacid (8a)

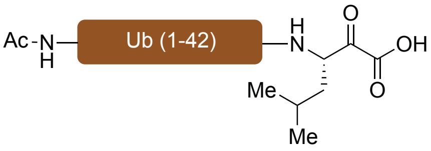

8a

Methionine (Met1) residue was substituted by norleucine (Nle) residue.[3] Ac-Ub-(1-43)-Leu-*α*-ketoacid **8a** was synthesized on Rink amide polystyrene resin preloaded with protected Fmoc-Leu-*α*-ketoacid with a substitution capacity of 0.26 mmol/g.[4] The synthesis was performed on a 0.10 mmol scale (385 mg resin, 1.0 equiv) by automated Fmoc-SPPS up to AcHN-Nle1 using the procedure described in General Methods. N-terminal Acetyl capping was induced by subjecting the peptide to capping conditions (20% Ac_2O in DMF, 2 x 5 min) at the end of the synthesis after the final Fmoc deprotection. After cleavage from resin, purification was performed by preparative HPLC using the Reprosil-Pur C18 column (5 µm, 120 Å pore size, 250 x 50 mm) with a gradient of 5–95% CH_3CN in H_2O + 0.1% TFA over 30 min at rt, with a flow rate of 40 mL/min. The product fractions were pooled and lyophilized to obtain **8a** (65.7 mg, 13.4 µmol, 13.4% yield for peptide synthesis, resin cleavage, and purification steps). Analytical HPLC measurement confirmed the purity using the Shiseido Capcell Pak UG80 C18 UG 80 (5 µm, 120 Å pore size, 250 x 4.6 mm) with a gradient of 5–95% CH_3CN in H_2O + 0.1% TFA over 14 min at rt, with a flow rate of 1 mL/min.

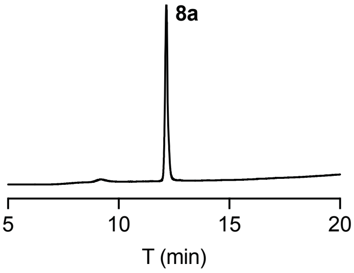

LC-MS confirmed the exact mass, from top to bottom: observed mass spectrum, deconvoluted mass spectrum, close-up view of deconvoluted mass spectrum, simulated mass spectrum.

HRMS (ESI): calculated for $C_{218}H_{364}N_{56}O_{71}$, 4905.6; measured 4904.7.

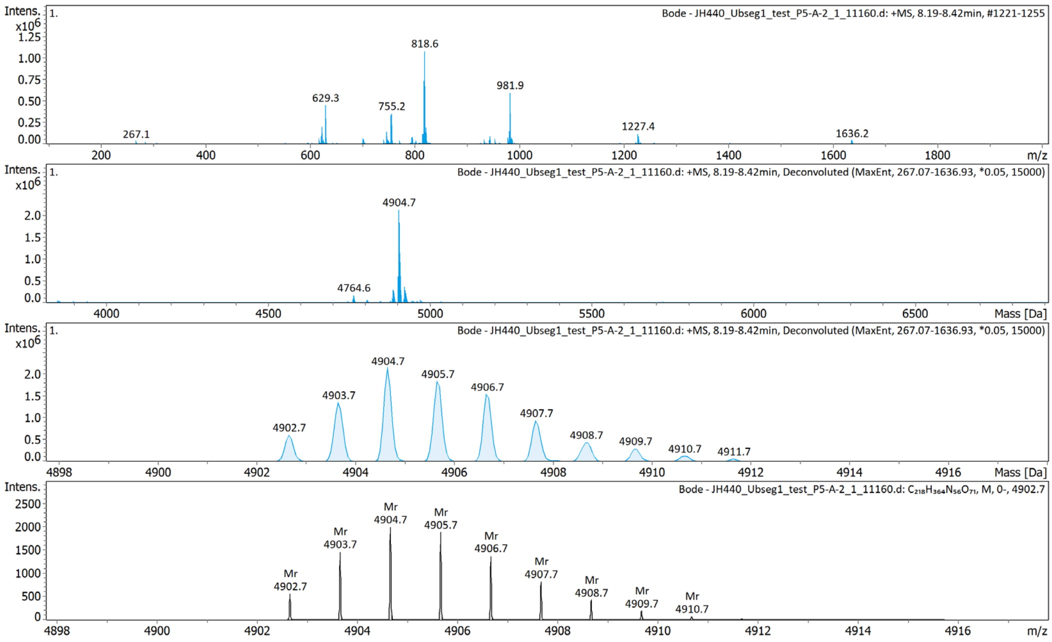

5.1.2. Photoprotected-Ub(44-76)-(K48-Aboc)-DH_6 (S14)

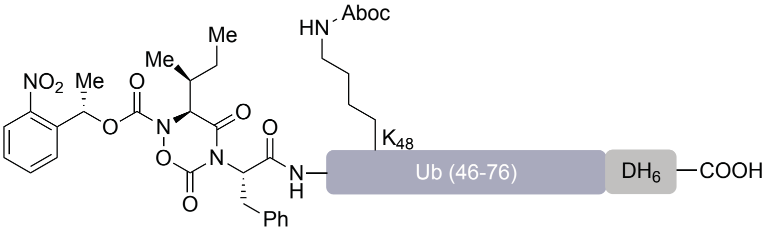

S14

Photoprotected hydroxylamine Ub(44-76)-(K48-Aboc)-DH_6 **S14** was synthesized on 2-chlorotritylchloride polystyrene resin preloaded with Fmoc-His(Trt)-OH with a substitution capacity of 0.3 mmol/g. The synthesis was performed on a 0.3 mmol scale (1.0 g resin, 1.0 equiv) by automated Fmoc-SPPS up to Ala46 using the procedure described in General Methods. K48 modification was introduced with the Fmoc-Lys(Aboc)-OH (2.00 equiv) using HATU (1.98 equiv) and NMM (4.00 equiv) in DMF (100 mM) for 2 h at rt.[5] The resin was coupled with the photoprotected Ile-Phe hydroxylamine building block **7** (1.5 equiv) using Oxyma (1.5 equiv) and DIC (1.5 equiv) in DMF (100 mM) for 2 h.

After cleavage from resin, purification was performed by preparative HPLC using the Reprosil-Pur C18 column (5 µm, 120 Å pore size, 250 x 50 mm) with a gradient of 15 to 75% CH_3CN in H_2O + 0.1% TFA over 30 min at rt, with a flow rate of 40 mL/min. The pure product fractions were combined and lyophilized to obtain **S14** (559 mg, 111 µmol, 37% yield for peptide synthesis, resin cleavage, and purification steps). Analytical HPLC measurement confirmed the purity using the Reprosil-Pur C18 column (5 µm, 120 Å pore size, 250 x 4.6 mm) with a gradient of 20–60% CH_3CN in H_2O + 0.1% TFA over 14 min at rt, with a flow rate of 1 mL/min.

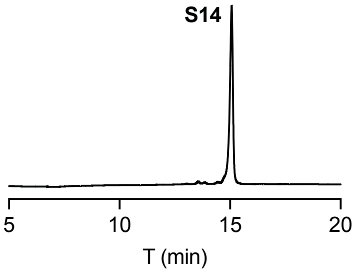

LC-MS confirmed the exact mass, from top to bottom: observed mass spectrum, deconvoluted mass spectrum, close-up view of deconvoluted mass spectrum, simulated mass spectrum.

HRMS (ESI): calculated for $C_{219}H_{332}N_{70}O_{68}$, 5033.5; measured 5032.4.

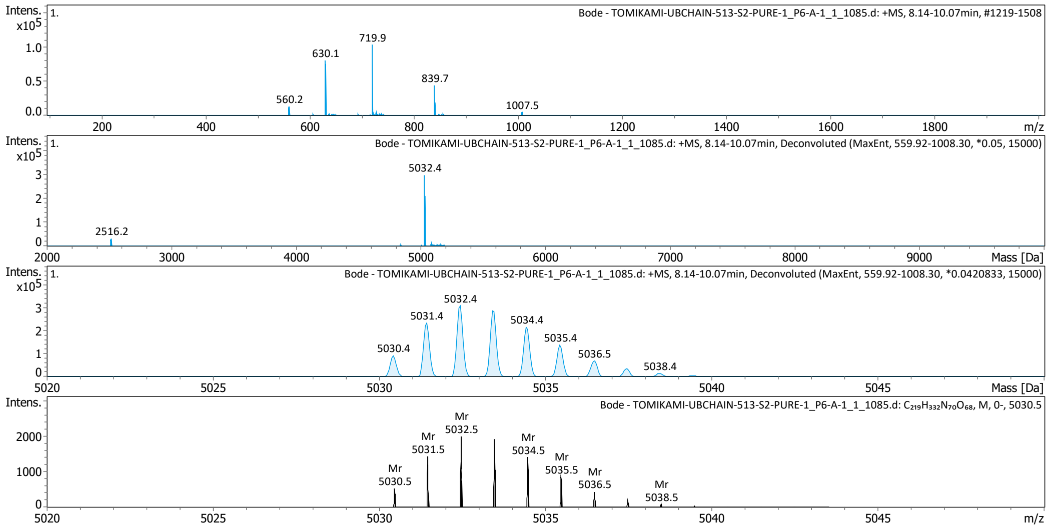

5.1.3. Photodeprotection - Ub(44-76)-(K48-Aboc)-DH$_6$ (9a)

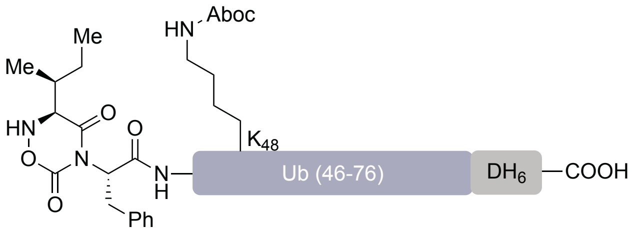

9a

Photoprotected hydroxylamine peptide **S14** (51.5 mg, 10.2 μmol, 1.00 equiv) was redissolved in CH_3CN/H_2O = 1:1 + 0.1% TFA (0.5 mM) and irradiated for 2 h at 365 nm with a hand-held UV lamp, cooled with ice. The deprotected peptide was lyophilized to obtain **9a** and used for the next reaction without further purification. Analytical HPLC measurement confirmed the purity using the Reprosil-Pur C18 column (5 µm, 120 Å pore size, 250 x 4.6 mm) with a gradient of 20–60% CH_3CN in H_2O + 0.1% TFA over 14 min at rt, with a flow rate of 1 mL/min.

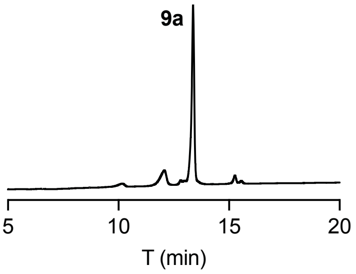

LC-MS confirmed the exact mass, from top to bottom: observed mass spectrum, deconvoluted mass spectrum, close-up view of deconvoluted mass spectrum, simulated mass spectrum.

HRMS (ESI): calculated for $C_{210}H_{325}N_{69}O_{64}$, 4840.3; measured 4840.4.

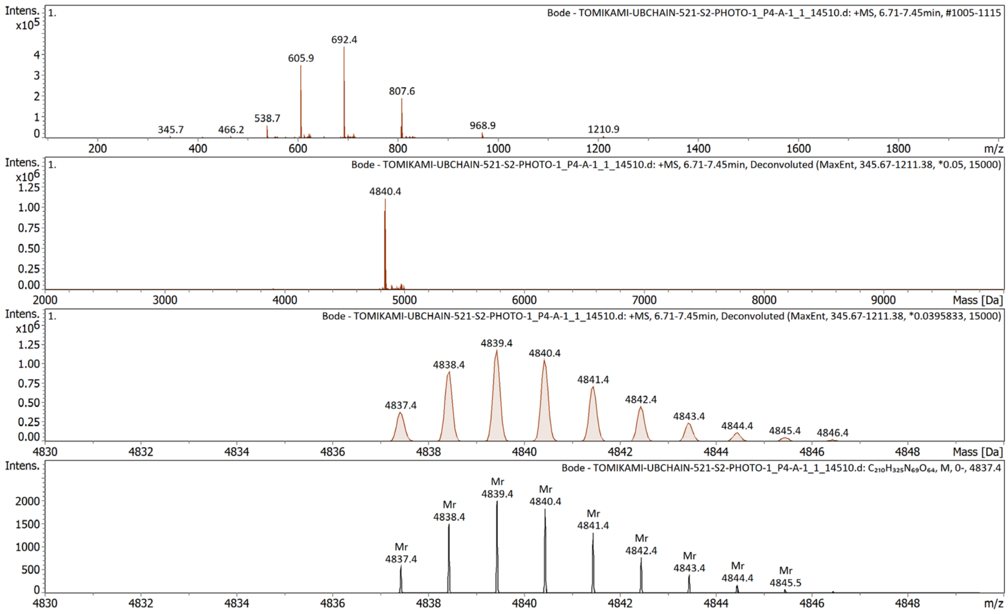

5.1.4. KAHA ligation - Ac-Ub(K48-Aboc)-DH_6 (10a)

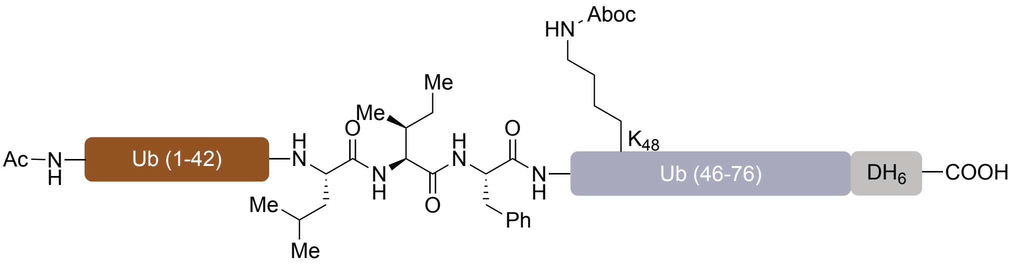

10a

Ac-Ub-(1-43)-Leu-*α*-ketoacid **8a** (52.7 mg, 10.7 *μ*mol, 1.05 equiv) and deprotected hydroxylamine peptide **9a** (10.2 *μ*mol, 1.00 equiv) were dissolved in AcOH/HFIP (1:1) + 1% H_2O (20 mM) and incubated at rt for 48 h. The ligation was monitored by analytical HPLC using the Reprosil-Pur C18 column (5 µm, 120 Å pore size, 250 x 4.6 mm) with a gradient of 20–60% CH_3CN in H_2O + 0.1% TFA over 14 min at rt, with a flow rate of 1 mL/min.

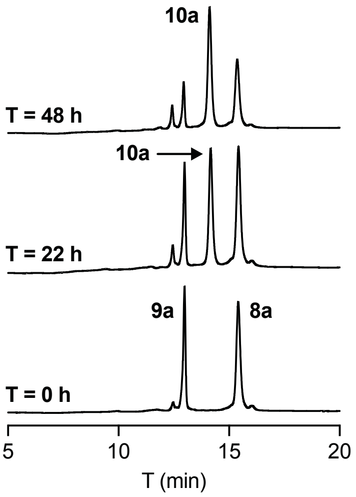

The ligation product was purified by preparative HPLC using the Reprosil-Pur C18 column (5 µm, 120 Å pore size, 250 x 50 mm) with a gradient of 20–60% CH_3CN in H_2O + 0.1% TFA over 30 min at rt, with a flow rate of 40 mL/min. The pure product fractions were combined and lyophilized to obtain **10a** (47.3 mg, 4.9 *μ*mol, 48% yield). Analytical HPLC measurement confirmed the purity of ligated product **10a** using the Reprosil-Pur C18 column (5 µm, 120 Å pore size, 250 x 4.6 mm) with a gradient of 20–60% CH_3CN in H_2O + 0.1% TFA over 14 min at rt, with a flow rate of 1 mL/min.

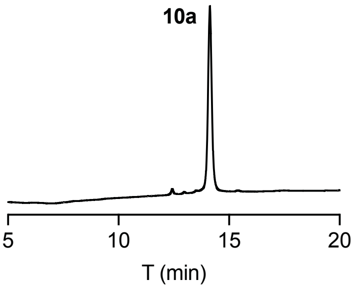

LC-MS confirmed the exact mass, from top to bottom: observed mass spectrum, deconvoluted mass spectrum, close-up view of deconvoluted mass spectrum, simulated mass spectrum.

HRMS (ESI): calculated for $C_{426}H_{690}N_{125}O_{131}$, 9658.9; measured 9657.1.

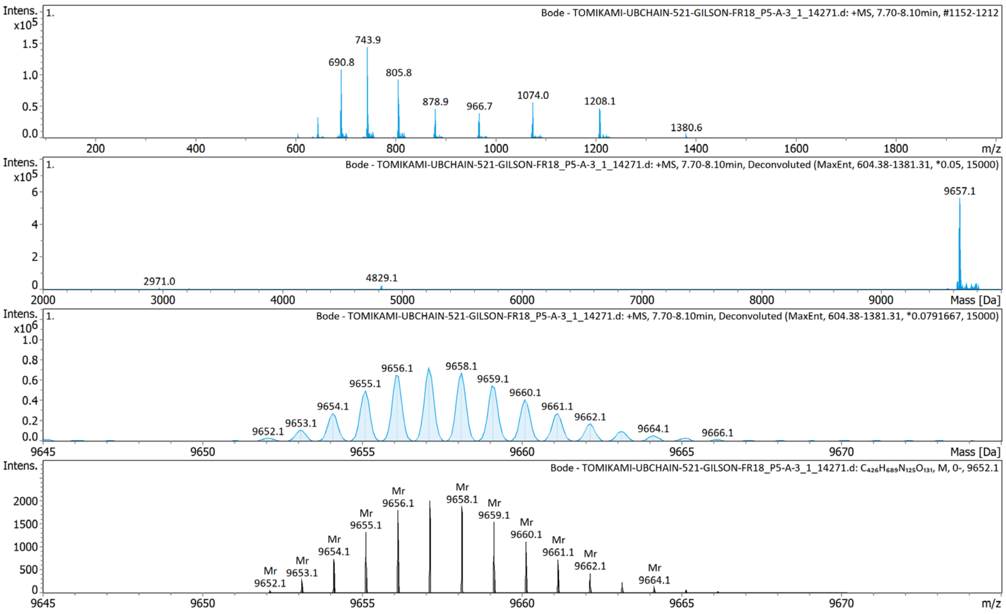

5.2. Desthiobiotin-Ub(K48-Aboc, K63-Aboc)-OH (10b)

5.2.1. dsBt-Ub-(1-43)-Leu-*α*-ketoacid (8b)

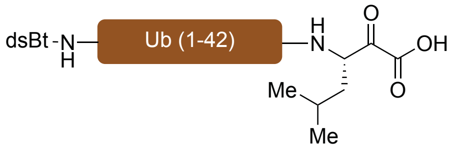

8b

Methionine (Met1) residue was substituted by norleucine (Nle) residue.[3] dsBt-Ub-(1-43)-Leu-*α*-ketoacid **8b** was synthesized on Rink amide polystyrene resin preloaded with protected Fmoc-Leu-*α*-ketoacid with a substitution capacity of 0.25 mmol/g.[4] The synthesis performed on 0.25 mmol scale (1 g resin, 1.0 equiv) by automated Fmoc-SPPS using the procedure described in General Methods. The d-Desthiobiotin (2.0 equiv) was coupled using HATU (1.96 equiv) and DIPEA (4.0 equiv) in DMF (100 mM) for 2 h at rt. After cleavage from resin, purification was performed by preparative HPLC using the Reprosil-XR C8 column (5 µm, 120 Å pore size) with a gradient of 20–95% CH_3CN in H_2O + 0.1% TFA over 40 min at rt, with a flow rate of 60 mL/min. The product fractions were pooled and lyophilized to obtain **8b** (211 mg, 41.7 µmol, 17% yield for peptide synthesis, resin cleavage, and purification steps). Analytical HPLC measurement confirmed the purity using the Reprosil-Pur C18 column (5 µm, 120 Å pore size, 250 x 4.6 mm) with a gradient of 20–60% CH_3CN in H_2O + 0.1% TFA over 14 min at rt, with a flow rate of 1 mL/min.

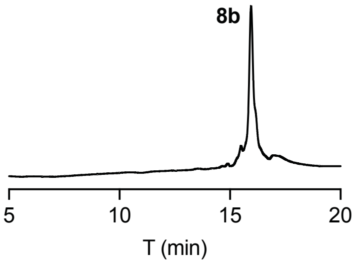

LC-MS confirmed the exact mass, from top to bottom: observed mass spectrum, deconvoluted mass spectrum, close-up view of deconvoluted mass spectrum, simulated mass spectrum.

HRMS (ESI): calculated for $C_{226}H_{378}N_{58}O_{72}$, 5059.8; measured 5058.8.

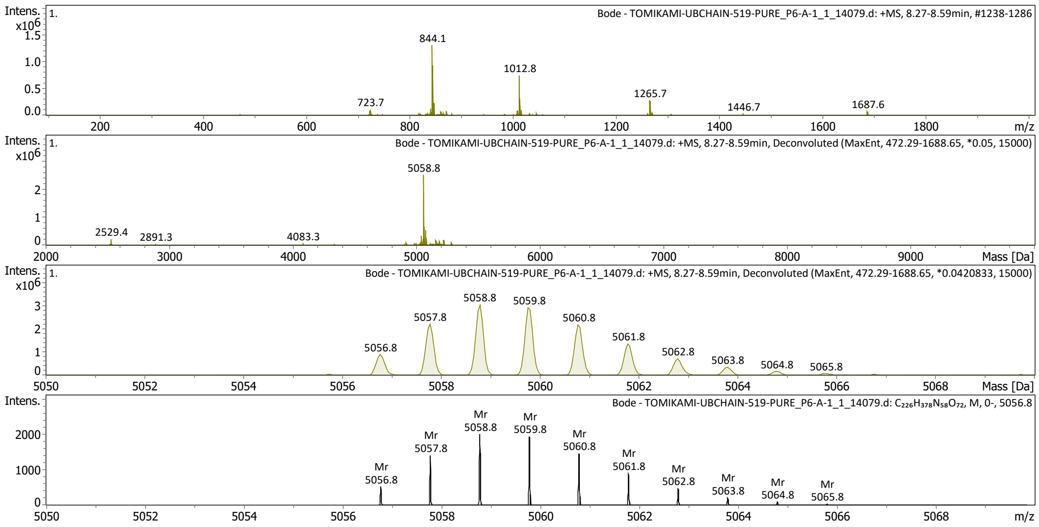

5.2.2. Photoprotected-Ub(44-76)-(K48-Aboc, K63-Aboc)-OH (S15)

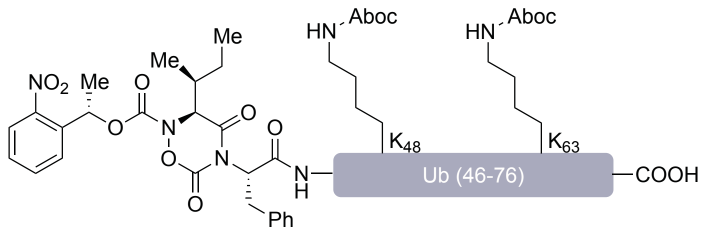

S15

Photoprotected hydroxylamine Ub(44-76)-(K48-Aboc, K63-Aboc)-OH **S15** was synthesized on 2-chlorotritylchloride polystyrene resin preloaded with Fmoc-Gly-OH with a substitution capacity of 0.32 mmol/g. The synthesis was performed on a 0.2 mmol scale (625 mg resin, 1.0 equiv) by automated Fmoc-SPPS up to Ala46 using the procedure described in General Methods. K48 and K63 modifications were introduced with the Fmoc-Lys(Aboc)-OH (2.00 equiv) using HATU (1.98 equiv) and NMM (4.00 equiv) in DMF (100 mM) for 2h, respectively.[5] The resin was coupled with the photoprotected Ile-Phe hydroxylamine building block **7** (1.5 equiv) using Oxyma (1.5 equiv) and DIC (1.5 equiv) in DMF (100 mM) for 2 h.

After cleavage from resin, purification was performed by preparative HPLC using the Reprosil-Pur C18 column (5 µm, 120 Å pore size, 250 x 50 mm) with a gradient of 15 to 75% CH_3CN in H_2O + 0.1% TFA over 30 min at rt, with a flow rate of 40 mL/min. The pure product fractions were combined and directly used for the following photodeprotection without lyophilization. Analytical HPLC measurement confirmed the purity using the Reprosil-Pur C18 column (5 µm, 120 Å pore size, 250 x 4.6 mm) with a gradient of 20–60% CH_3CN in H_2O + 0.1% TFA over 14 min at rt, with a flow rate of 1 mL/min.

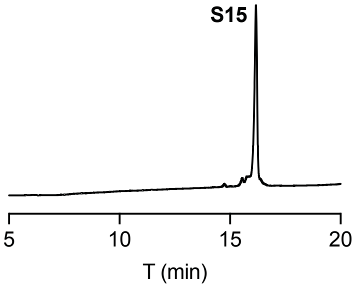

LC-MS confirmed the exact mass, from top to bottom: observed mass spectrum, deconvoluted mass spectrum, close-up view of deconvoluted mass spectrum, simulated mass spectrum.

HRMS (ESI): calculated for $C_{184}H_{294}N_{52}O_{62}$, 4226.7; measured 4226.2.

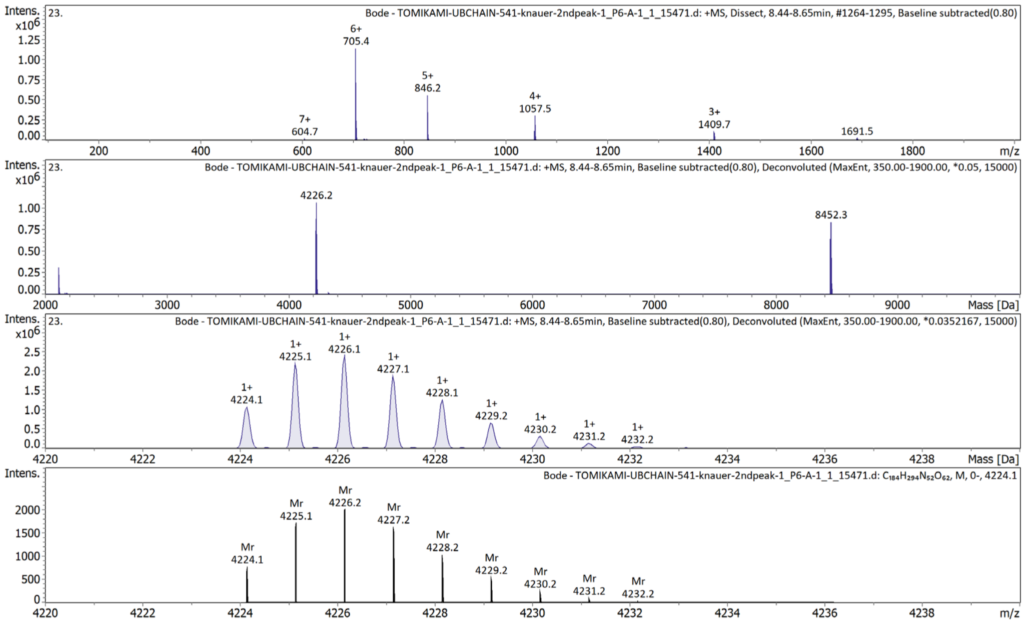

5.2.3. Photodeprotection - Ub(44-76)-(K48-Aboc, K63-Aboc)-OH (9b)

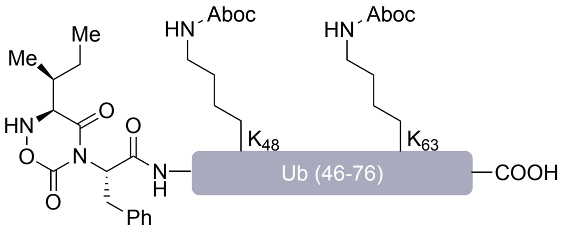

9b

Photoprotected hydroxylamine peptide **S15** was irradiated for 3 h at 365 nm with a hand-held UV lamp, cooled with ice. The deprotected peptide was lyophilized to obtain **9b** (65 mg, 16 µmol, 8% yield for peptide synthesis, resin cleavage, and purification steps) and used for the next reaction without further purification. Analytical HPLC measurement confirmed the purity using the Reprosil-Pur C18 column (5 µm, 120 Å pore size, 250 x 4.6 mm) with a gradient of 20–60% CH_3CN in H_2O + 0.1% TFA over 14 min at rt, with a flow rate of 1 mL/min.

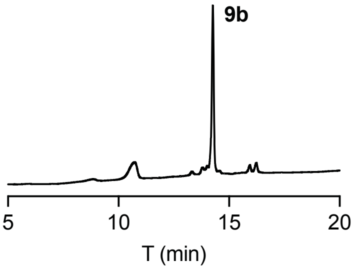

LC-MS confirmed the exact mass, from top to bottom: observed mass spectrum, deconvoluted mass spectrum, close-up view of deconvoluted mass spectrum, simulated mass spectrum.

HRMS (ESI): calculated for $C_{175}H_{287}N_{51}O_{58}$, 4033.5; measured 4033.1.

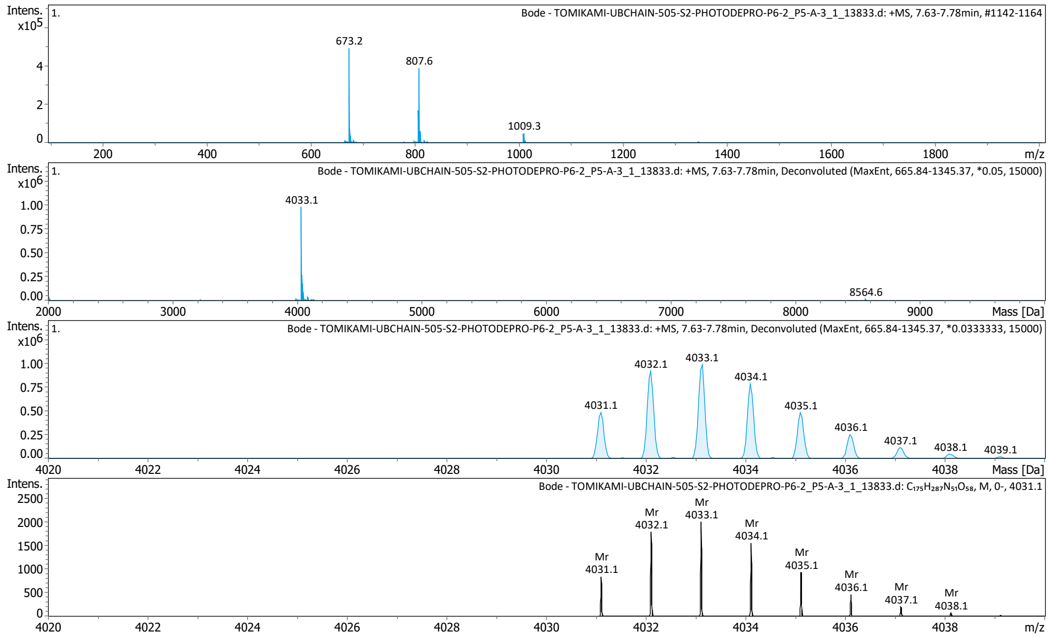

5.2.4. KAHA ligation - dsBt-Ub(K48-Aboc, K63-Aboc)-OH (10b)

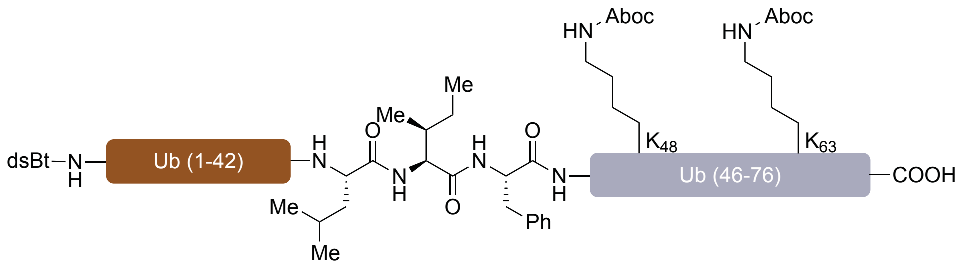

10b

dsBt-Ub-(1-43)-Leu-*α*-ketoacid **8b** (8.28 mg, 1.64 *μ*mol, 1.2 equiv) and deprotected hydroxylamine peptide **9b** (5.5 mg, 1.36 *μ*mol, 1.0 equiv) were dissolved in AcOH/HFIP (1:1) w/ 1% H_2O (20 mM) and incubated at rt for 4 d. The ligation was monitored by analytical HPLC using the Reprosil-Pur C18 column (5 µm, 120 Å pore size, 250 x 4.6 mm) with a gradient of 20–60% CH_3CN in H_2O + 0.1% TFA over 14 min at rt, with a flow rate of 1 mL/min.

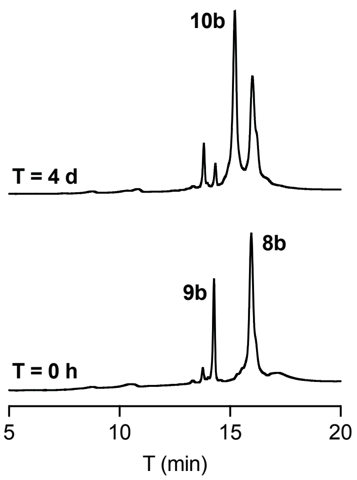

The ligation product was purified by preparative HPLC using the Reprosil-Pur C18 column (5 µm, 120 Å pore size, 250 x 50 mm) with a gradient of 20–60% CH_3CN in H_2O + 0.1% TFA over 30 min at 60 °C, with a flow rate of 40 mL/min. The pure product fractions were combined and lyophilized to obtain **10b** (3.2 mg, 0.354 *μ*mol, 26% yield). Analytical HPLC measurement confirmed the purity of ligated product **10b** using the Reprosil-Pur C18 column (5 µm, 120 Å pore size, 250 x 4.6 mm) with a gradient of 20–60% CH_3CN in H_2O + 0.1% TFA over 14 min at rt, with a flow rate of 1 mL/min.

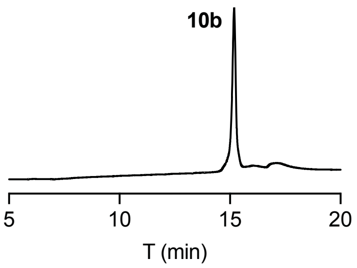

LC-MS confirmed the exact mass, from top to bottom: observed mass spectrum, deconvoluted mass spectrum, close-up view of deconvoluted mass spectrum, simulated mass spectrum.

HRMS (ESI): calculated for $C_{399}H_{665}N_{109}O_{126}$, 9005.3; measured 9004.9.

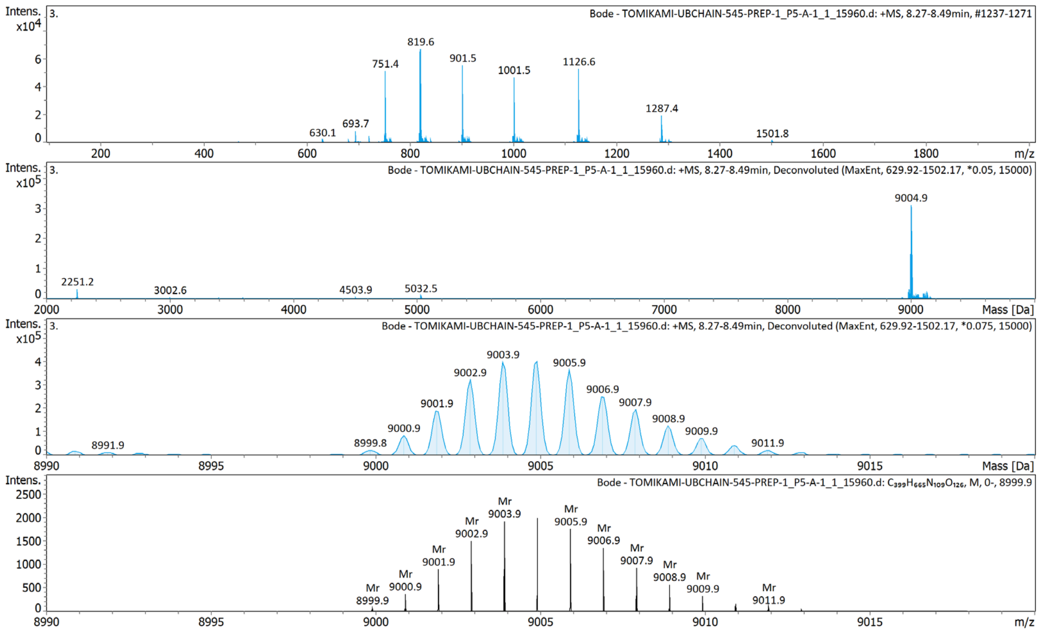

6. Synthesis of Tirzepatide (14)

6.1. Tirzepatide-(1-16)-Lys-*α*-ketoacid (12)

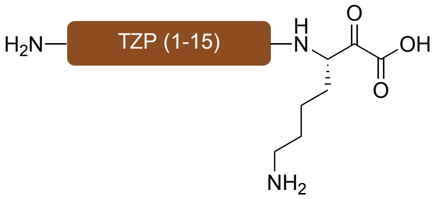

12

Tirzepatide-(1-16)-Lys-*α*-ketoacid **12** was synthesized on Rink amide polystyrene resin preloaded with protected Fmoc-Lys-*α*-ketoacid with a substitution capacity of 0.19 mmol/g.[6] The synthesis was performed on a 0.2 mmol scale (1.07 g resin, 1.0 equiv) by automated Fmoc-SPPS using the procedure described in General Methods. After the final Fmoc deprotection and cleavage from resin, purification was performed by preparative HPLC using the Shiseido Capcell Pak MGII C18 column (5 µm, 100 Å pore size, 250 × 20 mm) with a gradient of 10–95% CH_3CN in H_2O + 0.1% TFA over 30 min at rt, with a flow rate of 40 mL/min. The product fractions were pooled and lyophilized to obtain **12** (73.4 mg, 40.0 µmol, 20% yield for peptide synthesis, resin cleavage, and purification steps). Analytical HPLC measurement confirmed the purity using the Agilent Eclipse XDB C8 column (5 µm, 80 Å pore size, 150 × 4.6 mm) with a gradient of 20–95% CH_3CN in H_2O + 0.1% TFA over 14 min at rt, with a flow rate of 1 mL/min.

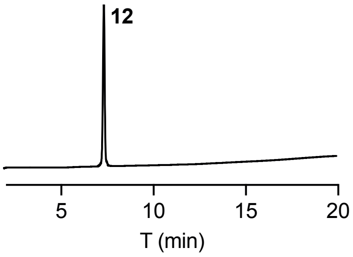

LC-MS confirmed the exact mass, from top to bottom: observed mass spectrum, deconvoluted mass spectrum, close-up view of deconvoluted mass spectrum, simulated mass spectrum.

HRMS (ESI): calculated for $C_{83}H_{121}N_{17}O_{30}$, 1837.0; measured 1836.8.

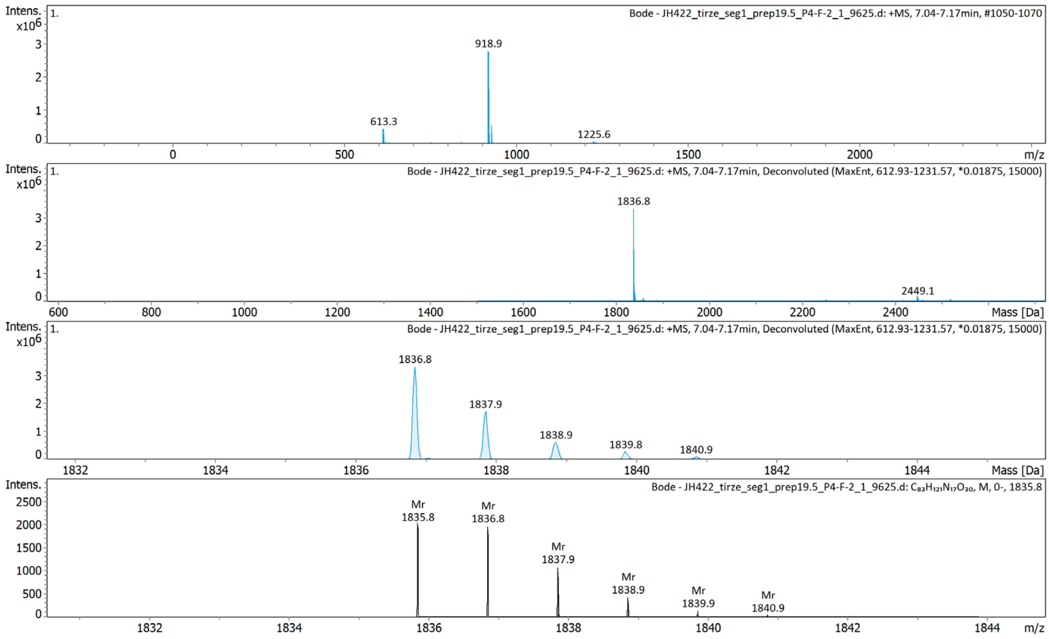

6.2. Photoprotected-Tirzepatide-(17-39) (S16)

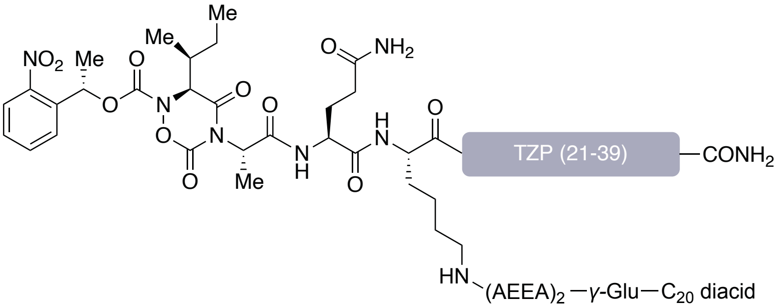

S16

Photoprotected hydroxylamine tirzepatide (17-39) was synthesized on Rink amide polystyrene resin preloaded with Fmoc-Ser-OH with a substitution capacity of 0.30 mmol/g. The synthesis was performed on 0.6 mmol scale (2 g resin, 1.0 equiv) by automated Fmoc-SPPS up to Lys20 (Alloc-Lys(Fmoc)-OH) using the procedure described in General Methods. The loading was determined to be 0.17 mmol/g. An aliquot of the resin (500 mg, 87 µmol) was deprotected and coupled with the fatty acid side chain building block *t*BuO-Ara-Glu(AEEA-AEEA-OH)-O*t*Bu (3.0 equiv) using DIC (3.0 equiv) and Oxyma (3.0 equiv) in DMF (100 mM) for 2 h. After washing with DMF and CH_2Cl_2, the resin was treated twice with tetrakis-(triphenylphosphin)-palladium (0.1 equiv) and phenyl silane (20.0 equiv) dissolved in CH_2Cl_2 (100 mM) to remove the *N*-terminal Alloc protecting group. After quenching with a solution of sodium diethyldithiocarbamate in DMF (10 wt%), the resin was washed with DMF and CH_2Cl_2. Fmoc-Gln(Trt)-OH was coupled with DIC (3.0 equiv) and Oxyma (3.0 equiv) in DMF (100 mM) for 45 min, coupling repeated twice. The loading was determined to be 0.12 mmol/g. An aliquot of the resin (250 mg, 43 µmol) was deprotected and coupled with the photoprotected Ile-Ala hydroxylamine building block **11** (1.0 equiv) using Oxyma (1.0 equiv) and DIC (1.0 equiv) in DMF (100 mM) for overnight, coupling repeated twice.

After cleavage from resin, purification was performed by preparative HPLC using the Shiseido Capcell Pak MGII C18 column (5 µm, 100 Å pore size, 250 × 20 mm) with a gradient of 50–95% CH_3CN in H_2O + 0.1% TFA over 30 min at rt, with a flow rate of 40 mL/min. The pure product fractions were combined and lyophilized to obtain **S16** (30.0 mg, 9.2 µmol, 12% yield for peptide synthesis, resin cleavage, and purification steps). Analytical HPLC measurement confirmed the purity using the Agilent Eclipse XDB C8 column (5 µm, 80 Å pore size, 150 × 4.6 mm) with a gradient of 20–95% CH_3CN in H_2O + 0.1% TFA over 14 min at 60 °C, with a flow rate of 1 mL/min.

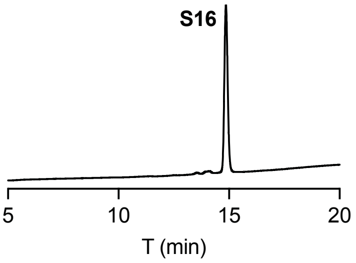

LC-MS confirmed the exact mass, from top to bottom: observed mass spectrum, deconvoluted mass spectrum, close-up view of deconvoluted mass spectrum, simulated mass spectrum.

HRMS (ESI): calculated for $C_{153}H_{234}N_{32}O_{46}$, 3257.7; measured 3257.7.

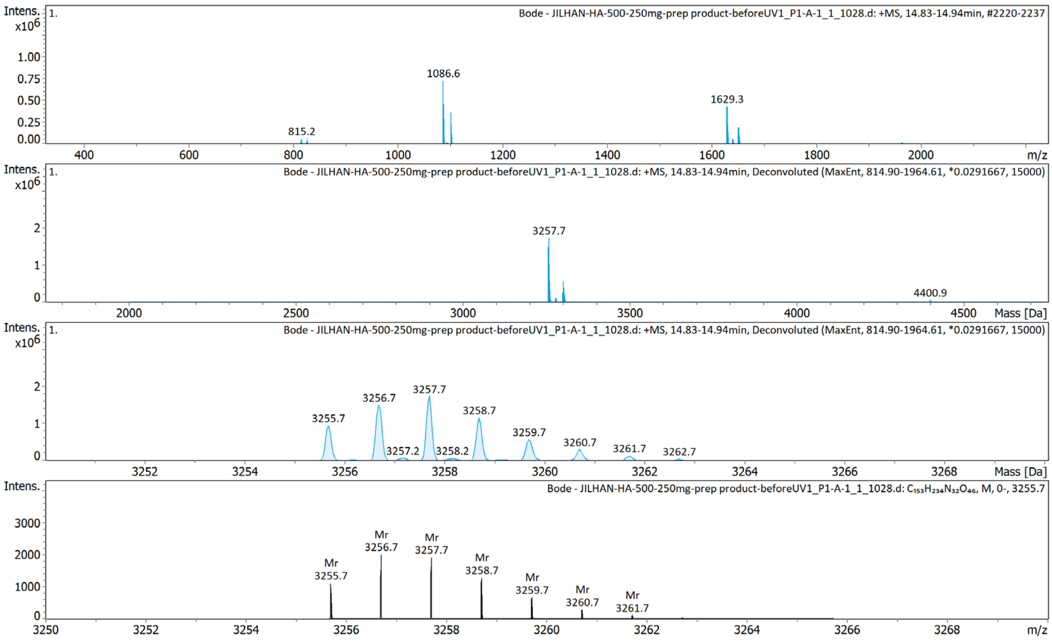

6.3. Photodeprotection - Tirzepatide-(17-39) (13)

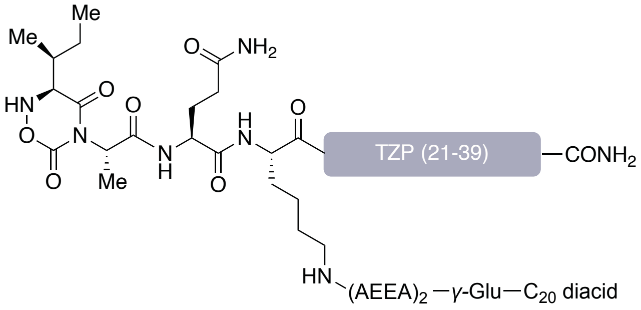

13

Photoprotected hydroxylamine peptide **S16** was redissolved in CH_3CN/H_2O = 1:1 + 0.1% TFA (1 mM) and irradiated for 1.5 h at 365 nm with a hand-held UV lamp, cooled with ice. The deprotected peptide was lyophilized to obtain **13** and used for the next reaction without further purification. Analytical HPLC measurement confirmed the purity using the Agilent Eclipse XDB C8 column (5 µm, 80 Å pore size, 150 × 4.6 mm) with a gradient of 20–95% CH_3CN in H_2O + 0.1% TFA over 14 min at 60 °C, with a flow rate of 1 mL/min.

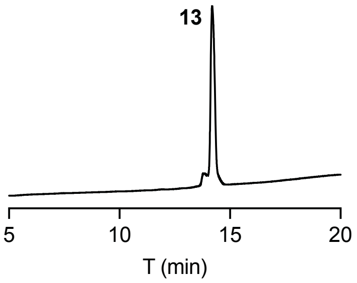

LC-MS confirmed the exact mass, from top to bottom: observed mass spectrum, deconvoluted mass spectrum, close-up view of deconvoluted mass spectrum, simulated mass spectrum.

HRMS (ESI): calculated for $C_{144}H_{227}N_{31}O_{42}$, 3064.6; measured 3063.7.

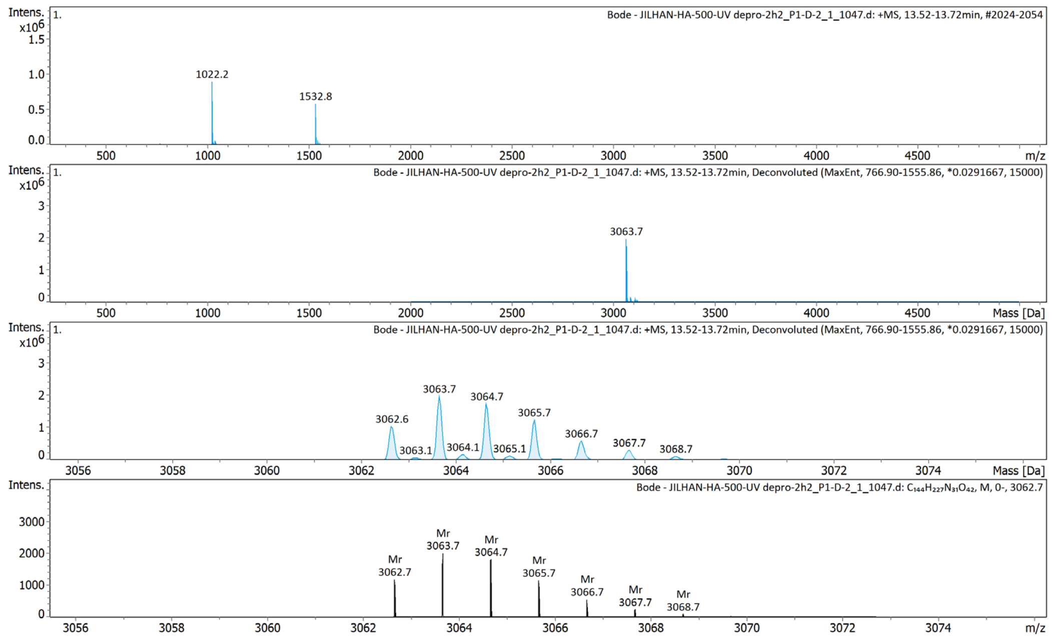

6.4. KAHA ligation - Tirzepatide (14)

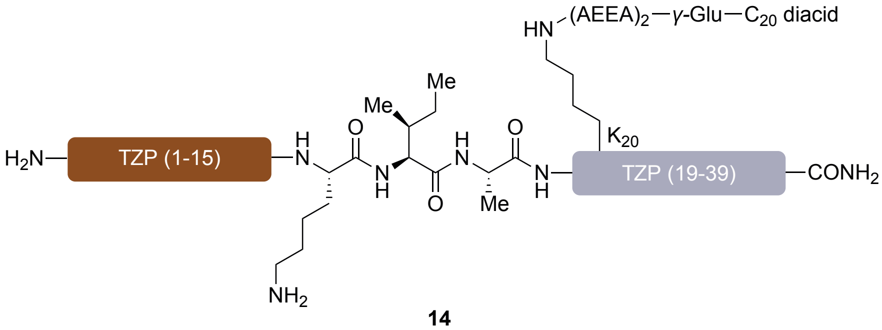

14

Tirzepatide-(1-16)-Lys-*α*-ketoacid **12** (2.8 mg, 1.5 *μ*mol, 1.0 equiv) and deprotected hydroxylamine peptide **13** (4.7 mg, 1.5 *μ*mol, 1.0 equiv) were dissolved in AcOH (40 mM) and incubated at rt for 48 h. The ligation was monitored by analytical HPLC using the Agilent Eclipse XDB C8 column (5 µm, 80 Å pore size, 150 × 4.6 mm) with a gradient of 20–95% CH_3CN in H_2O + 0.1% TFA over 14 min at 60 °C, with a flow rate of 1 mL/min.

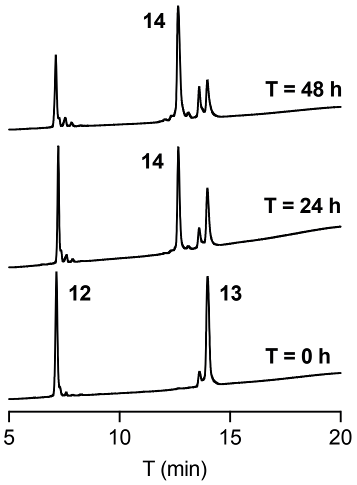

The ligation product was purified by preparative HPLC using the Shiseido Capcell Pak MGII C18 column (5 *μ*m, 100 Å pore size, 250 × 20 mm) with a gradient of 30–95% CH_3CN in H_2O + 0.1% TFA over 30 min at rt, with a flow rate of 40 mL/min. The pure product fractions were combined and lyophilized to obtain **14** (3.1 mg, 0.64 µmol, 42% yield). Analytical HPLC measurement confirmed the purity of ligated product **14** using the Agilent Eclipse XDB C8 column (5 µm, 80 Å pore size, 150 × 4.6 mm) with a gradient of 20–95% CH_3CN in H_2O + 0.1% TFA over 14 min at 60 °C, with a flow rate of 1 mL/min.

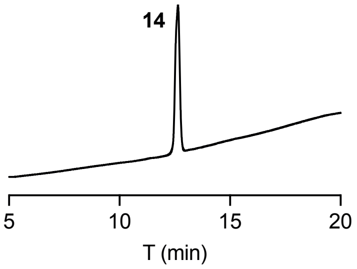

LC-MS confirmed the exact mass, from top to bottom: observed mass spectrum, deconvoluted mass spectrum, close-up view of deconvoluted mass spectrum, simulated mass spectrum.

HRMS (ESI): calculated for $C_{225}H_{348}N_{48}O_{68}$, 4813.5; measured 4812.5.

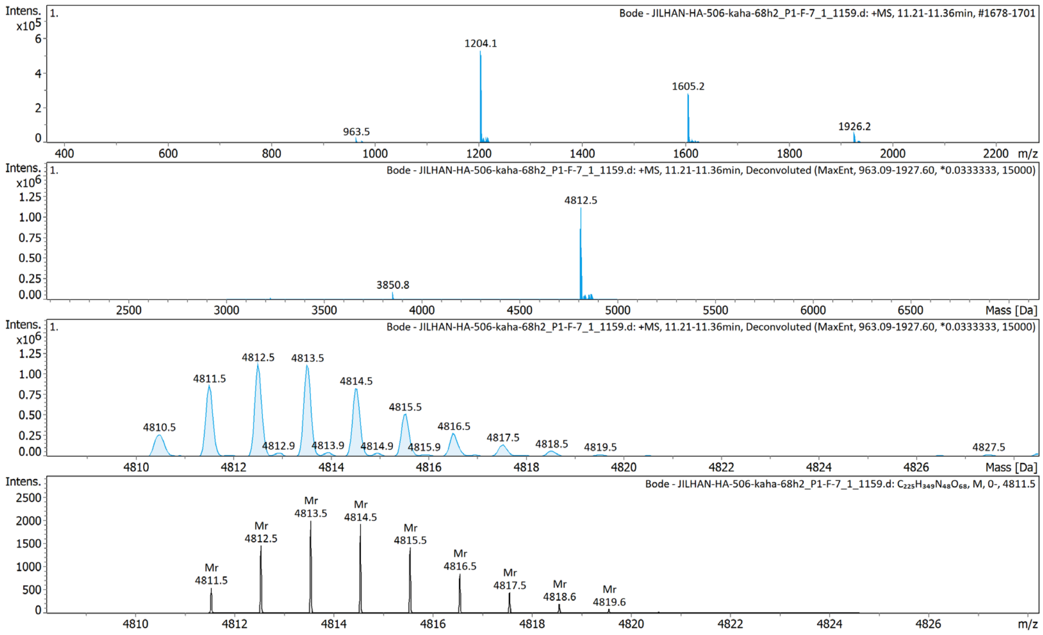

7. NMR Spectra

Allyl (*tert*-butoxycarbonyl)-*L*-alloisoleucylphenylalaninate (S2)

^{1}H NMR

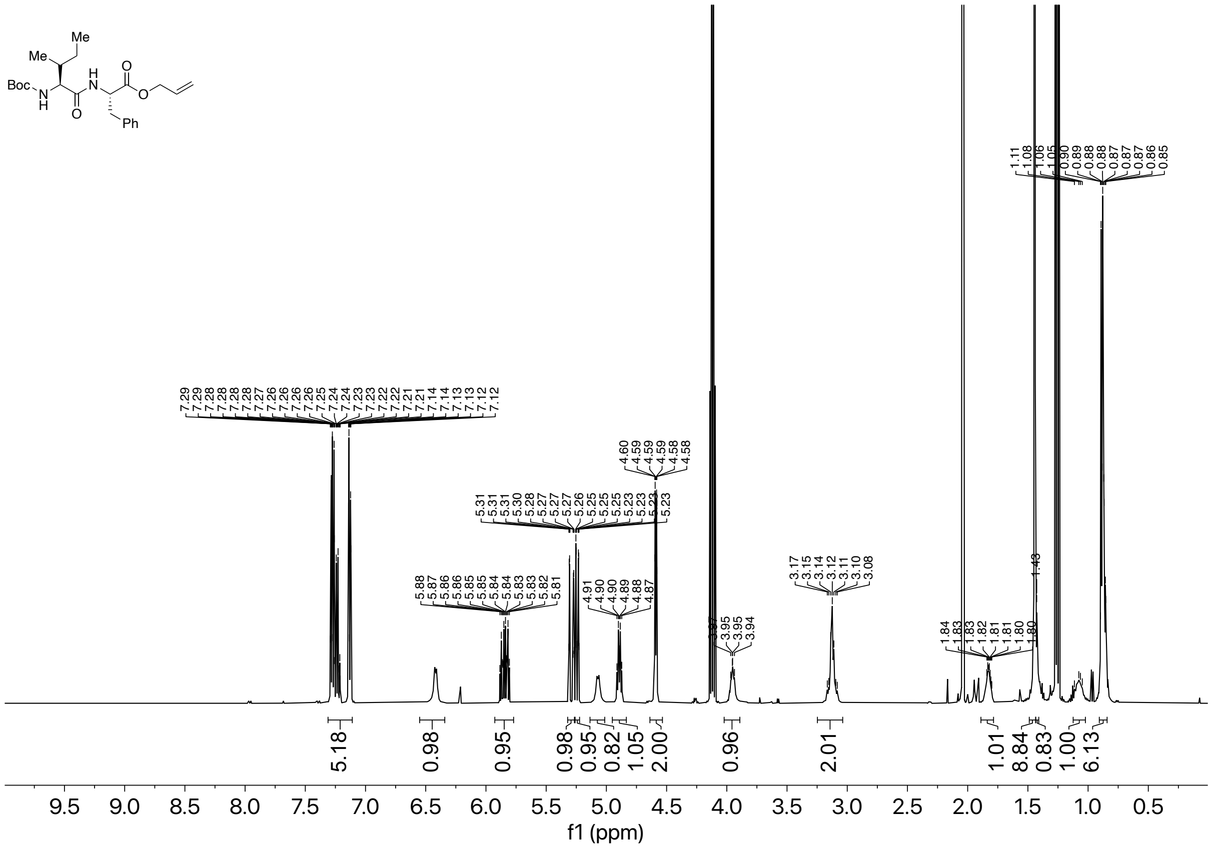

^{13}C NMR

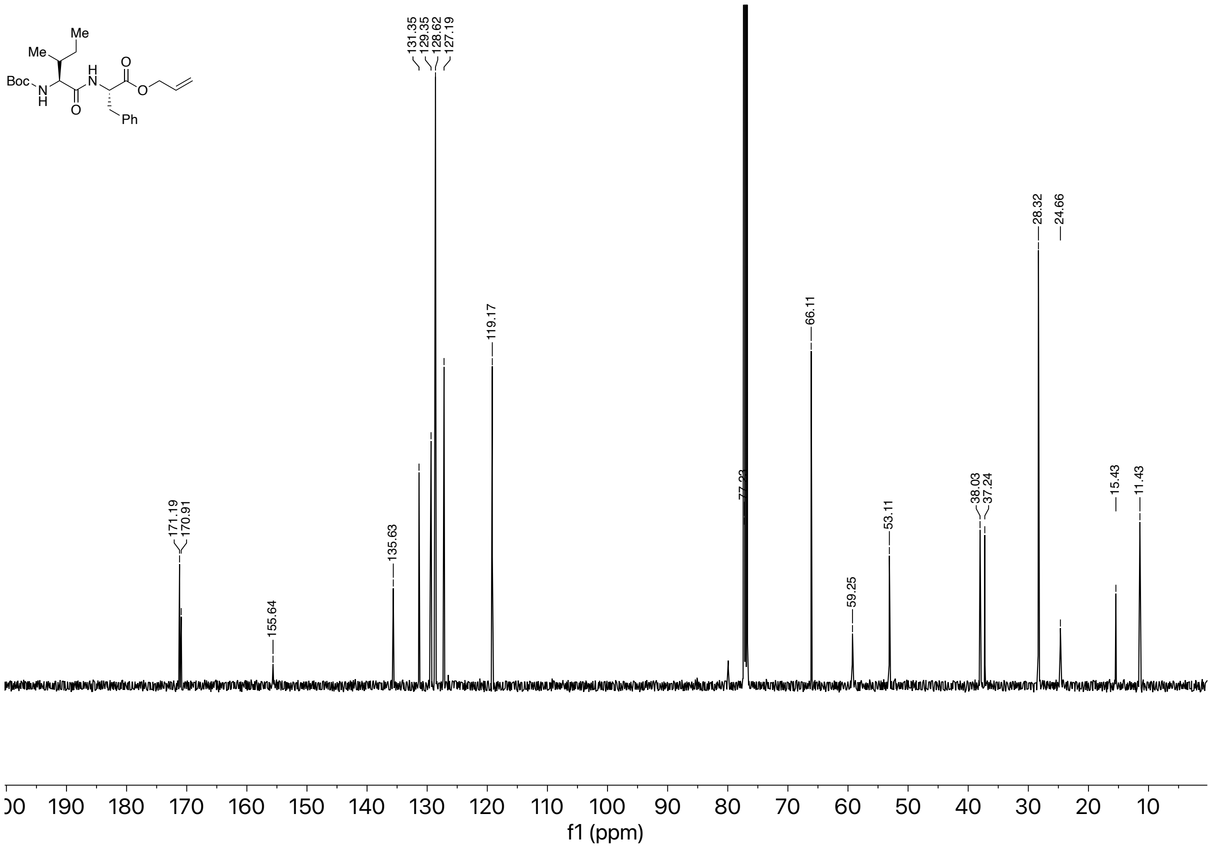

Allyl (cyanomethyl)-*L*-alloisoleucyl-*L*-phenylalaninate (S3)

^{1}H NMR

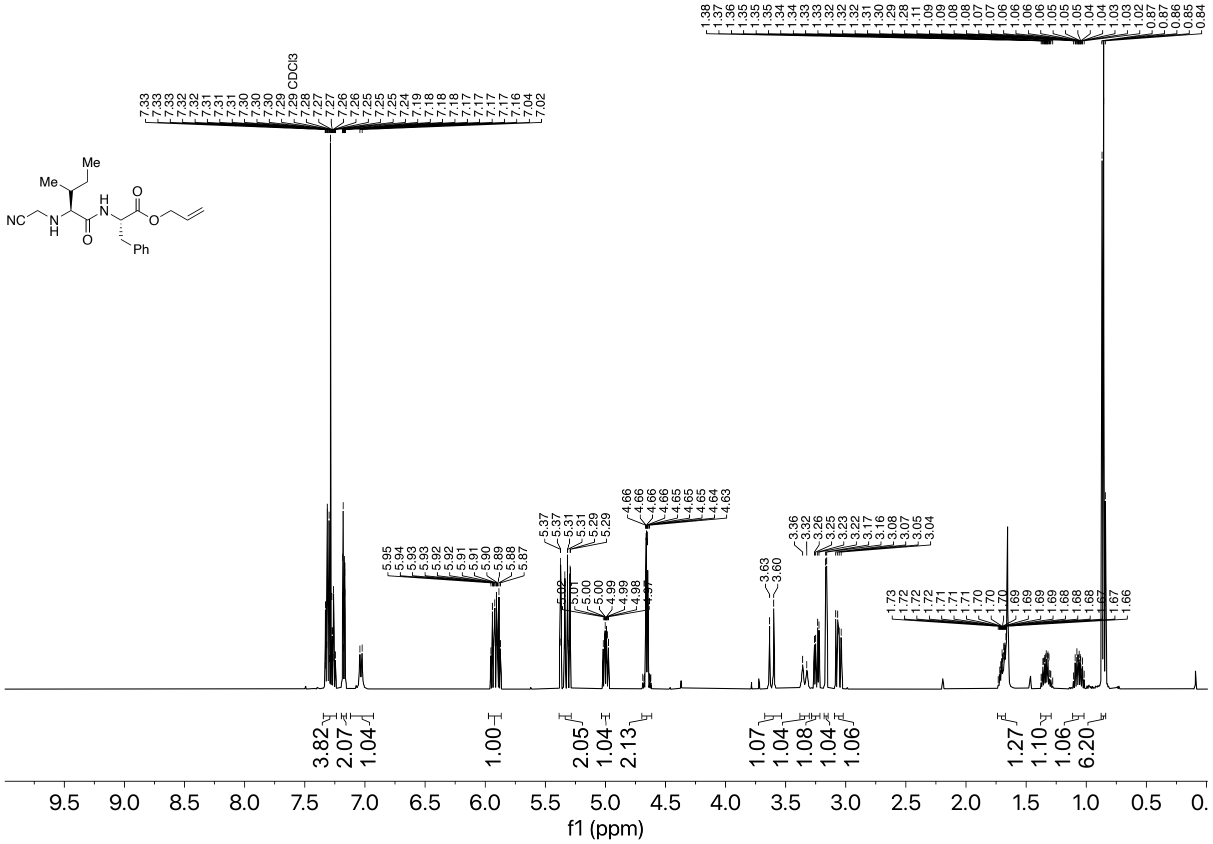

^{13}C NMR

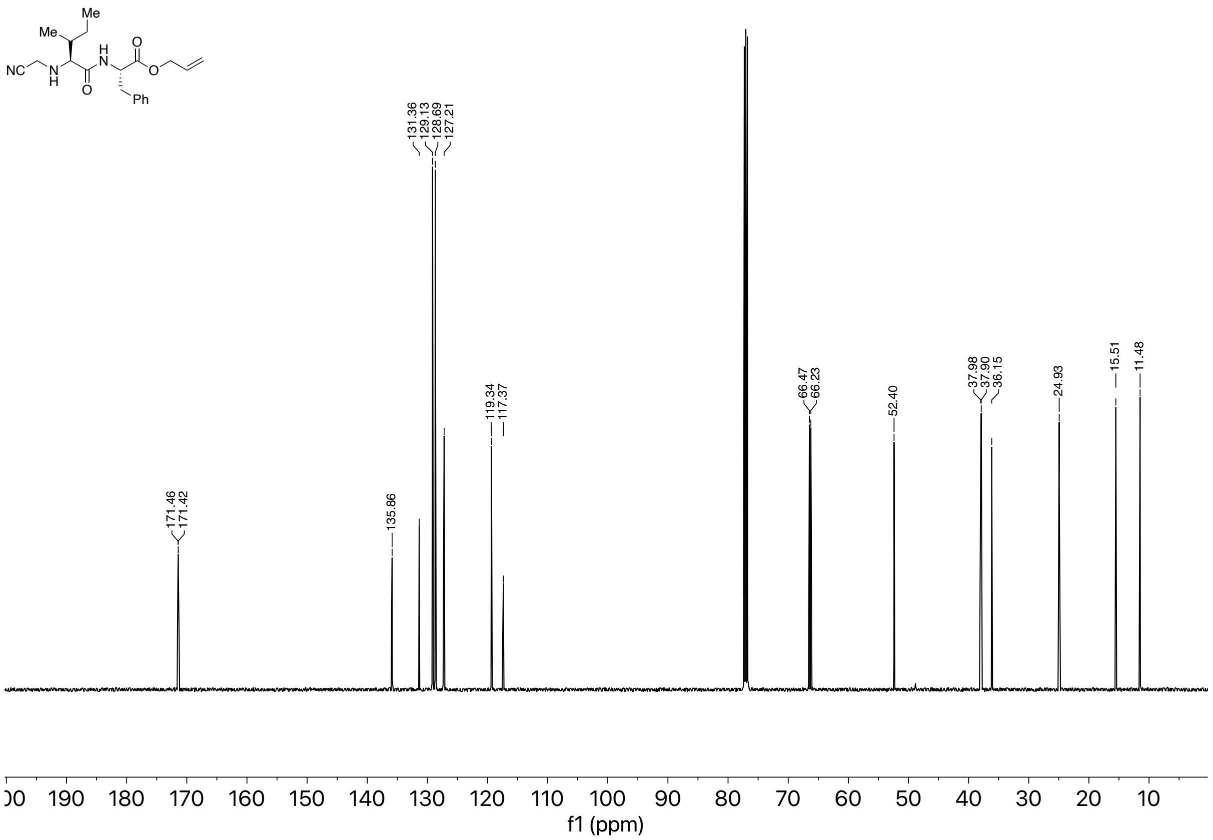

Allyl hydroxy-*L*-alloisoleucyl-*L*-phenylalaninate (2)

^{1}H NMR

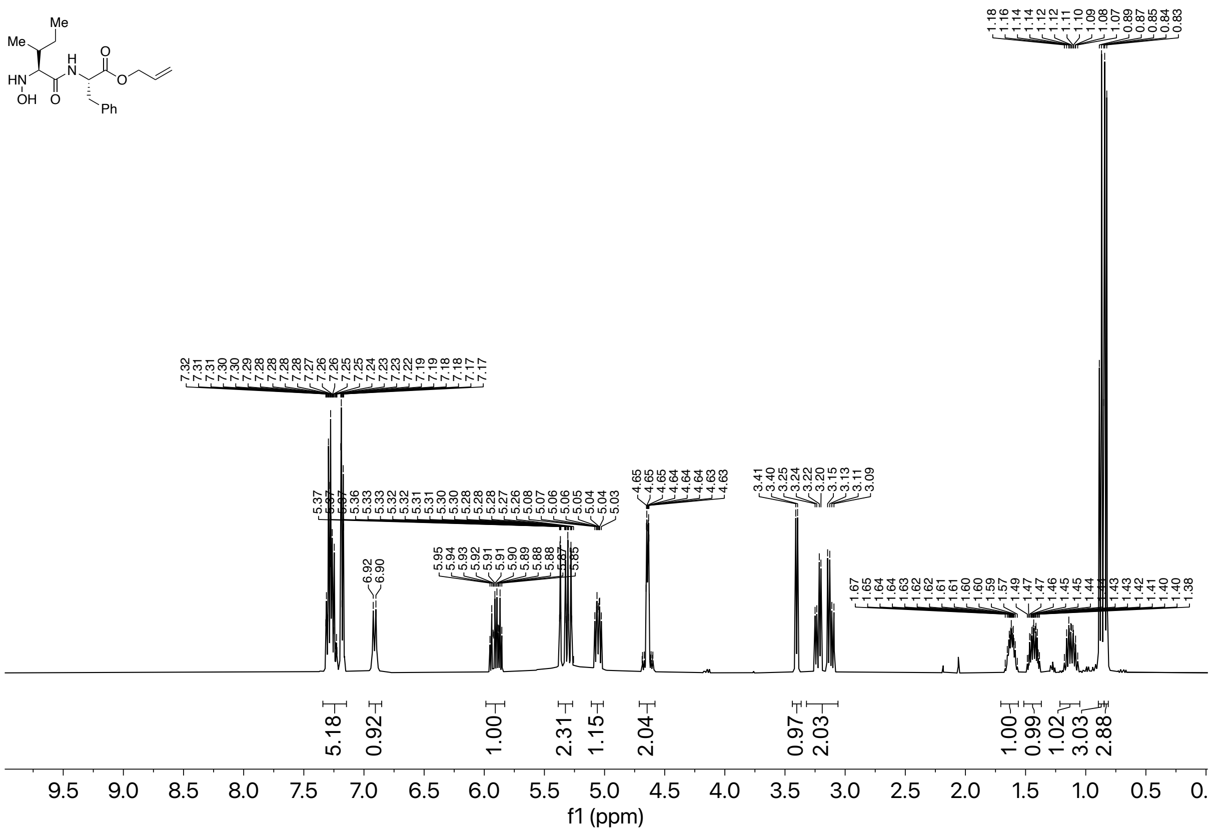

^{13}C NMR

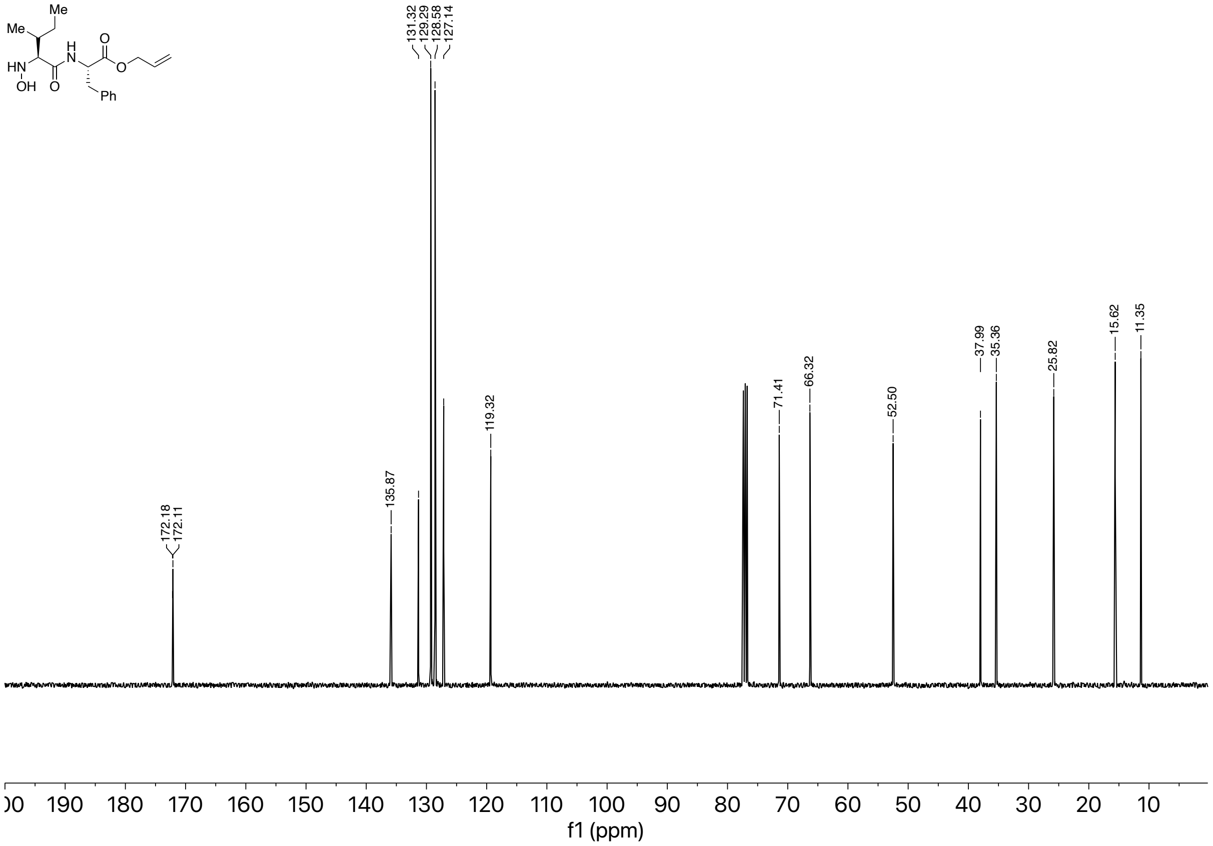

Allyl *N*-hydroxy-*N*-(((*S*)-1-(2-nitrophenyl)ethoxy)carbonyl)-*L*-alloisoleucyl-*L*-phenylalaninate (4)

^{1}H NMR

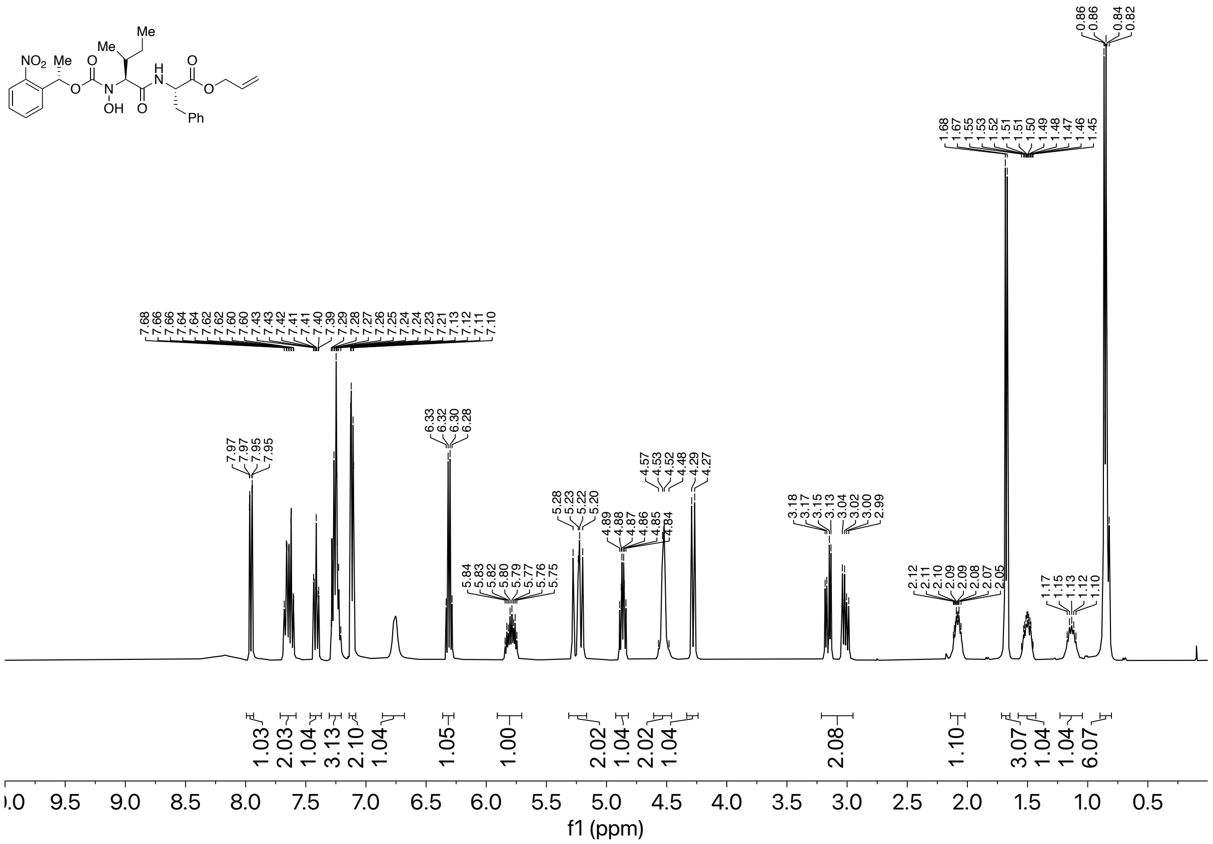

^{13}C NMR

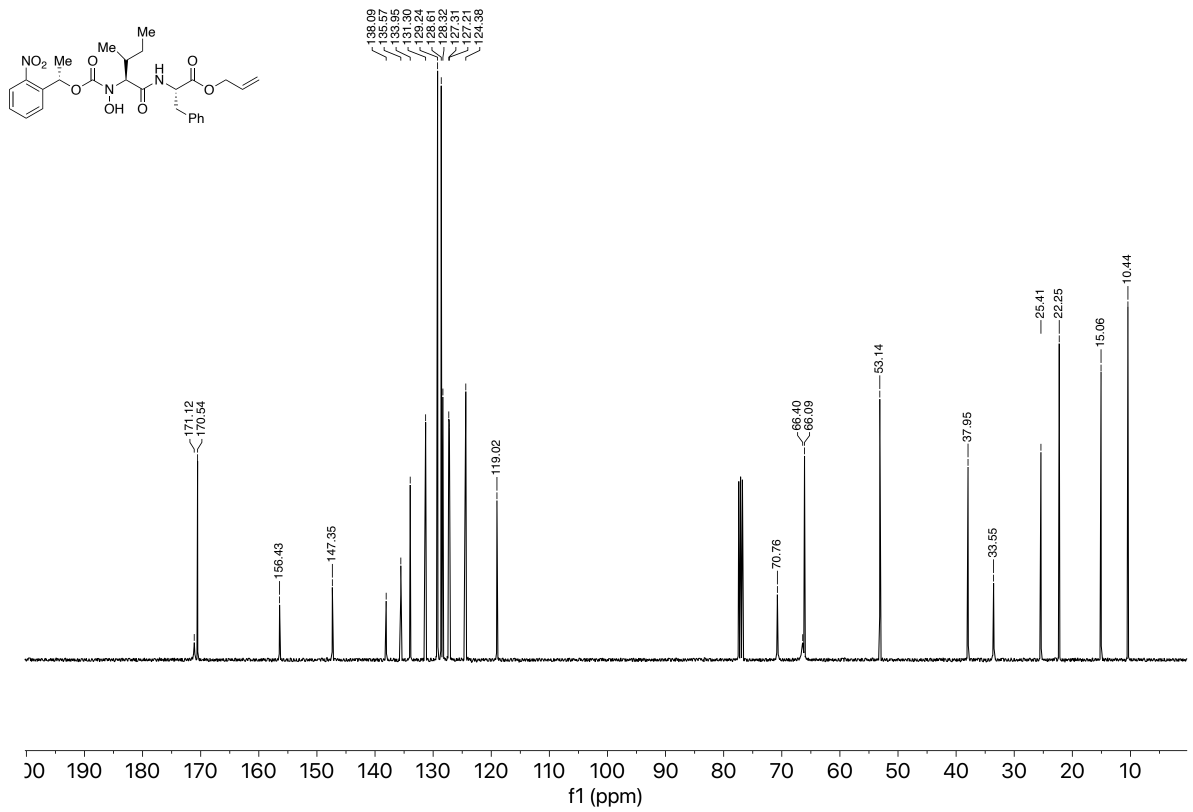

Allyl N-(((4-nitrophenoxy)carbonyl)oxy)-N-(((S)-1-(2-nitrophenyl)ethoxy)carbonyl)-L-alloisoleucyl-L-phenylalaninate (5)

^{1}H NMR

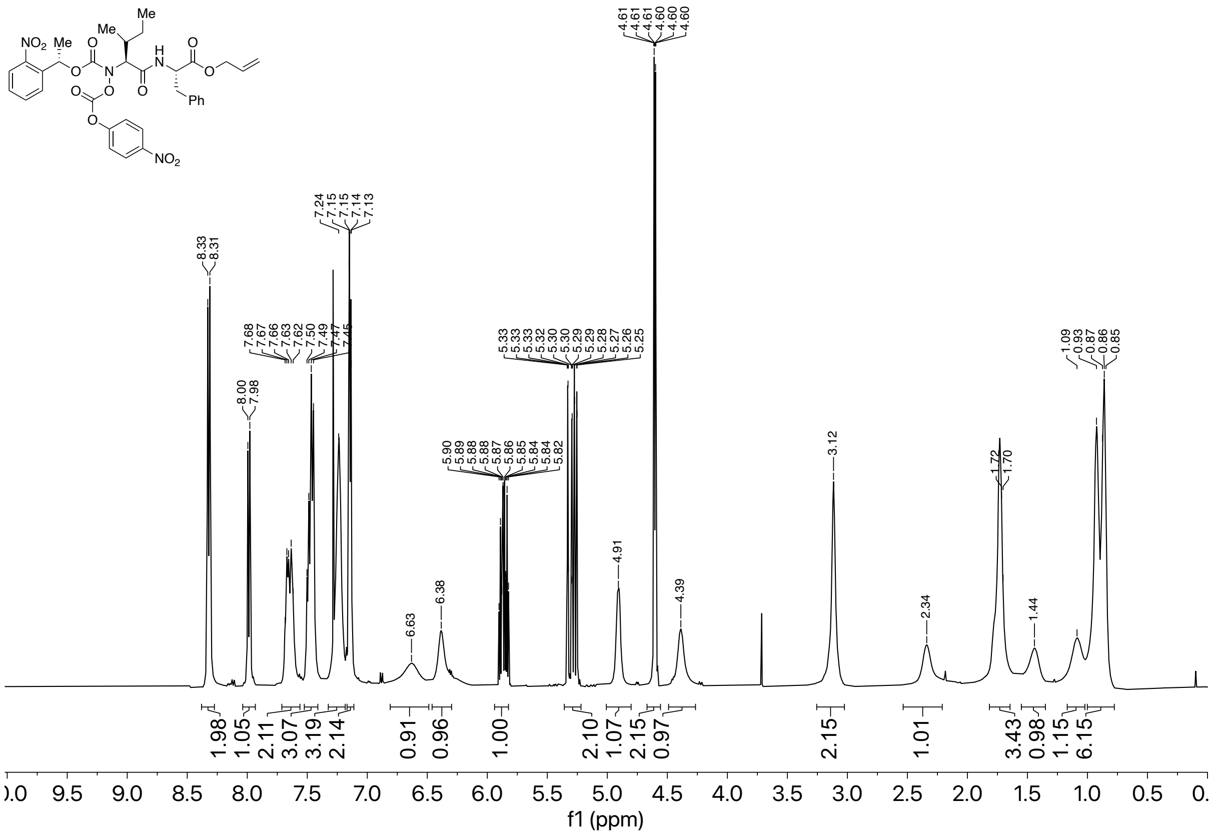

^{13}C NMR

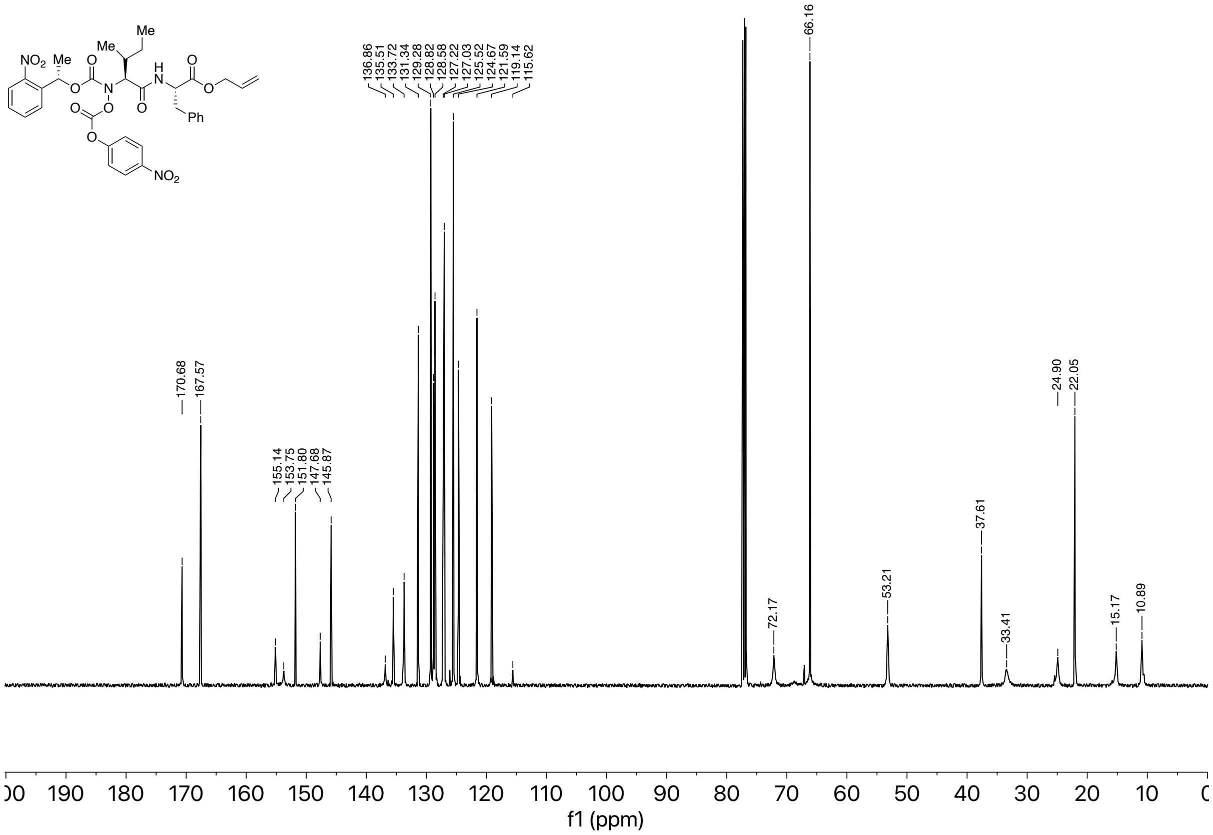

(*S*)-1-(2-nitrophenyl)ethyl (*S*)-5-((*S*)-1-(allyloxy)-1-oxo-3-phenylpropan-2-yl)-3-((*R*)-*sec*-butyl)-4,6-dioxo-1,2,5-oxadiazinane-2-carboxylate (6)

^{1}H NMR

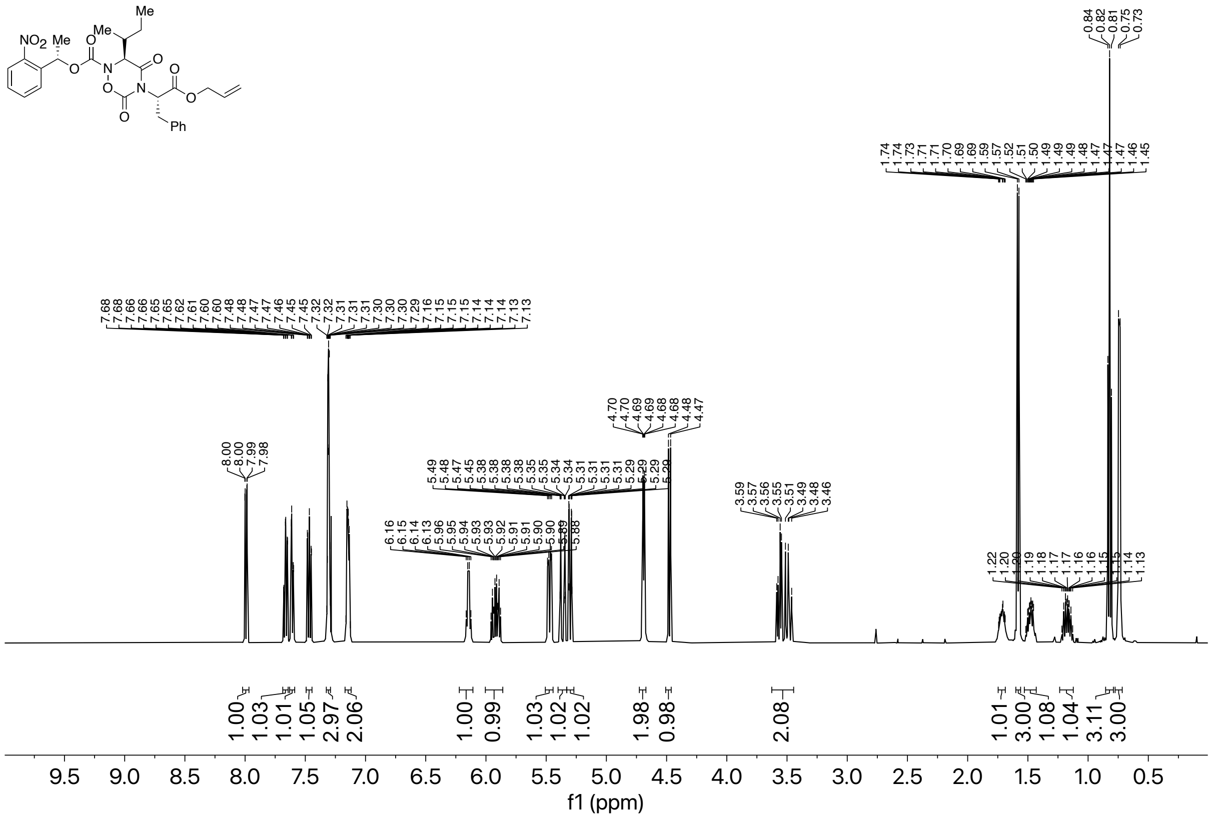

^{13}C NMR

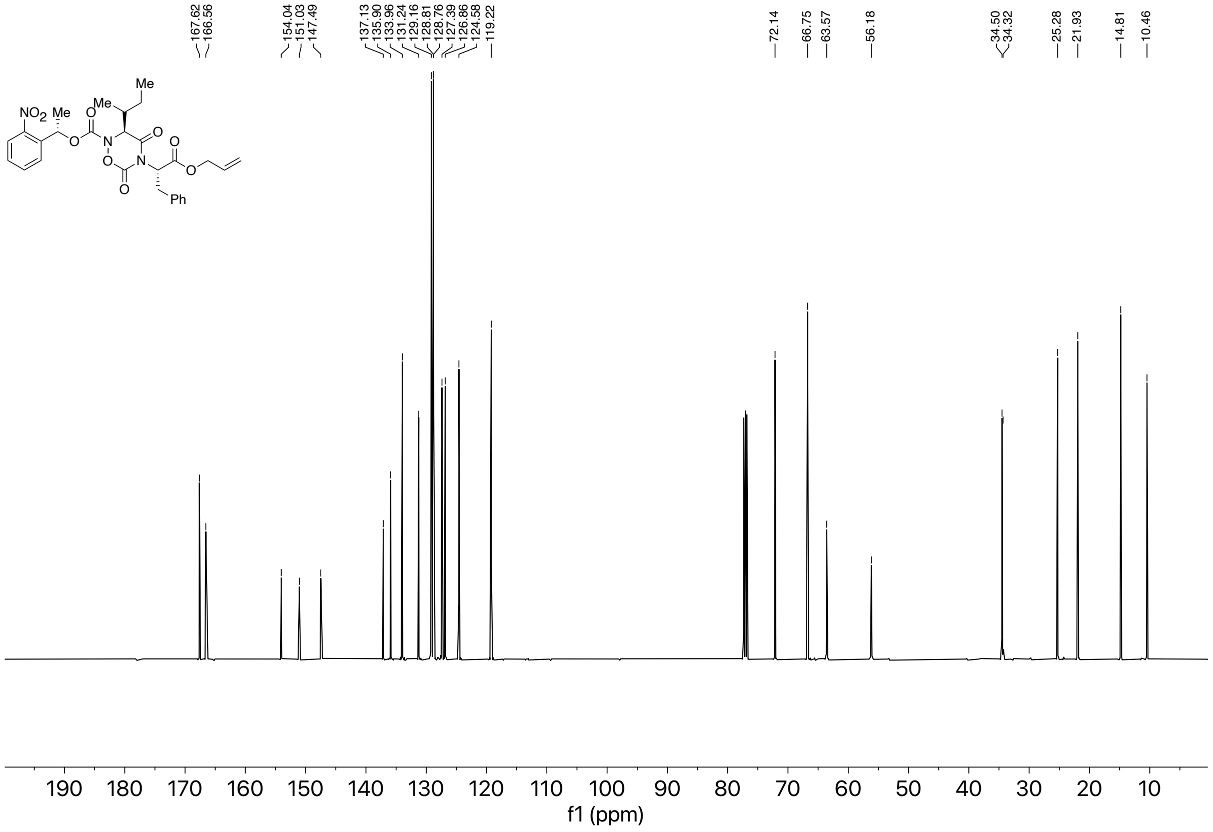

(*S*)-2-((*S*)-3-((*R*)-*sec*-butyl)-2-(((*S*)-1-(2-nitrophenyl)ethoxy)carbonyl)-4,6-dioxo-1,2,5-oxadiazinan-5-yl)-3-phenylpropanoic acid (7)

^{1}H NMR

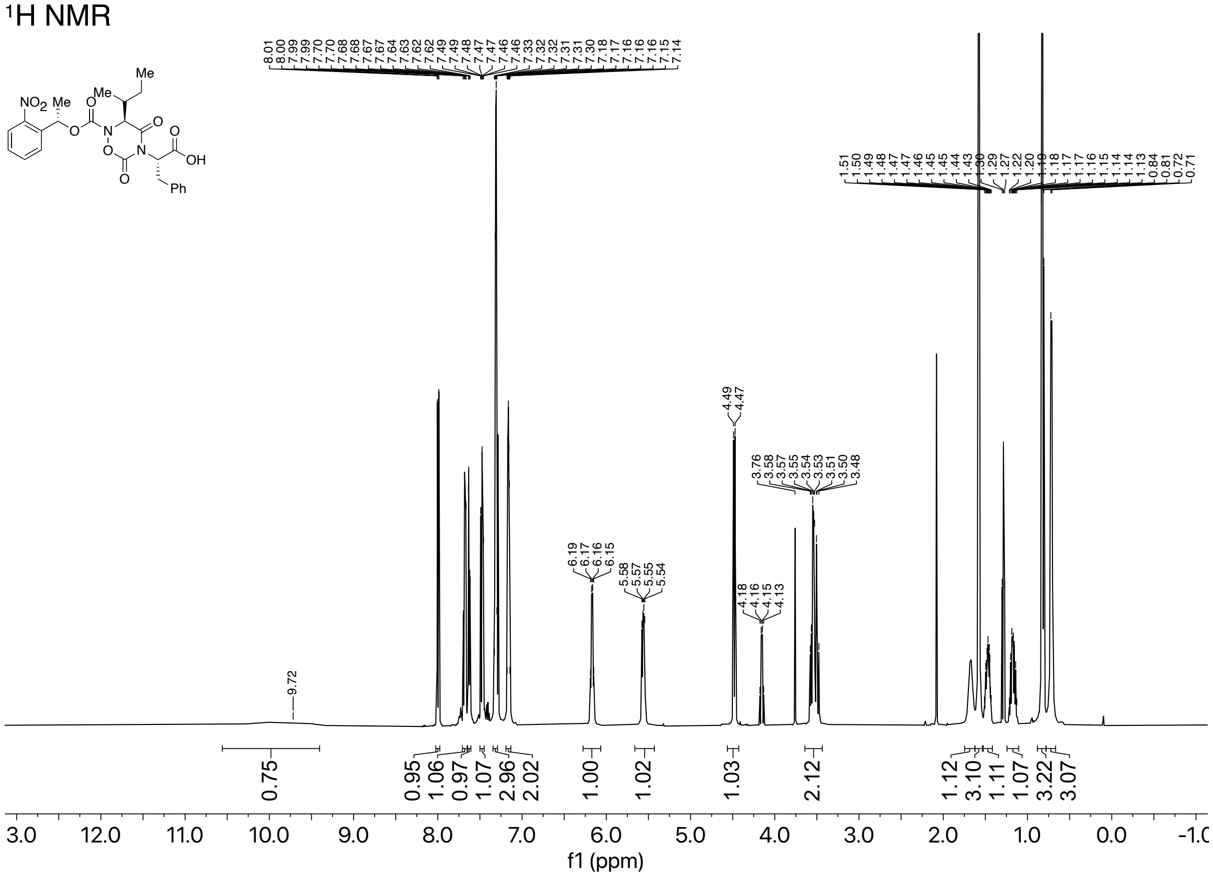

^{13}C NMR

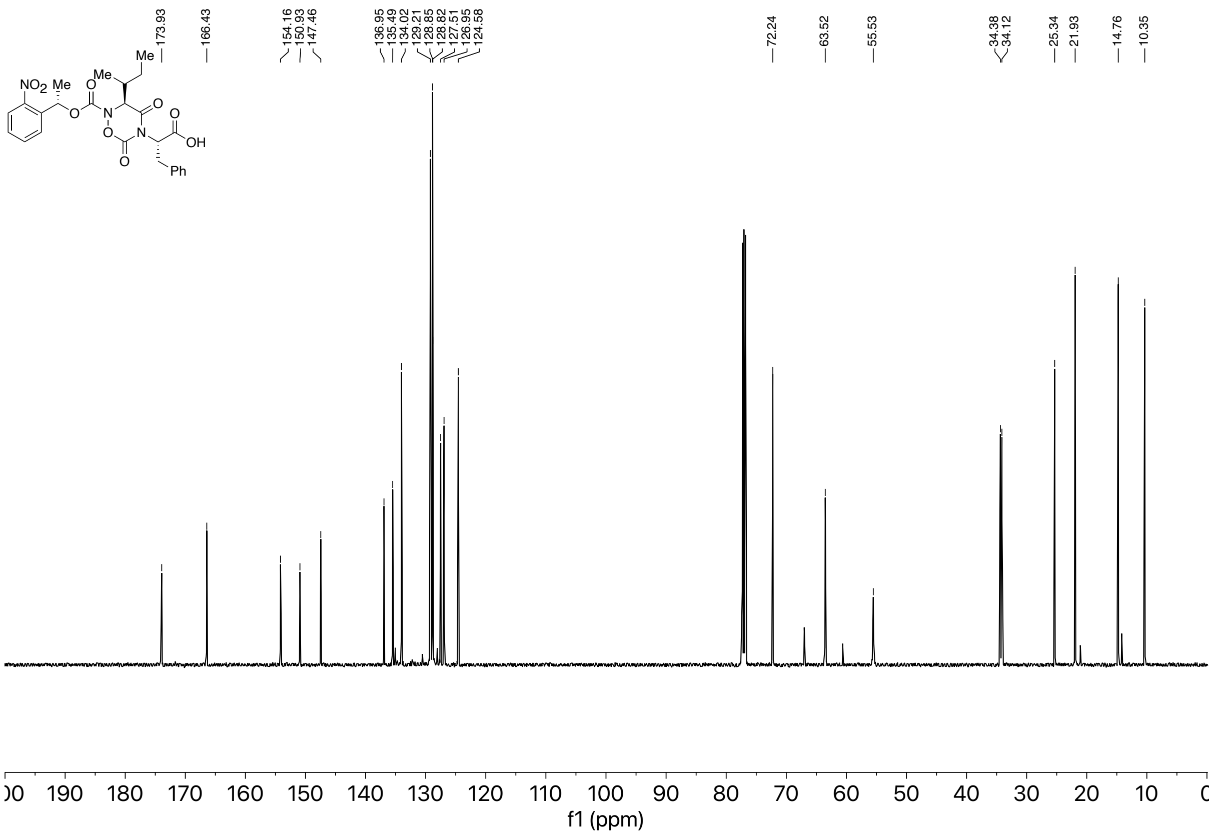

Allyl (*tert*-butoxycarbonyl)-*L*-alloisoleucyl-*L*-alaninate (S6)

^{1}H NMR

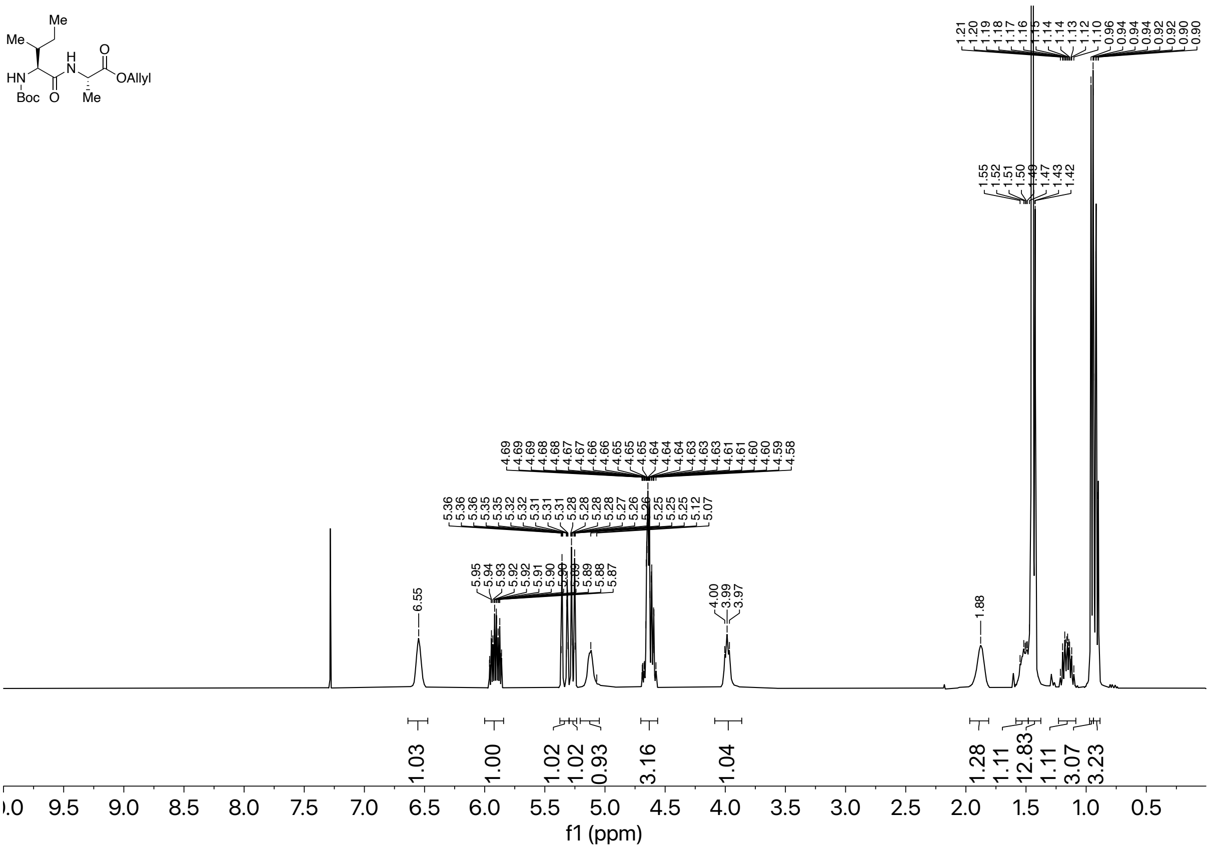

^{13}C NMR

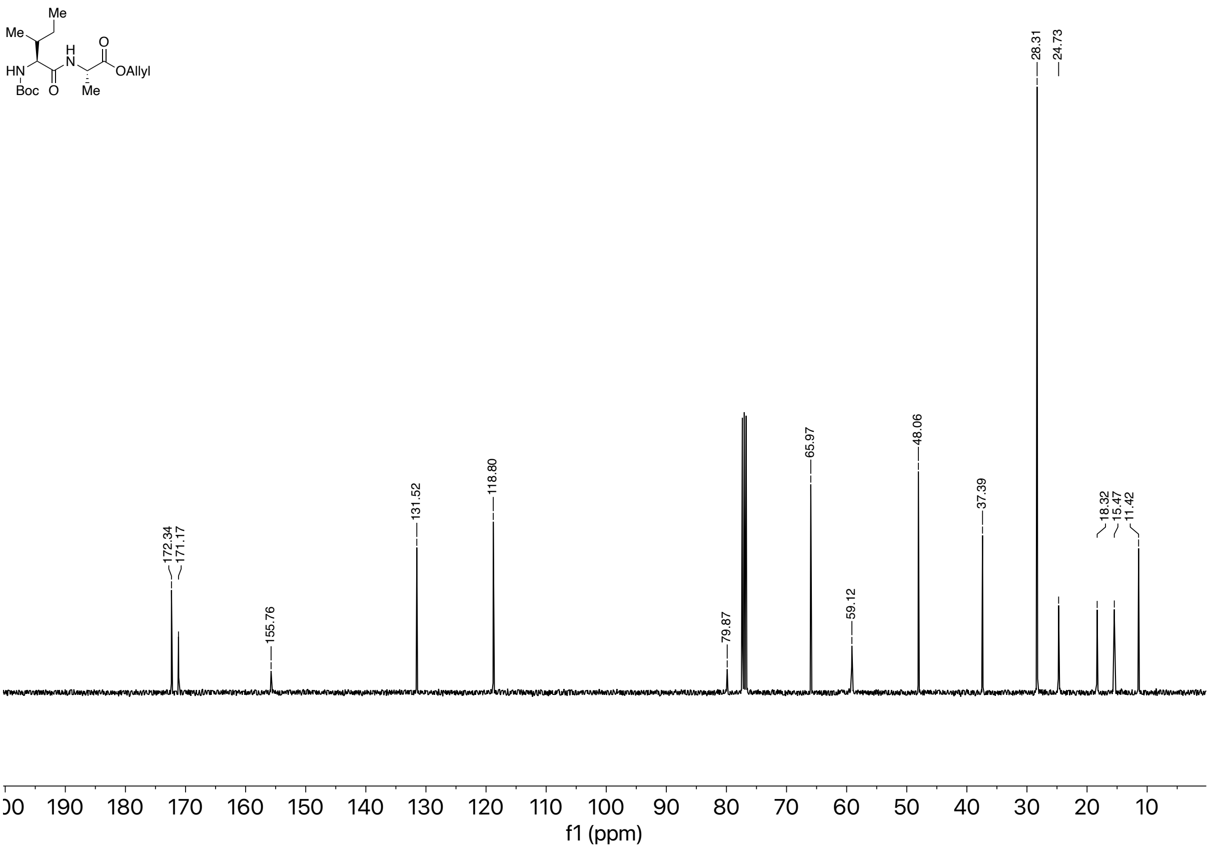

Allyl (cyanomethyl)-*L*-alloisoleucyl-*L*-alaninate (S7)

^{1}H NMR

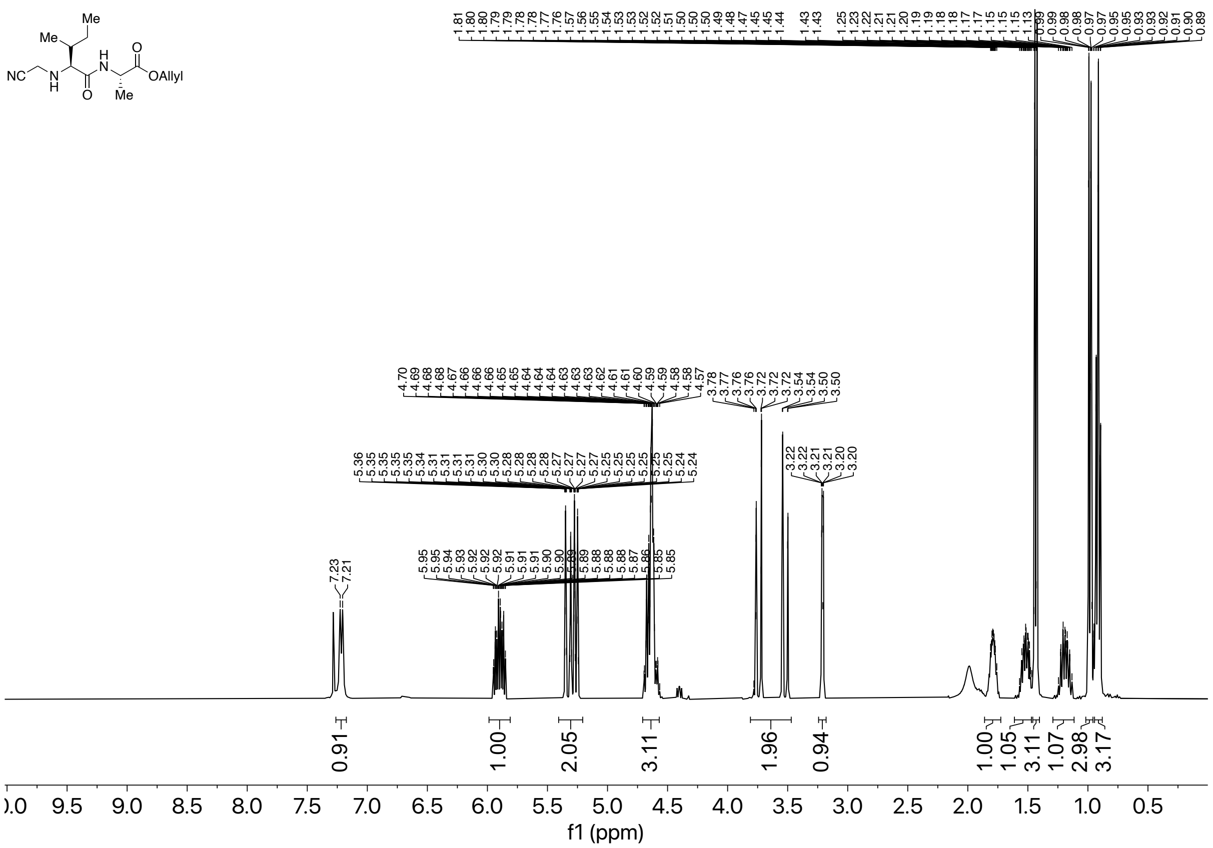

^{13}C NMR

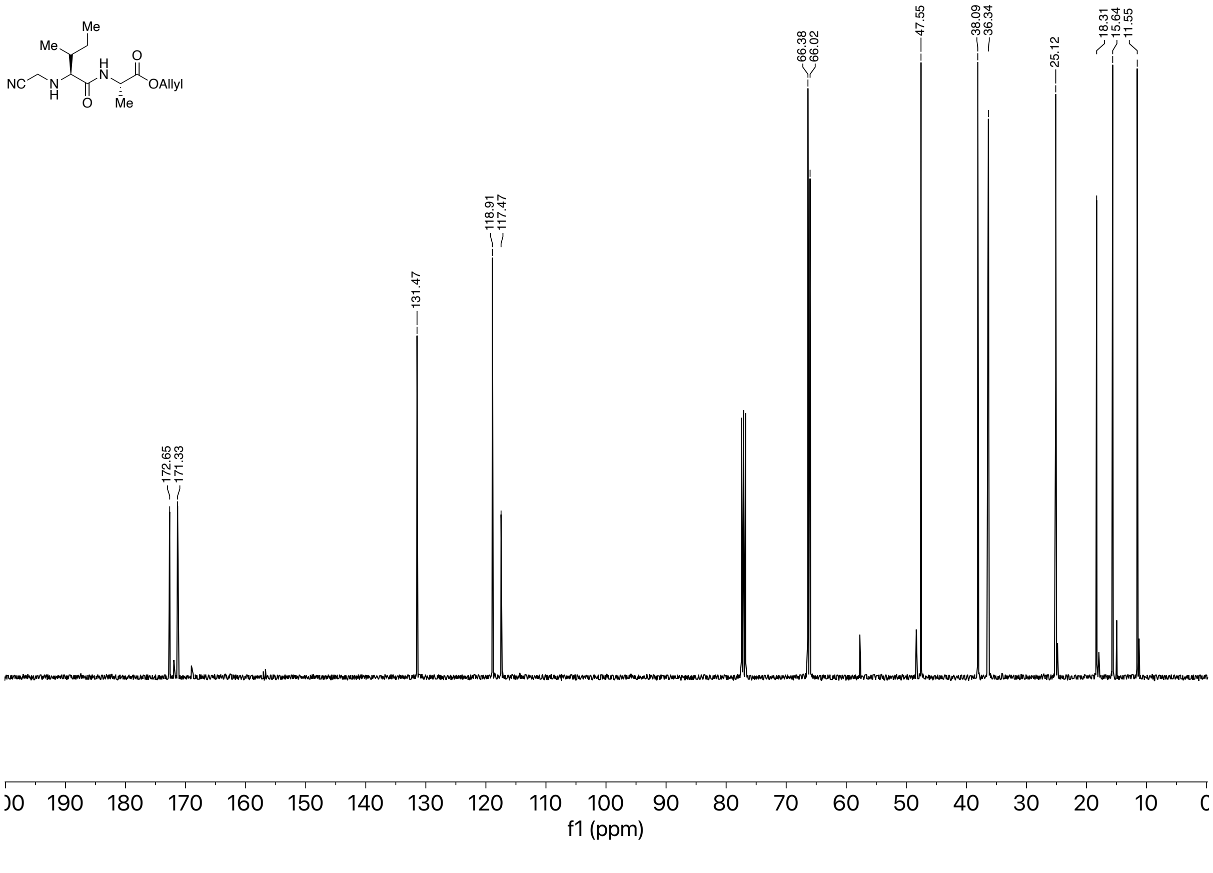

Allyl hydroxy-*L*-alloisoleucyl-*L*-alaninate (S10)

^{1}H NMR

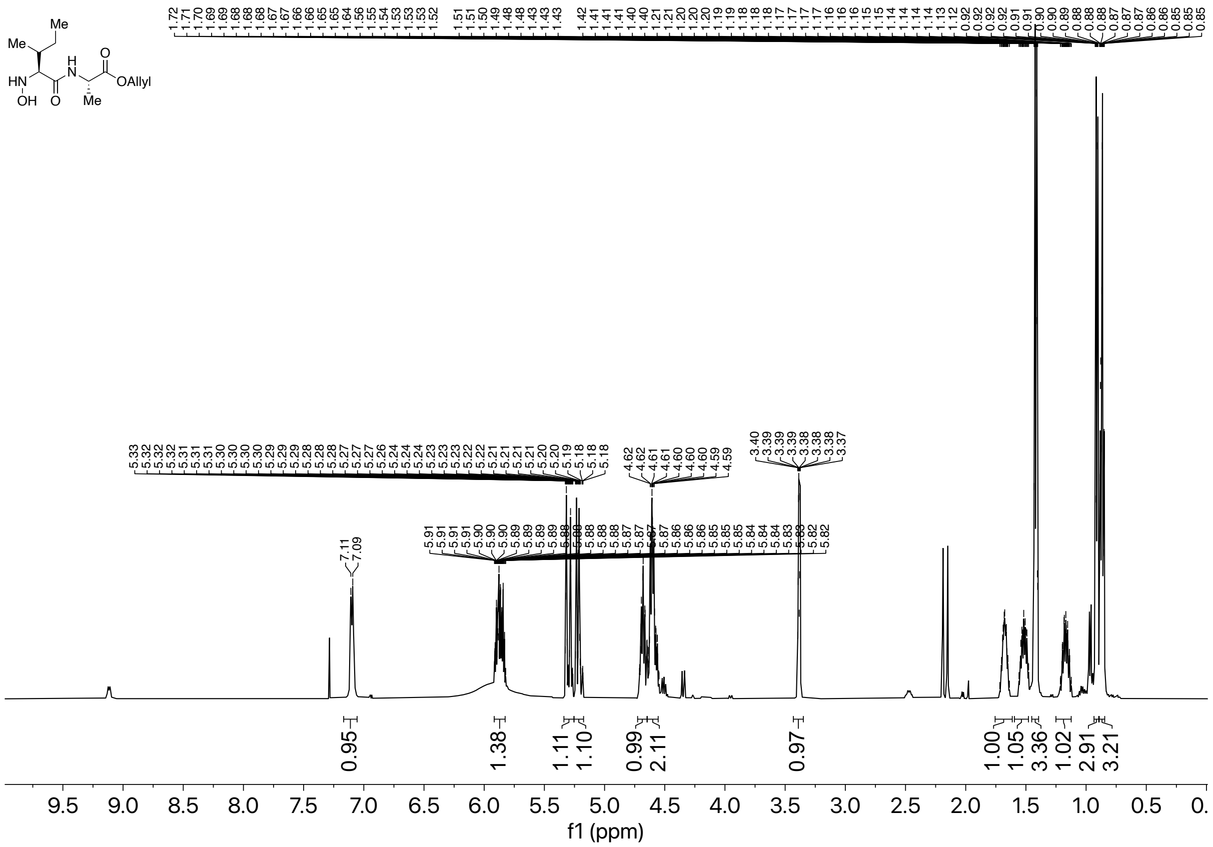

^{13}C NMR

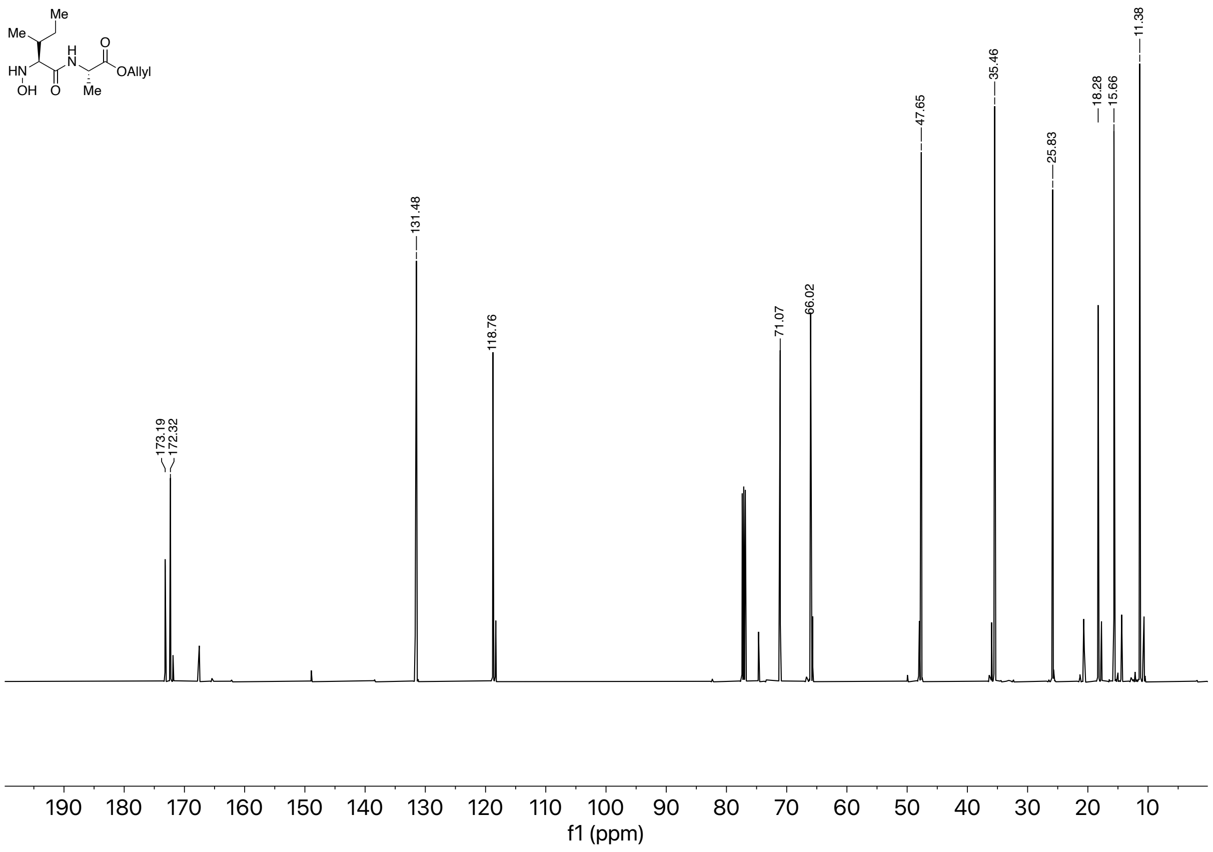

Allyl *N*-hydroxy-*N*-(((*S*)-1-(2-nitrophenyl)ethoxy)carbonyl)-*L*-alloisoleucyl-*L*-alaninate (S11)

^{1}H NMR

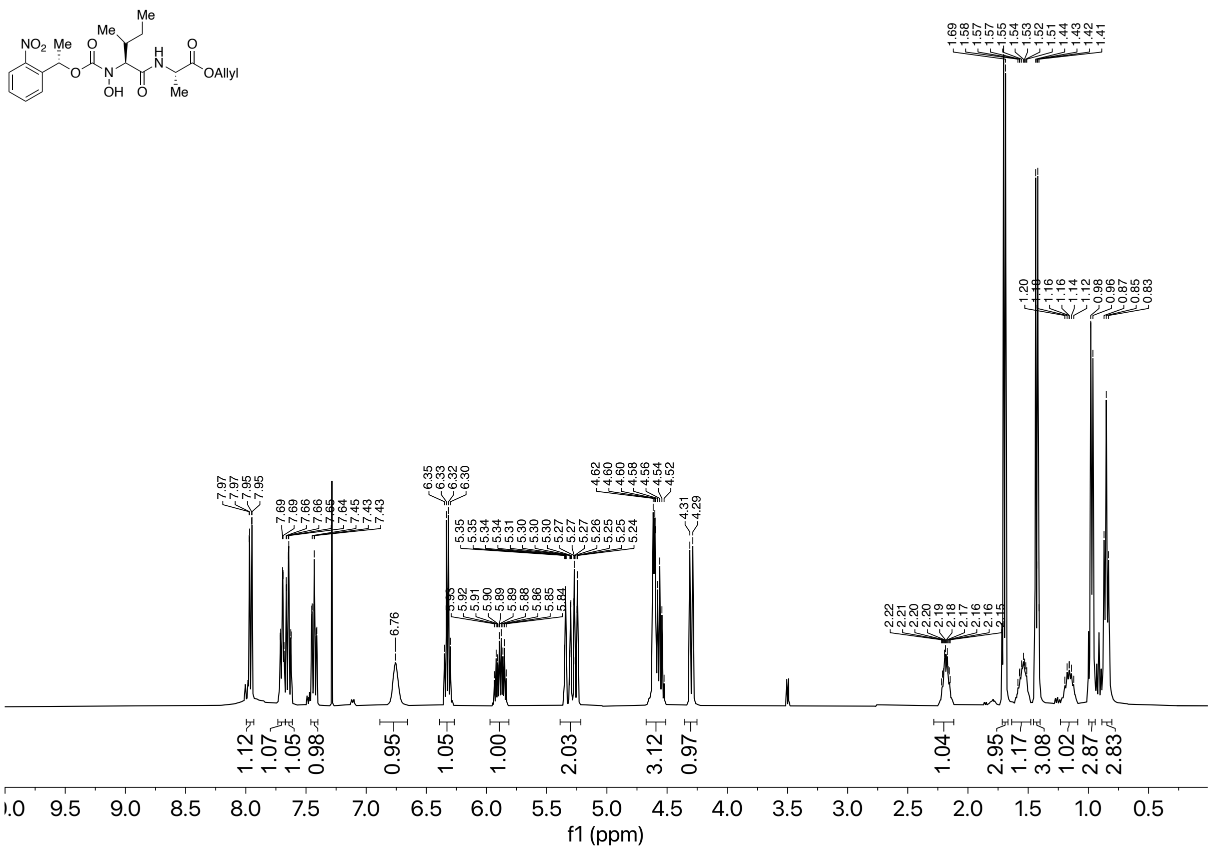

^{13}C NMR

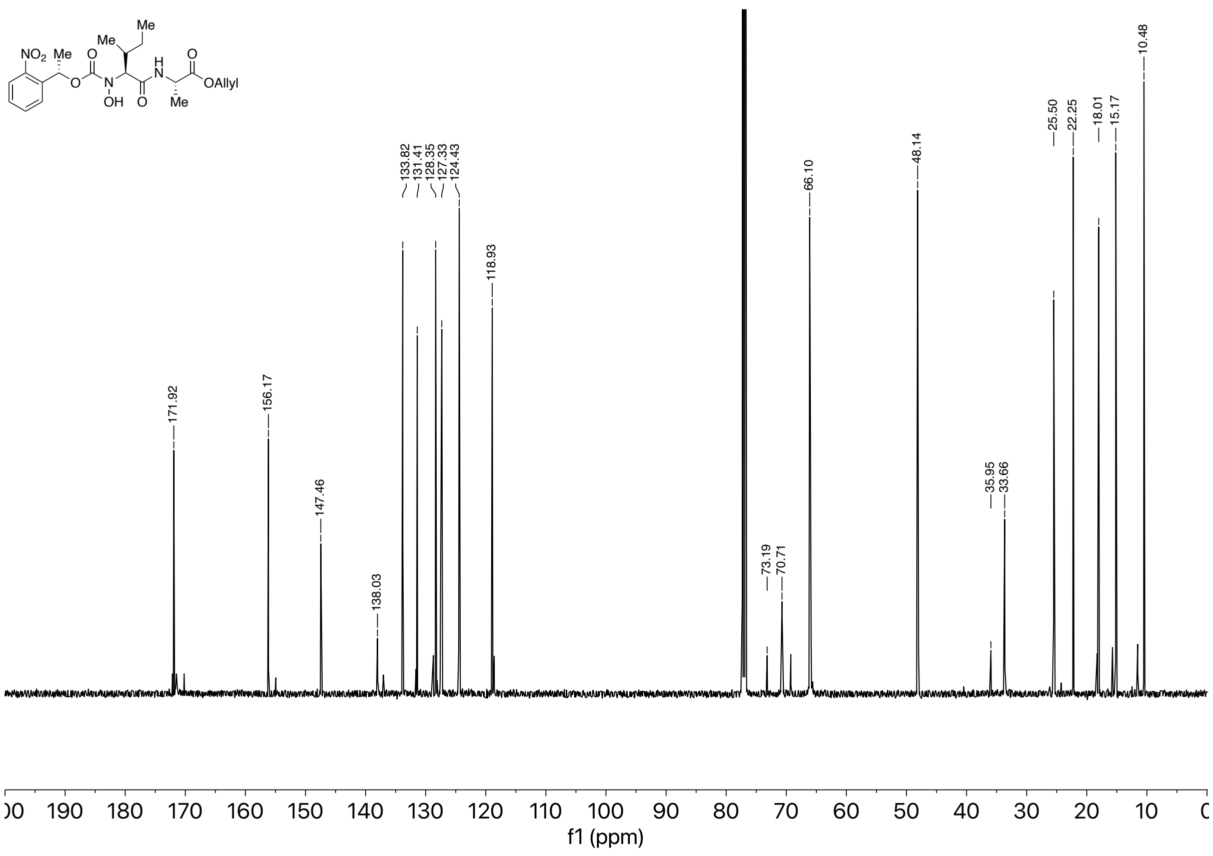

Allyl *N*-(((4-nitrophenoxy)carbonyl)oxy)-*N*-(((*S*)-1-(2-nitrophenyl)ethoxy)carbonyl)-*L*-alloisoleucyl-*L*-alaninate (S12)

^{1}H NMR

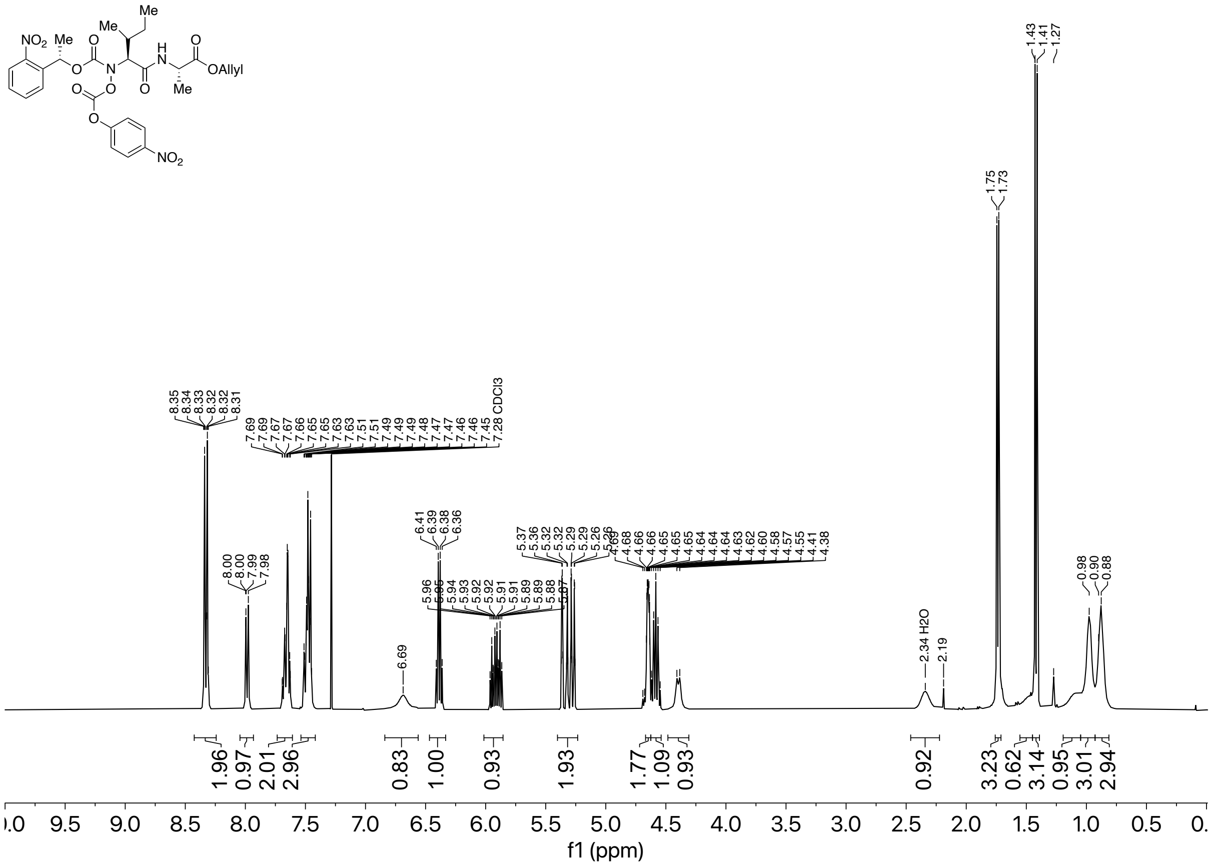

^{13}C NMR

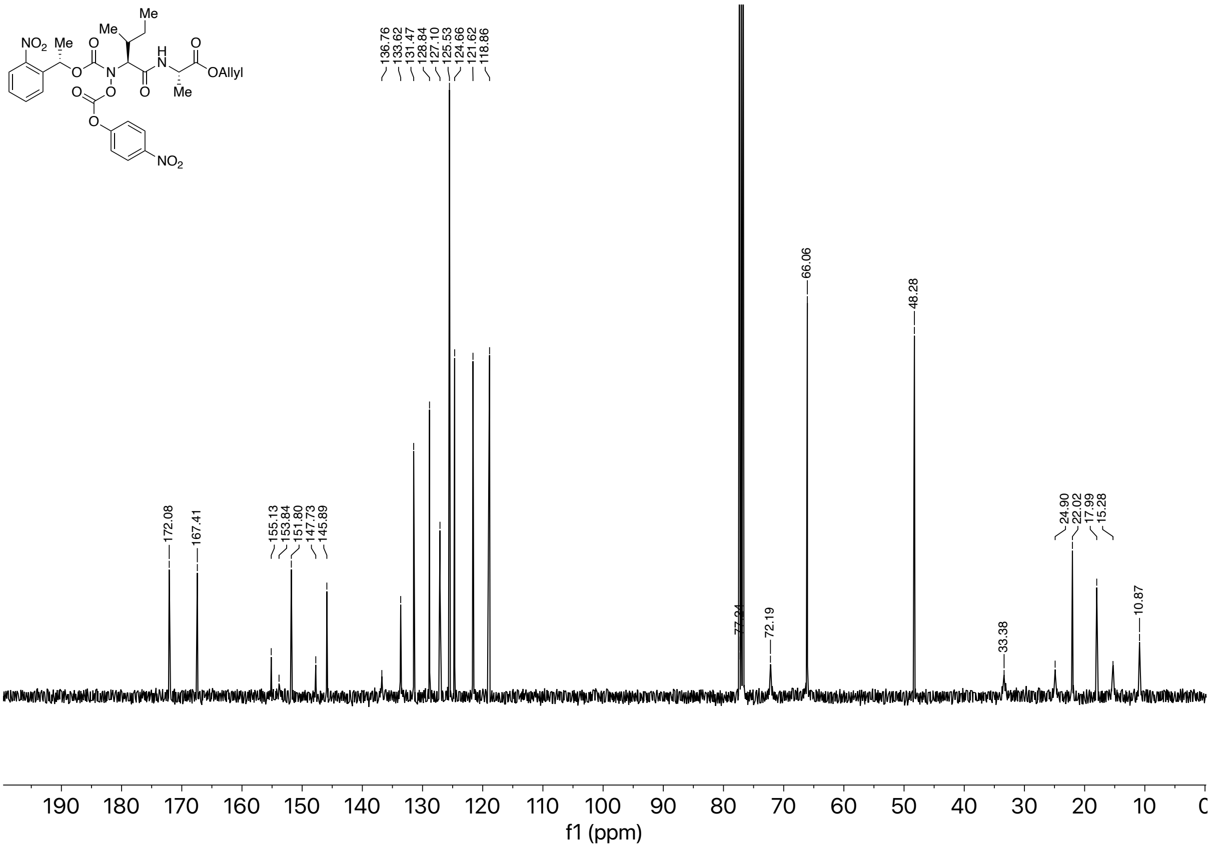

(*S*)-1-(2-nitrophenyl)ethyl (*S*)-5-((*S*)-1-(allyloxy)-1-oxopropan-2-yl)-3-((*R*)-*sec*-butyl)-4,6-dioxo-1,2,5-oxadiazinane-2-carboxylate (S13)

^{1}H NMR

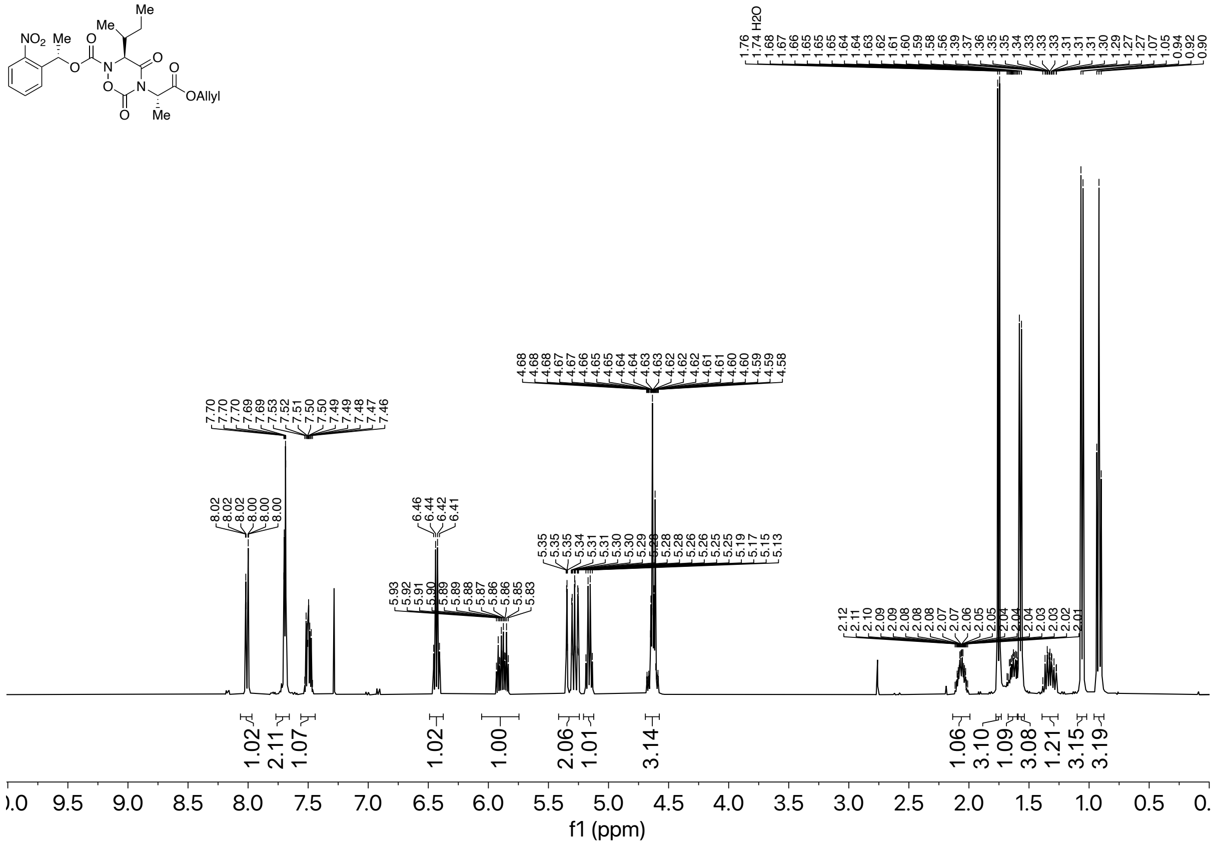

^{13}C NMR

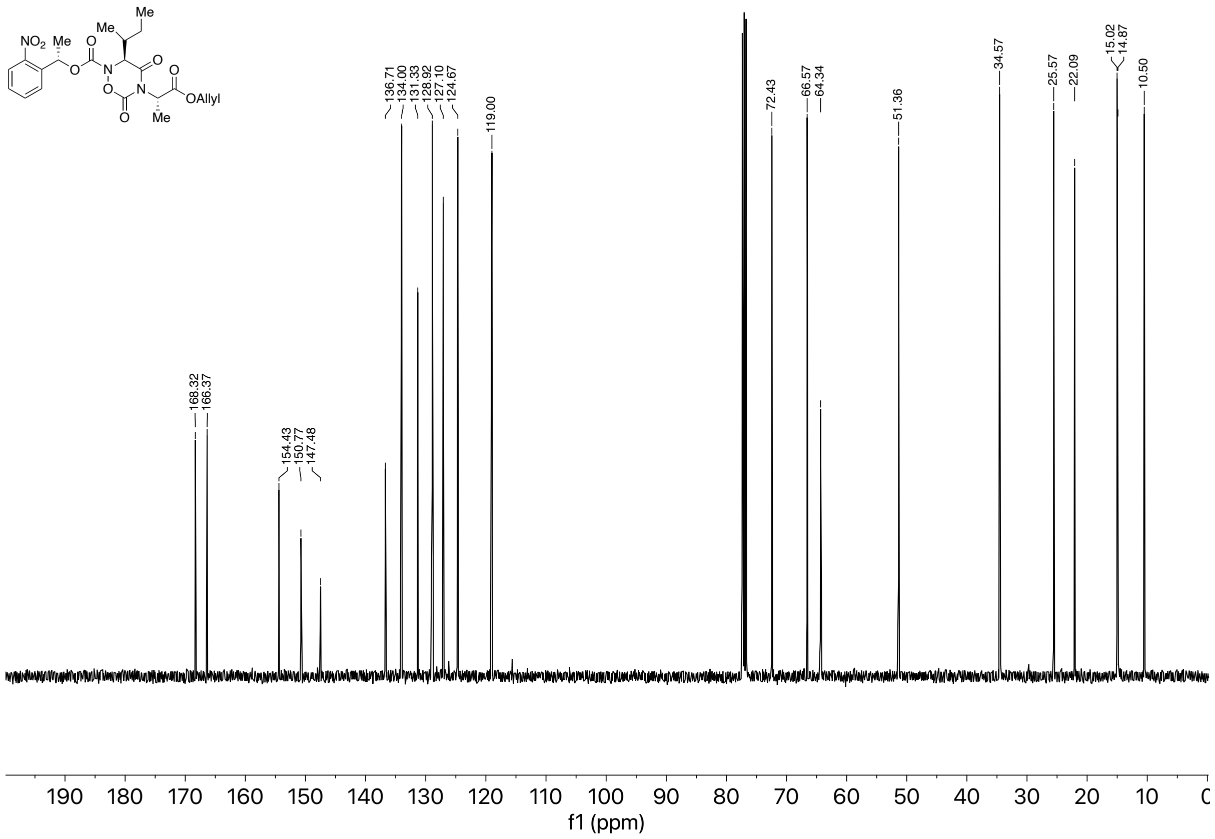

(*S*)-2-((*S*)-3-((*R*)-*sec*-butyl)-2-(((*S*)-1-(2-nitrophenyl)ethoxy)carbonyl)-4,6-dioxo-1,2,5-oxadiazinan-5-yl)propanoic acid (11)

^{1}H NMR

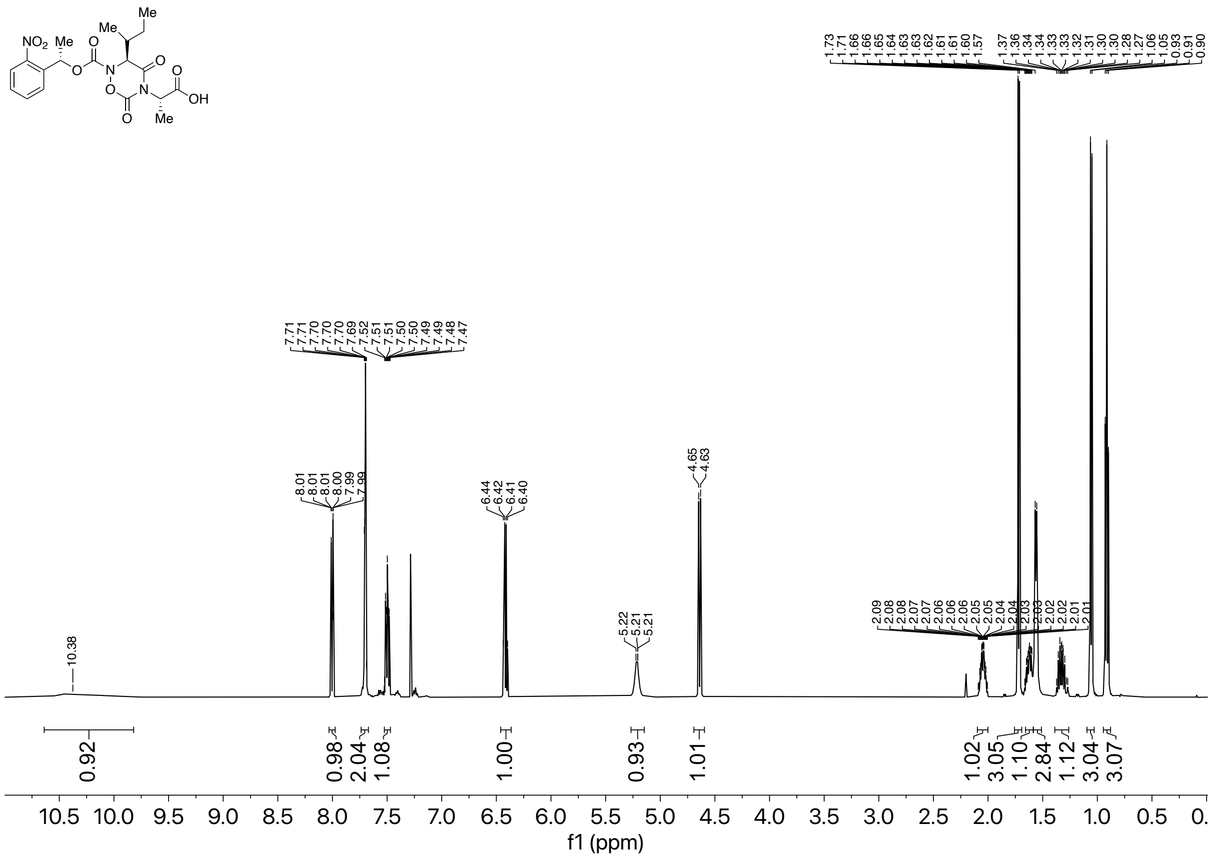

^{13}C NMR

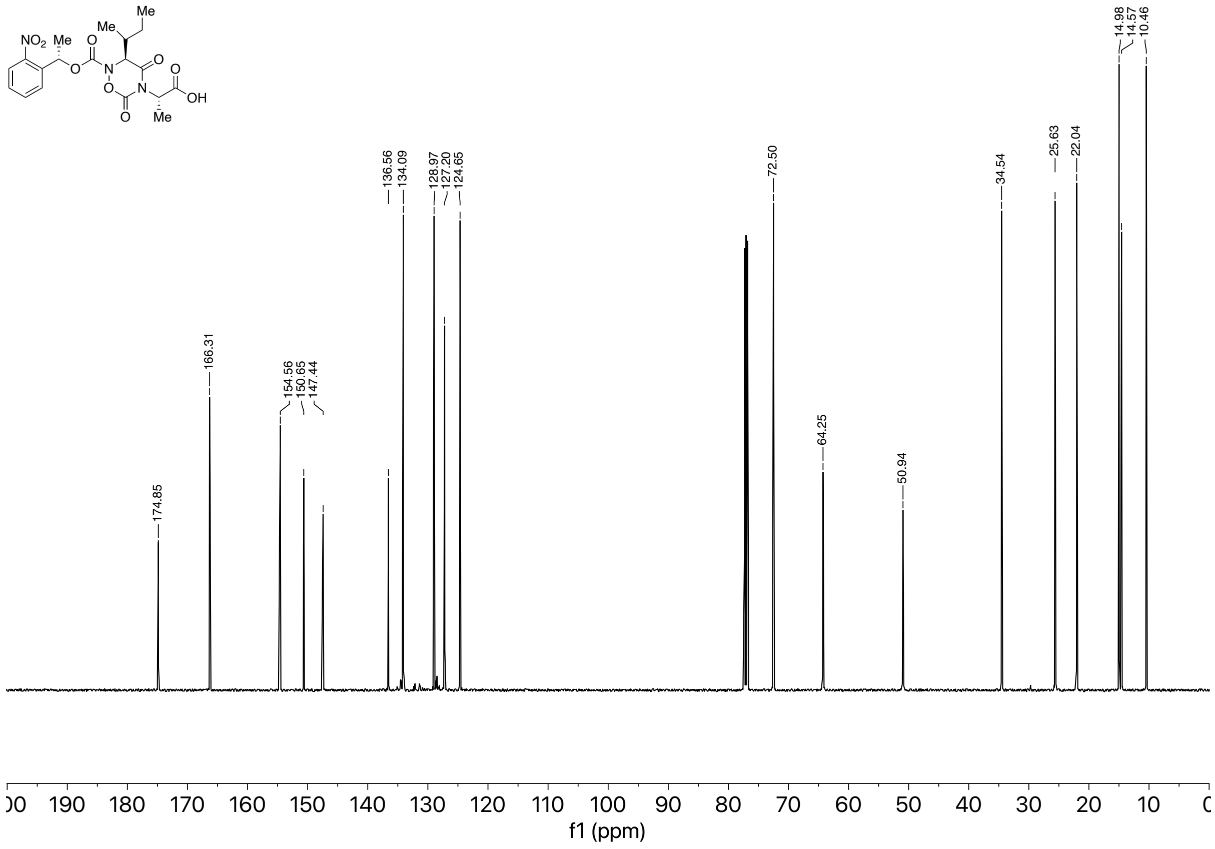

8. References

(1) Tokuyama, H.; Kuboyama, T.; Amano, A.; Yamashita, T.; Fukuyama, T. A Novel Transformation of Primary Amines to N-Monoalkylhydroxylamines. *Synthesis* **2000**, *2000* (09), 1299–1304.

(2) Odaka, M.; Furuta, T.; Kobayashi, Y.; Iwamura, M. Synthesis, Photoreactivity and Cytotoxic Activity of Caged Compounds of L-Leucyl-L-Leucine Methyl Ester, an Apoptosis Inducer. *Photochem. Photobiol.* **1996**, *63* (6), 800–806.

(3) Lacoursiere, R. E.; Shaw, G. S. Acetylated Ubiquitin Modulates the Catalytic Activity of the E1 Enzyme Uba1. *Biochemistry* **2021**, *60* (16), 1276–1285.

(4) Wucherpfennig, T. G.; Pattabiraman, V. R.; Limberg, F. R. P.; Ruiz-Rodríguez, J.; Bode, J. W. Traceless Preparation of C-Terminal α-Ketoacids for Chemical Protein Synthesis by α-Ketoacid–Hydroxylamine Ligation: Synthesis of SUMO2/3. *Angew. Chem. Int. Ed.* **2014**, *53* (45), 12248–12252.

(5) Mikami, T.; Majima, S.; Song, H.; Bode, J. W. Biocompatible Lysine Protecting Groups for the Chemoenzymatic Synthesis of K48/K63 Heterotypic and Branched Ubiquitin Chains. *ACS Cent. Sci.* **2023**, *9* (8), 1633–1641.

(6) Thuaud, F.; Rohrbacher, F.; Zwicky, A.; Bode, J. W. Incorporation of Acid-Labile Masking Groups for the Traceless Synthesis of C-Terminal Peptide α-Ketoacids. *Org. Lett.* **2016**, *18* (15), 3670–3673.
